# A feather hydrogen (δ^2^H) isoscape for Brazil

**DOI:** 10.1371/journal.pone.0271573

**Published:** 2022-08-03

**Authors:** Renata D. Alquezar, Fabio J. V. Costa, João Paulo Sena-Souza, Gabriela B. Nardoto, Keith A. Hobson

**Affiliations:** 1 Departamento de Ecologia, Instituto de Ciências Biológicas, Universidade de Brasília, Brasília, Distrito Federal, Brazil; 2 Department of Biology, University of Western Ontario, London, Ontario, Canada; 3 Instituto Nacional de Criminalística, Polícia Federal, Brasília, Distrito Federal, Brazil; 4 Departamento de Geociências, Universidade Estadual de Montes Claros, Montes Claros, Minas Gerais, Brazil; Universita del Salento, ITALY

## Abstract

Spatial patterns of stable isotopes in animal tissues or “isoscapes” can be used to investigate animal origins in a range of ecological and forensic investigations. Here, we developed a feather hydrogen isotope (δ^2^H_f_) isoscape for Brazil based on 192 samples of feathers from the family *Thraupidae* from scientific collections. Raw values of δ^2^H_f_ ranged from -107.3 to +5.0‰, with higher values at the Caatinga biome (northeast Brazil) and lower values at the Amazon and Pantanal. A Random Forest (RF) method was used to model the spatial surface, using a range of environmental data as auxiliary variables. The RF model indicated a negative relationship between δ^2^H_f_ and Mean Annual Precipitation, Precipitation in the Warmest Quarter, and Annual Temperature Range and positive relationships for amount-weighted February-April precipitation δ^2^H (δ^2^H_p(Feb-April)_) and Mean Annual Solar Radiation. Modelled δ^2^H_f_ values ranged from -85.7 to -13.6‰. Ours is the first δ^2^H_f_ isoscape for Brazil that can greatly assist our understanding of both ecological and biogeochemical processes controlling spatial variation in δ^2^H for this region. This isoscape can be used with caution, due to its poor predictive power (as found in other tropical regions) and can benefit from new sample input, new GNIP data, ecological and physiological studies, and keratin standard material better encompassing the range in feather samples from Brazil. So, we encourage new attempts to build more precise feather H isoscapes, as well as isoscapes based on other elements.

## Introduction

The world is rapidly losing biodiversity [1] due to human activities that include habitat loss, direct persecution and climate change [2, 3], an epoch defined as the Anthropocene. One factor of great importance is the increased illegal trade in wild species, especially birds [4]. Unfortunately, this activity is tremendously difficult to control and it is usually impossible to define the provenance of seized material. However, the development of isotopic techniques to infer origins of birds and other animals through the isotopic measurement of feathers or other tissues shows considerable promise as a forensic tool to assist with investigations of illegally traded birds and other wildlife [5]. Such tools are urgently needed in Brazil because this country holds huge biodiversity and deals with high illegal wildlife trade [6]. A first step in establishing such isotopic tools is the creation of predicted tissue-specific isotopic patterns or isoscapes to form the basis of provenance tracking.

Hydrogen and oxygen have well established isotopic patterns across continents [7] as demonstrated by δ^2^H and δ^18^O models from precipitation based primarily on the >60-year dataset from the International Atomic Energy Agency (IAEA), the Global Network of Isotopes in Precipitation (GNIP) [8]. Hydrogen in metabolically inactive tissues like feather keratin is fixed from drinking water and diet, with subsequent isotopic changes associated with metabolism. Feather δ^2^H is influenced, then, by diet composition, body size, and spatial environmental gradients in food web δ^2^H [9]. Because H in food webs is ultimately derived from environmental waters used by plants, the GNIP-based precipitation isoscapes provide a means of deriving tissue isoscapes of consumers providing appropriate linkages between the tissue of interest and environmental waters (reviewed in [5]). The power of such predictive tools will depend on how well environmental isoscapes are known for a particular region and how precisely tissue δ^2^H values can be linked to them.

Recent development of isotopic techniques to infer origins of birds and other animals have included more refined regional isoscapes [10–14] and used advanced statistical and artificial intelligence methodologies for isoscape building [15, 16]. Feather δ^2^H isoscapes are available today, ranging from continental models [17, 18] to country focused models [19, 20]. In addition, for birds, such isoscapes have been developed for specific foraging or migratory guilds [21]. For several animal taxa, tissue-specific isoscapes have been successfully built and used to determine the probabilistic origin of migratory species [5] such as monarch butterflies (*Danaus plexippus* [22]), dragonflies [23], and several birds [24, 25], including Neotropical migrants [21, 26–28].

Forensic applications based on stable isotope measurements are numerous [29–31]. In Brazil, isotopic forensic investigations have contributed to the understanding of the origin of seized marijuana [32, 33] and the patterns of human diet [34]. Sena-Souza et al. [35] presents an overview of isoscapes in the Brazilian context, exploring potential applications for understanding biogeochemical cycle mechanisms and processes in Brazil, as well as the potential application to solve crimes and track drug and illegal animal trade. However, few forensic studies have formally used large-scale isoscapes to predict animal origins.

Here, we established a feather δ^2^H isoscape for Brazil based on existing knowledge of precipitation-based δ^2^H models, environmental variables, and known-origin feathers from museum collections. Our goal was to derive a useful forensic tool that best fits the precipitation and feather isotope data based on a machine learning approach.

## Materials and methods

### Feather collection

Feathers were sampled from non-migratory passerine birds held in ten scientific ornithological collections in Brazil, and also from field campaigns between 2016–2018 in different Brazilian National Parks, as part of a Brazilian isotope forensic project (Edital CAPES 25/2014 –Pró-Forenses). Our dataset comprised 192 samples distributed among 129 locations in Brazil and included 49 species. We chose species belonging to six most representative sub-families of *Thraupidae* due to the wide range of distribution of this family in Brazil and its prominent use in the illegal bird trade [36]. We also included two specimens from family *Fringillidae* (genus *Euphonia*) and two from family *Cardinalidae* (genus *Cyanoloxia*). Taxonomic classification follows the Brazilian Ornithological Records Committee [37].

We prioritized specimens collected since 2013 and the use of flight feathers. Body and tail feathers were included only when flight feathers were not allowed from collections. Wing primaries or secondaries are more associated with a specific molting period, but, if grown simultaneously, body and flight feathers are expected to have similar δ^2^H_f_ values [38]. Due to the regional scarcity of samples collected within our target period, we also included a few feathers collected before 2013 (but after 2006). Sample information (species, year, locality, coordinates) is available as S1 Table.

Most birds in Brazil molt their remiges and rectrices following reproduction, showing little or no overlap between reproduction and molt, especially in Thraupidae and generally in frugivores [39, 40]. The reproductive season is mostly concentrated at the end of the dry season and the beginning of the rainy season; the molt is expected to occur in the middle to the end of the rainy season [40]. Molt duration seems to be longer in tropical regions, where it can take 100–230 days [41, 42], while lasting 42–105 days in temperate regions.

The procedures described here were approved by the Animal Use and Ethics Committee–University of Brasília (55712/2016). Field collection permits were done under the Chico Mendes Institute for Biodiversity Conservation’s license (SISBIO 8745–1). Feathers were transported from Brazil to Canada for stable isotopes analysis under CITES license (144180 and 144541). All Genetic Patrimony Access was also registered under the SISGEN system (#A4C6D05).

### Isotopic analysis of feathers

Feathers were steam-dried at 100°C for 20 min to meet requirements for international transportation to Canada. Before isotopic analysis, feathers were soaked in a 2:1 chloroform:methanol solution overnight, drained and air-dried under a fume hood. Distal feather vane was cut, weighed (~ 0.35 mg) into silver capsules, crushed, and placed into a Uni Prep carousel (Eurovector, Milan, Ital) at 60°C. The carousel was evacuated, flushed, and held under pressurized helium flow and samples were combusted in a Eurovector 3000 elemental analyzer using a glassy carbon reactor held at 1350°C. Resultant H_2_ gas was analyzed on a coupled Thermo Delta V Plus (Thermo, Bremen, Germany) continuous-flow isotope-ratio mass spectrometer at the University of Western Ontario LSIS-AFAR laboratory. Stable-hydrogen isotope results are reported for the non-exchangeable H fraction using the comparative equilibration method of Wassenaar and Hobson [43] based on within run (n = 5 each) measurements of CBS (-197‰) and KHS (-54.1‰) keratin standards. All results are reported in standard delta notation relative to the VSMOW (Vienna Standard Mean Ocean Water) standard scale. Based on within-run replicate measurements of standards, we estimate our measurement precision to be ~ ± 2‰.

### Exploratory analysis

Aiming to understand how sample characteristics affected our dataset, we ran exploratory analyses evaluating whether δ^2^H_f_ are affected by year of sample collection, feather type (body, tail or wing), biome where sample was collected, sub-family to which the species belong, latitude, and longitude (S1 Table).

The whole dataset was tested for normality and the variables year of collection, feather type, biome, and sub-family were independently tested through analysis of variance followed by a Tukey’s HSD (multiple comparisons of means). Latitude and longitude were tested through simple linear models. Additionally, year of collection was also tested through a linear model, where model residuals were also explored.

Subsequentially, we also ran a linear model selection (LM), with all variables, to explore variables’ importance to explain the measured δ^2^H_f_ values. We used “dredge” and “model average” functions to summarize the best models, ranking them by increasing Akaike’s Information Criteria (AICc) and considering models within ΔAIC < 2 as competitive. To run this analysis we used the package ‘*MuMIn*’ [44] available in software R [45].

The R code used and the results for each analysis are available as S1 File. Figures were edited using Inkscape free software [46].

### Isoscape modeling

Our Brazilian δ^2^H_f_ isoscape was modeled using a Random Forest analysis approach. First, using the “Recursive Feature Elimination” method, we selected environmental variables that most contributed to the prediction of δ^2^H_f_ values. Then, using the selected variables, we ran the Random Forest analysis to predict the best-fitting isoscape.

The R script used here was adapted from Bataille et al. [16] and Sena-Souza et al. [15]. Statistical analyses were performed in R version 4.1.3 [45] as well as RStudio interface [47]. Packages used were ‘*raster*’ [48], ‘*sf*’ [49], ‘*dismo*’ [50], ‘*caret*’ [51, 52], ‘*rgdal*’ [53], and ‘*randomForest*’ [54]. Plots and figures were built using package ‘*ggplot2*’ [55] and ‘*pdp*’ [56] in R, but also Inkscape [46] and Qgis [57] free software.

#### Environmental variables

We used data for temperature, precipitation, solar radiation, wind speed, water vapor pressure and altitude from WorldClim database [58]; and data for humidity and potential evapotranspiration, for the period of 1901–2019, was used from Climatic Research Unit (CRU) database [59].

The precipitation and the maximum and minimum temperature were calculated for the period of 2007–2018, and the bioclimatic variables for this period were calculated using package ‘*dismo*’[50]. The R script used here is available as S2 File. The mean annual solar radiation, wind speed and water vapor pressure were used for the period of 1970–2000, as this is the only period available in WorldClim database and recent tests confirm the reliability of this approach as recent proxies [60].

We also used data from Waterisotopes database [61], corresponding to isotopic composition of monthly amount-weighted precipitation (δ^2^H_p_) [62]. The correlation between δ^2^H_p_ and δ^2^H_f_ in Brazil (r^2^ = 0.167) was low when compared to the same correlation in North America (r^2^ = 0.83; [63]). In temperate regions, this correlation is usually higher when applied to the ‘growing season’ values, representing the isotopic precipitation values in the months when temperature is greater than 0º C. As temperatures in Brazil are rarely below 0º C, we investigated a timeframe that better represented H incorporation into food webs relevant to feather growth. For this reason, we selected the timeframe from February to April (amount-weighted February-April precipitation δ^2^H; δ^2^H_p(Feb-April)_), which gave us the best fit for δ^2^H_f_ observed data (r^2^ = 0.252). The idea of joining the more representative months is consistent with the idea of WorldClim bioclimatic variables, where year-quarters (3 subsequent months) are used as units of analysis. The criteria and R code used to define this timeframe is available as S4 Fig and S4 Table in S3 File. The R code used to extract the bioclimatic values for sample points, all the raster images, and the values for each variable are available as S4 File.

#### Variable selection

Before applying a Random Forest analysis, we used a Recursive Feature Elimination (RFE) method to select a reduced number of variables most relevant in predicting our δ^2^H_f_ values from our known-origin feathers. This approach reduces the collinearity between variables which would result in overfitting [64]. Here, we used function “rfFuncs”, method “cv”, and 10-fold cross-validation as outer sampling method. We started with a subset including all possible variables. The best combination of variables was considered for the model with the lowest Root Mean Squared Error (RMSE) after cross-validation.

#### Random Forest

Random Forest is a machine-learning model algorithm based on decision trees that are merged into a single prediction, reducing noise and increasing accuracy [65]. Here, the RF model was run using a training subset corresponding to 80% of samples for the ensemble learning and a holdout subset corresponding to 20% to test the model [51]. The model randomly selects a set of variables to be used in each tree split or node, and this parameter (m_try_) was defined as m_try_ = 3, based on out-of-bag error and avoiding m_try_ = 1 due to overfitting (see S4 File for details). The importance of each covariate to the final model was assessed by the percentage increase in the Mean Standard Error (%incMSE), indicating how much of the model accuracy is lost when that variable is removed. Each variable’s influence on the δ^2^H_f_ was assessed using partial dependence plots (package ‘*pdp’* [56] and ‘*ggplot2’* [55]).

#### Model validation

Model performance was evaluated through linear regressions between observed δ^2^H_f_ values and predicted δ^2^H_f_ values, for the following datasets: (A) training dataset after 10-fold cross-validation and (B) testing dataset (20% of data). For each linear regression we compared the Mean Absolute Error (MAE = mean of model residuals^2^), the Root Mean Squared Error (RMSE = the root squared of MAE), and the r-squared (r^2^).

#### Spatial prediction

Spatial predictions were conducted using the function “predict” available in the ‘*raster*’ package [48]. The final isoscape was obtained from a mean of the spatial predictions from 20 RF models using the same 80% dataset previously defined. The R code and raw results are available as S4 File.

### Results

**Fig 1:**
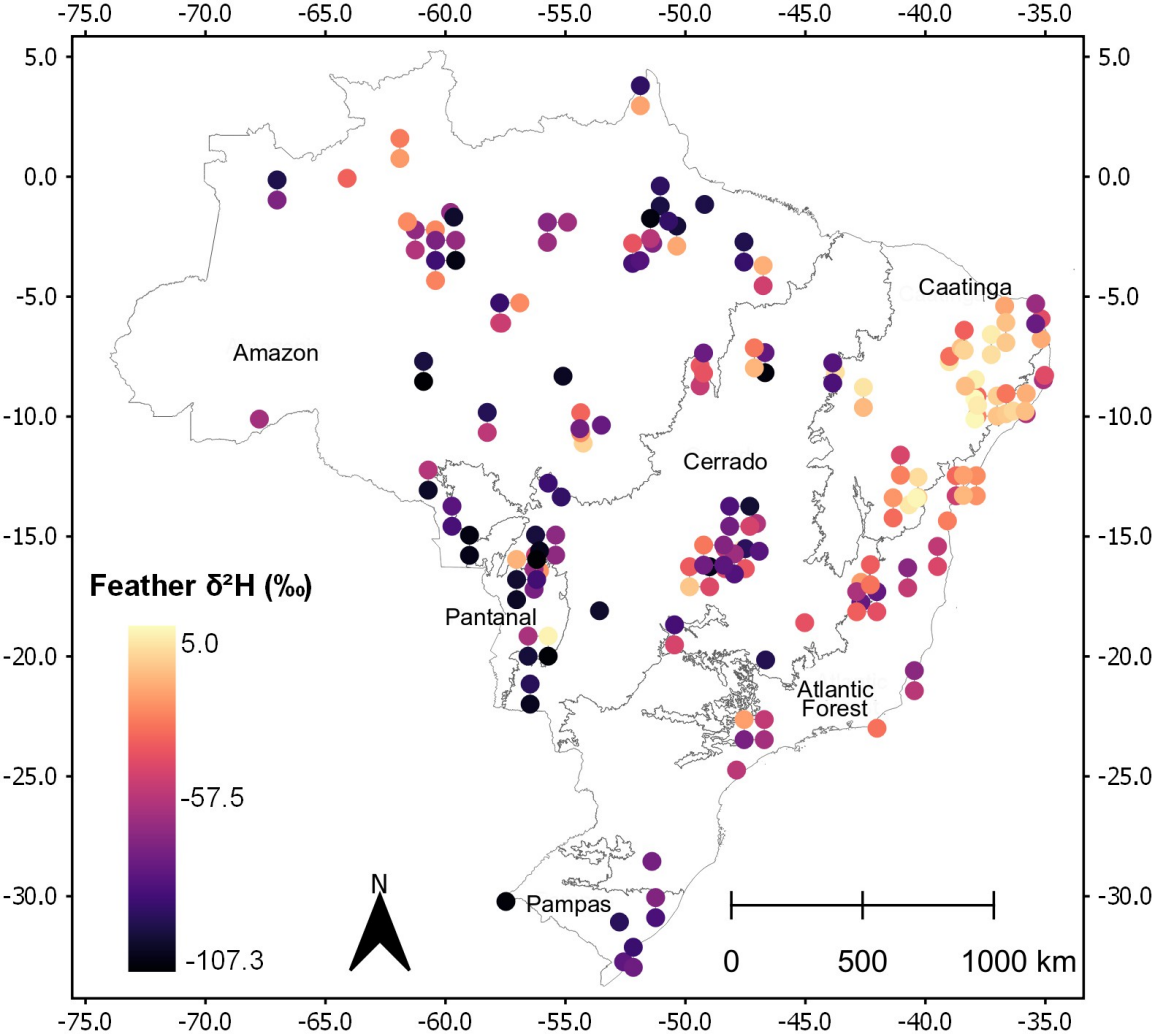
Raw observed δ^2^H_f_ values (‰ VSMOW). Biome limits and names are indicated (Biome limits from Instituto Brasileiro de Geografia e Estatística–IBGE—https://www.ibge.gov.br/geociencias/downloads-geociencias.html).

Values of δ^2^H_f_ ranged from -107.3‰ to +5.0‰ (N = 192; Fig 1). The higher values were found in northeast region (Caatinga biome) and the lower values were found in the northwest region (Amazon and Pantanal biomes).

### Exploratory analysis

**Fig 2:**
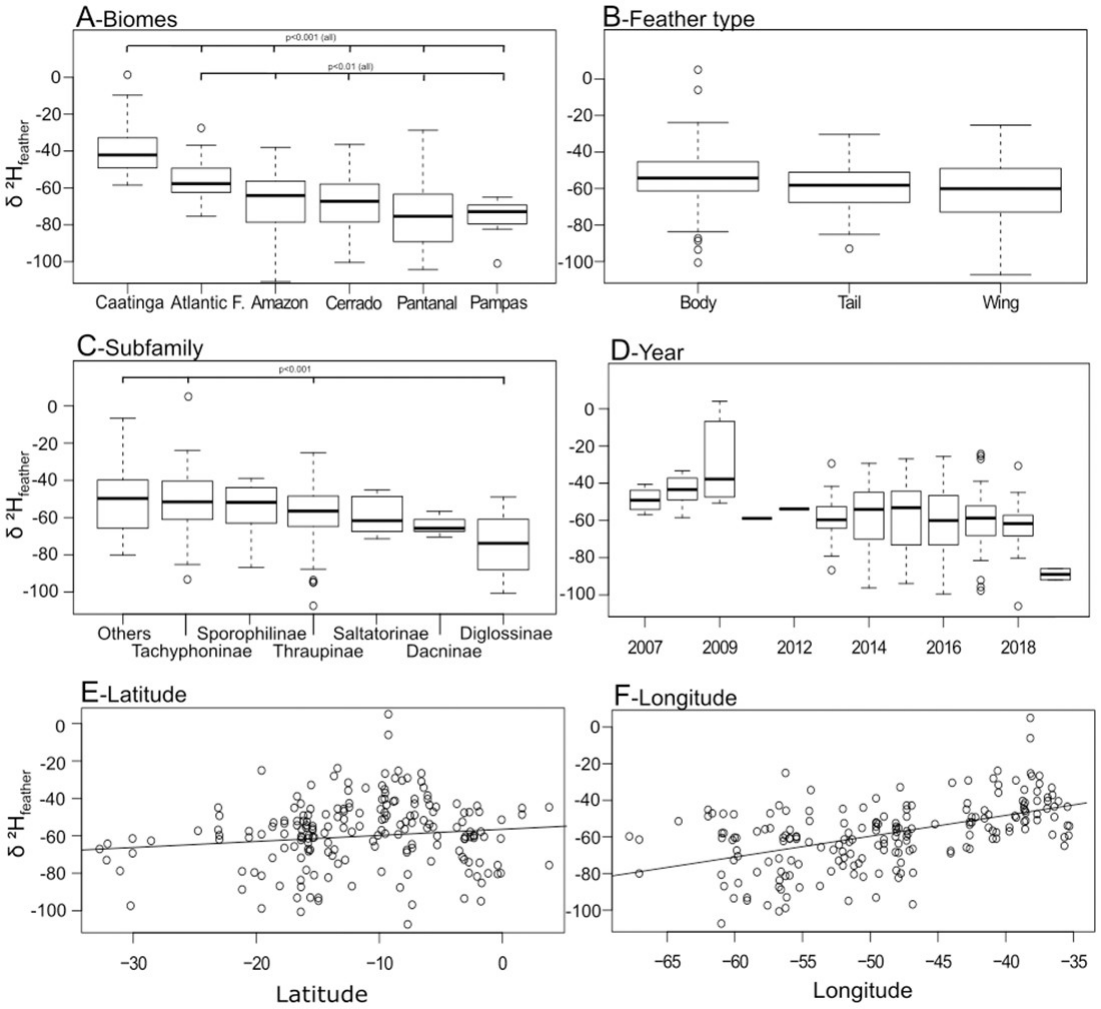
Effect of variables on the raw observed δ^2^H_f_ values (‰ VSMOW). (A) Biomes where the sample was collected; (B) Feather type; (C) Bird’s subfamilies; (D) Year when sample was collected; (E) Longitude; and (F) Latitude. The p values refer to individual analyses of variance followed by Tukey’s HSD or to linear models (latitude and longitude).

Using individual exploration of each variable through analysis of variance and linear models, the Caatinga biome had higher δ^2^H_f_ values than all other biomes (Tukey’s HSD: p < 0.001 for all comparisons), followed by Atlantic Forest values that were also higher than values found in all other biomes (Tukey’s HSD: p < 0.01 for all comparisons) (Fig 2A and S1 File). Samples from Amazon, Cerrado, Pantanal and Pampas had lower values, but they were not significantly different from each other. Feather types presented no significant differences for δ^2^H_f_ values (Fig 2B and S1 File), although such differences in values for wing and body feathers were close to significance (Tukey’s HSD: p = 0.052). Overall, most subfamilies did not differ in δ^2^H_f_ (Fig 2C and S1 File), but subfamily *Diglossinae* had lower δ^2^H_f_ values than the subset including ‘other’ less represented subfamilies, and the subfamilies *Tachyphoninae* and *Thraupinae* (Tukey’s HSD: p < 0.001 for all comparisons). The most sampled years (2013–2018) were homogeneous in δ^2^H_f_ but 2009 had higher values than years 2013–2019 (Fig 2D and S1 File). The years 2007–2012 represented only 17 samples (11% of total samples). Due to this difference, variable year was further explored with an additional linear model and exploration of residuals. The distribution of residuals was normal (Shapiro Test: W = 0.99, p = 0.20) and residuals from year 2009 were not out of the residual range. For this reason, samples from 2009 were kept in the following isoscape modeling. Latitude and longitude were positively related to δ^2^H_f_ values, but only longitude was significantly related to δ^2^H_f_ values (Tukey’s HSD: p < 0.001; Fig 2E and 2F and S1 File), with coastal longitudes corresponding to higher values of δ^2^H_f_.

According to our exploratory model selection, all included covariables were important to explain our δ^2^H_f_ values. Variable biome, subfamily, and longitude, were included in all models with ΔAIC < 2 (Table 1). After an average of models with ΔAIC < 2, the final conditional average model indicates biome Caatinga (p = 0.004), feather from wing (p = 0.03), and longitude (p = 0.003) as significantly influencing our δ^2^H_f_ values (S1 File).

**Table 1:** Exploratory model selection results for observed δ^2^H_f_ values. Showing the more competitive models and their degrees of freedom, AICc, ∆AIC and weight.

LM full model: δ^2^H_f_ ~ Year + Feather type + Longitude + Latitude + Biome + Subfamily				
Selected models	df	AICc	∆AIC	Weight
Biome + Long + Subfamily + Feather type	16	1561.6	0.00	0.230
Biome + Long + Subfamily + Feather type + Lat	17	1562.3	0.63	0.168
Biome + Long + Subfamily	14	1562.5	0.85	0.150
Biome + Long + Subfamily + Year	15	1563.1	1.52	0.108
Biome + Long + Subfamily + Feather type + Year	17	1563.5	1.91	0.088

Only models with ∆AIC < 2 were considered competitive.

### Isoscape modeling

#### Recursive feature elimination

Through the RFE method, we selected the environmental variables predicting the δ^2^H_f_ values from our known-origin feathers. The model presenting the lowest RMSE after cross-validation included the following five environmental variables: Annual Temperature Range (Bio7), Mean Annual Precipitation (Bio12), Precipitation at the Warmest Quarter (Bio18), Mean Annual Solar Radiation, and δ^2^H_p(Feb-April)_.

#### Random Forest

Mean Annual Precipitation was the most important predictor for our known-origin feather δ^2^H_f_ values, and its removal from the model caused an ~20% increase in the Mean Standard Error (MSE). The δ^2^H_p(Feb-April)_ was the second most-important variable (~15%), followed by Precipitation at the Warmest Quarter (~13%), Mean Annual Solar Radiation (~7%), and Annual Temperature Range (~5%).

**Fig 3:**
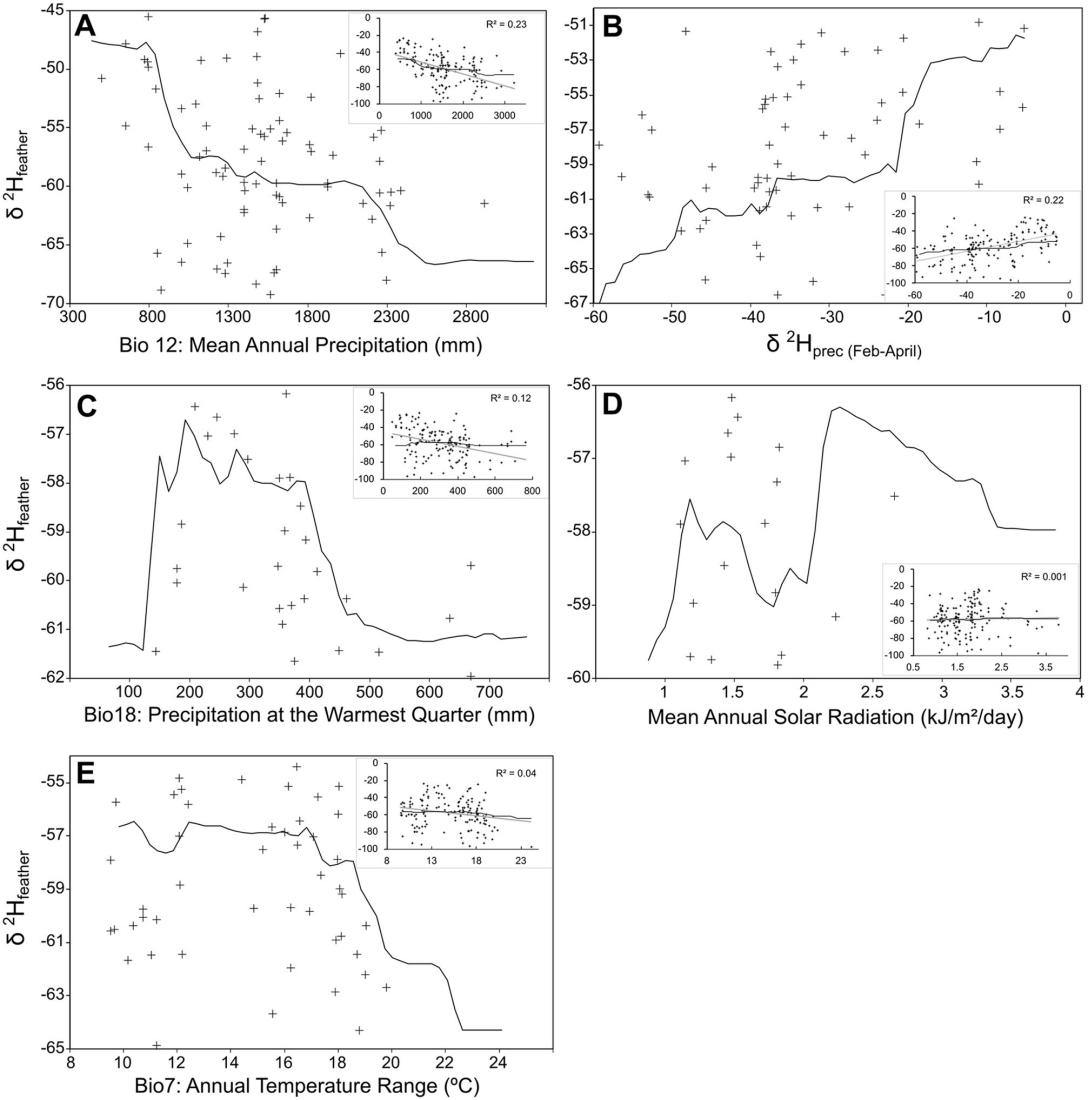
Influence of selected variables to feather δ^2^H prediction. Partial plots showing the influence of the five selected variables to δ^2^H_f_ (‰ VSMOW) prediction using the Random Forest algorithm and each associated linear regression, with corresponding adjusted r^2^ values (upper smaller plot). (A) Mean Annual Precipitation; (B) δ^2^H_p_ mean values from Feb-April; (C) Precipitation at the Warmest Quarter; (D) Mean Annual Solar Radiation; (E) Annual Temperature Range.

Each variable had a different influence on δ^2^H_f_ values. We observed each of these relationships through the partial plots (Fig 3). Mean Annual Precipitation (MAP) varied from 383–3221 mm and most of our samples were concentrated in areas holding 1000–2000 mm of rain (annual mean). MAP was negatively related to δ^2^H_f_ (Fig 3A), as sites with MAP lower than 1000 mm had higher δ^2^H_f_ values while sites with MAP higher than 2000 mm had lower values. The isotopic precipitation values (δ^2^H_p(Feb-April)_) varied from -5.2 to -59.4‰ and our samples were more evenly distributed for this environmental variable. The δ^2^H_p(Feb-April)_ was positively related to the observed δ^2^H_f_ values (Fig 3B).

The Precipitation at the Warmest Quarter (PWQ) varied from 50.8–762.1 mm and our samples were concentrated in areas with 200–450 mm of rain at the warmest period of the year. The PWQ was negatively related to δ^2^H_f_ (Fig 3C), as sites with higher PWQ had lower δ^2^H_f_ values. The Mean Annual Solar Radiation (MASR) varied from 0.81–3.78 kJ/m^2^/day and our samples were concentrated in areas with 1–2 kJ/m^2^/day. The MARS was positively related to δ^2^H_f_ (Fig 3D), as sites with higher solar radiation incidence had higher δ^2^H_f_ values. Finally, the Annual Temperature Range (ATR) varied from 8–24 ºC and was negatively related to δ^2^H_f_ values (Fig 3E).

#### Model validation

**Fig 4:**
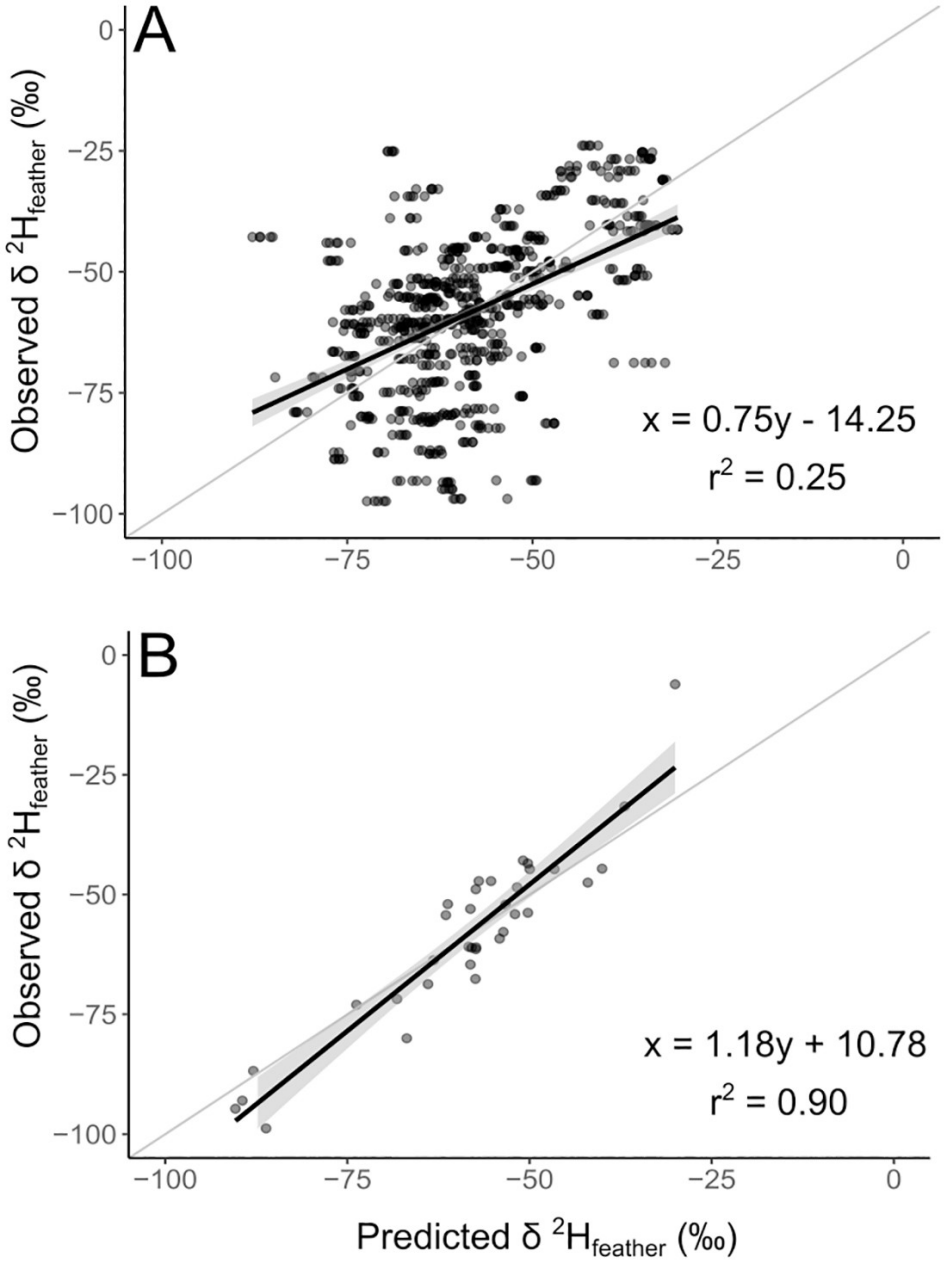
Observed *vs*. predicted δ^2^H_f_ values. Scatter plots of observed *vs*. predicted δ^2^H_f_ (‰ VSMOW) values. (A) training dataset after 10-fold cross-validation (MAE = 231.21, RMSE = 15.21); and (B) holdout testing dataset (MAE = 34.73, RMSE = 5.89). Regression equation and r^2^ also provided. The gray line corresponds to slope 1 and intercept 0.

After 10-fold cross-validation, the predicted δ^2^H_f_ values explained 25.6% (F_1,778_ = 270.1; p < 0.001) of the variance in observed values, with a MAE of 231.21 and a RMSE of 15.21 (Fig 4A). For model-predicted δ^2^H_f_ and the holdout testing data (n = 36), the predicted values explained 90.5% (F_1,34_ = 337,3; p < 0.001) of the variation, with a MAE of 34.73 and a RMSE of 5.89 (Fig 4B). For both validations, slope was significantly different from 1 and intercept was different from 0 (p < 0.01; See S4 File for more information).

#### Spatial prediction

**Fig 5:**
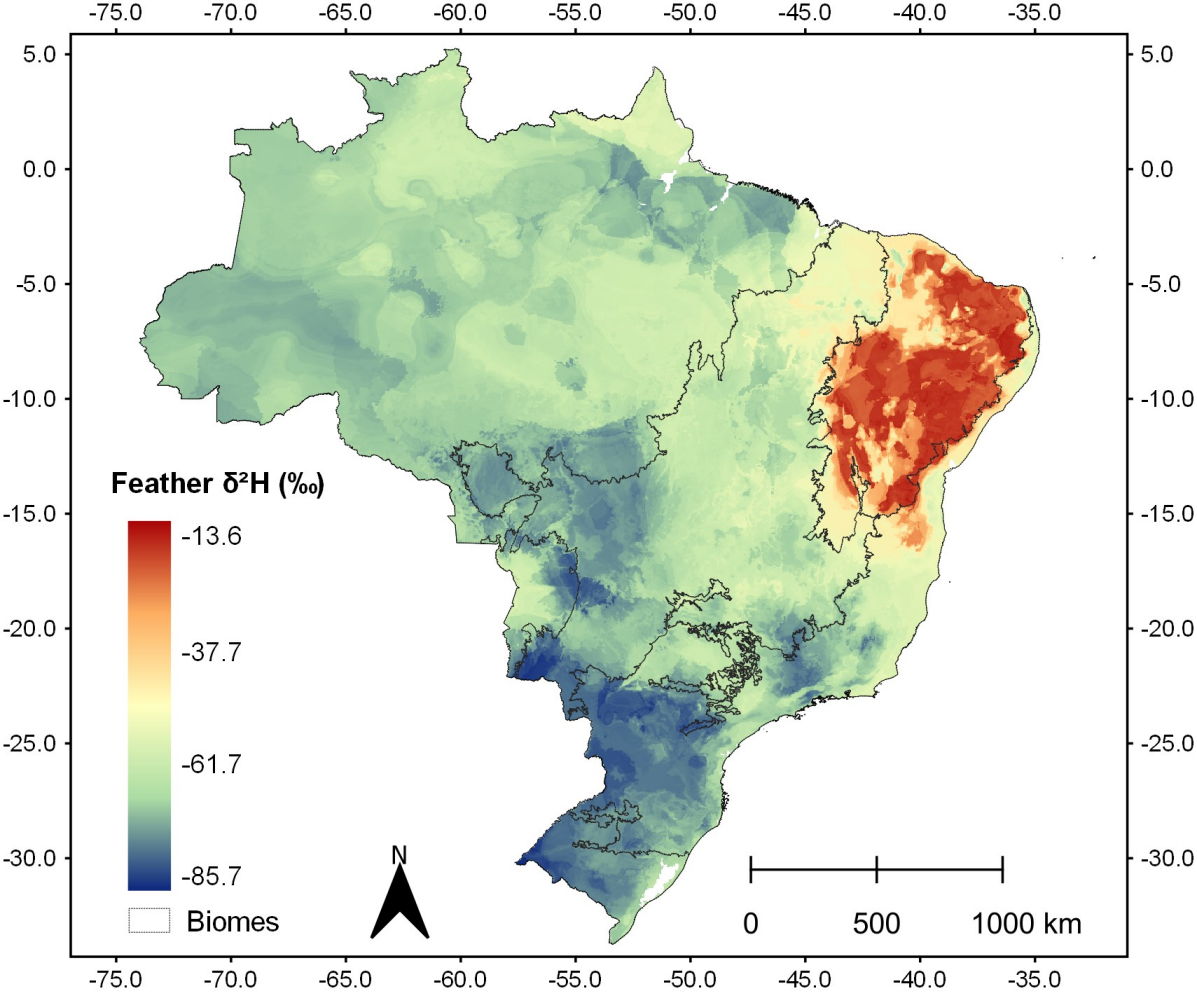
Isoscape. Final spatial prediction of δ^2^H_f_ (‰ VSMOW) for Brazil after 20 Random Forest models, based on Mean Annual Precipitation, Precipitation at the Warmest Quarter, Mean Annual Solar Radiation, δ^2^H_p_ from Feb-April, and Annual Temperature Range. Biome limits from Instituto Brasileiro de Geografia e Estatística–IBGE; Spatial variables from WorldClim database.

After running the 20 RF models, the final mean spatial prediction (isoscape) represented a δ^2^H_f_ range between -85.7 and -13.6‰ (Fig 5).

## Discussion

Here we developed the first feather hydrogen isoscape for Brazil based on an extensive analysis of available climatic data using a machine learning approach. Besides its novelty, this is the first such attempt in the Brazilian territory and we acknowledge the need for further improvements. The isoscape presented here has a low predictive power when compared to isoscapes developed for temperate regions. Possible reasons for such low predictability are (1) the poor modelled precipitation isoscape for Brazil due to low GNIP station coverage, (2) the complex climate and hydrology of Brazil, (3) the relatively low sample sizes of birds when compared to other works in temperate zones, (4) the complexities related to species ecology, migration and molt strategies, and (5) the poor ability to evaluate over which period of integration of H from precipitation supports local food webs.

The GNIP coverage in Brazil accounted for 15 to 20 stations between the 1980’s and 1990’s. However, those stations were deactivated and only one or two stations operated between 2008 and 2013 [66, 67]. Since 2018, an international initiative and national task force has installed 10 stations and plans to install 12 more [68]. This initiative will certainly provide a more reliable database for future studies. It is important to mention that even with these 22 stations in operation, our dataset still comprises an important number of independent locations (129), adding important isotopic information to the Brazilian context.

Brazil is a continental country, holding a land surface of 8.5 million km^2^ and with a complex climatic and hydrological structure. The main air masses influencing the climate are the Equatorial Atlantic, the Tropical Atlantic, and the Polar Atlantic air masses [69]. All of this complexity brings a high spatial and seasonal variability to the isotopic composition of meteoric precipitation [66]. Although complex, the effect of isotopic variability in precipitation might be reduced if biomes or geographical regions are analyzed separately (ex. [19, 70]).

When avian sample size is compared to studies conducted in North America (n = 461 samples in [19]; and n = 544 for calibration and n = 269 for validation in [18]), our sample size is relatively low and this might be responsible for part of the reduced predictability. Main reasons for this low sample size are the poor availability of collected specimens over the country for recent years and the low willingness of curators from some Brazilian scientific collections to provide samples. Scientific collections (especially museums) hold a very important set of taxonomical information, but these collected birds could serve additional uses as we have demonstrated through the provision of a small feather sample per individual. In this way, Brazilian science urges curators of scientific collections to be more flexible in making materials available to the scientific community (see Avisample.net [71]). Another observed caveat that can also be considered is support for the establishment of long-term avian banding stations in Brazil, in order to both provide feather samples and more information about migratory patterns. Nevertheless, we also support any fieldwork involving bird capture that could include feather collection in its activities (under the appropriated licenses). If these feathers are well identified (species name, precise coordinates) and deposited in scientific collections, they can be a great source of information for future isoscape development.

Although ornithological knowledge has grown in Brazil in the last decades, there is still a huge lack of basic ecological information for several species, including information related to molt strategies that are important here. This caveat also reflects on the state of physiological knowledge, resulting in a poor ability to evaluate over which period H from precipitation is integrated into local food webs. That is, it is currently unclear how birds integrate water into feather in tropical environments and in the different biomes found in Brazil. This knowledge can be established experimentally or further investigated by the collection and analysis of feathers close to the new GNIP stations in Brazil, providing new insights to unravel this relationship.

Another important caveat is the absence of a keratin standard material for higher δ^2^H values. Here we used the two recognized standards (CBS: -197.00 ± 0.9‰ and KHS: -54.1 ± 1.1‰; [72]) for keratinous tissues, but our sample values went beyond the available more positive standard values, raising the need for the development of a new standard to be used on future researches aiming to explore areas with less negative isotopic values. Our highest values came from the Northeast region, where several domestic animals, such as goats and donkeys, have keratinized tissue, and could be good candidates to provide reference material. Nonetheless, since the calibration relationship is expected to be linear over a broad range, we assume this effect was minor.

### Exploratory analysis

While previous global models strongly account for latitudinal variation, the geospatial structure of δ^2^H_f_ modelled for Brazil varied longitudinally, with values decreasing from east to west resulting in feathers enriched in ^2^H on the northeastern coast and more depleted westward. This reflects the atmospheric circulation over Brazil that predominantly brings rainfall from the east coast to the interior [69].

Feather type, avian subfamily, and year of collection introduced variability to our δ^2^H_f_ dataset. Wing feathers (i.e. primaries, secondaries) are preferred over body and tail feathers as they are less likely to be lost and replaced over periods that do not represent the annual molt. As expected, body feathers presented higher variability than tail and wing feathers. The most distinguishable subfamily in our sample was *Diglossinae*, which comprises a highly trafficked species (*Sicalis flaveola*). The observed differences among years were not taken here as a concern, since they can be associated with the region from which those samples came from. The 2008, 2011 and 2012 samples were from Atlantic Forest, while the 2009 samples were from Caatinga (with expected higher δ^2^H_f_ values). As Caatinga is represented by additional 23 samples, the higher δ^2^H_f_ values were likely not associated with a specific year, but to the region of origin.

The isotopic variability resulting from different subfamilies and feather types should be considered. We recommend that future researches should focus initially on one species or genera and on wing feathers, to reduce the possible associated noise.

### Biomes, bioclimatic variables, and air masses

The dry northeastern region (Caatinga) presented the highest δ^2^H_f_ values and part of this effect can be explained by the proximity to the coast. This region has the lowest values of Mean Annual Precipitation (MAP; <1,000 mm) and high Mean Annual Solar Radiation (MASR; 1.8–1.9 kJ/m^2^/day). It receives the Equatorial Atlantic air mass, which is mostly hot and humid [69], but the region also presents orographic barriers that prevent rainfall from the Atlantic Ocean entering the region, contributing to the overall aridity. The high values found are consistent with patterns found for migratory Barn Swallows (*Hirundo rustica*), where breeding individuals from Mississippi presented mean δ^2^H_f_ equal to -36.4‰ and were associated to non-breeding grounds in northeast South-America [26]. This contrast may be particularly useful in animal trafficking applications, as the region is usually a considerable source of illegally captured animals [73].

The northwestern region (Amazon) presented a 20‰ range in δ^2^H_f_ values with an isotopic gradient observed from east to west. The region presents the highest MAP (1500–3500 mm) and a low Annual Temperature Range (ATR; 10º C in the north and 15º C in the south to the east). This region is affected by the Equatorial Continental air mass, which is hot and humid, and transports humidity to the central part of the country [66, 69]. Isotopic homogeneity was previously observed in modeled precipitation isoscapes and have been reported as an effect of the strong and relatively uniform evapotranspiration from the Amazon forest [28, 74]. Terzer-Wassmuth et al. [70] also observed a significant influence of outgoing longwave radiation on Amazonian basin precipitation δ^2^H values. However, our findings suggest that this region may not be so homogeneous, but due to the lack of sampling on these remote landings, we suggest that more effort should be dedicated to isotopic investigations in this region. However, our findings suggest the possibility of using a more structured isoscape based on tissue δ^2^H as a geographical tracer of animals from the region.

The central region (Cerrado) is primarily represented by savanna. It is formed by a very heterogeneous formation, where grasslands and gallery forests occur in juxtaposition. This heterogeneity was not reflected in our δ^2^H_f_ values, but we did find a depletion in ^2^H_f_ from northeast to southwest. This pattern may occur due to the atmospheric circulation of the rains in the central region of Brazil, being more affected by Equatorial air masses in the northern part, while the southern part is more affected by Tropical (drier) air masses [69]. Additionally, precipitation coming from the Amazon influences the water circulation towards the southeast. As a result, the δ^2^H values of precipitation in the west of the Cerrado are more depleted in ^2^H.

The central western region (Pantanal) is located west of the Cerrado biome. It is a seasonally flooded steppe savanna, affected by the Equatorial Continental air mass coming from the Amazon and by the Polar Atlantic air mass [69]. The MAP is similar to Cerrado (1,000–1,500 mm), however, the region receives lower MASR (1.5–1.6 kJ/m^2^/day) than northern Cerrado and Caatinga. This region is more depleted in ^2^H in its eastern portion, similar to the adjacent Cerrado portion. However, the western portion presented a similar pattern to the δ^2^H_p(Feb-April)_ surface, with higher values than the eastern portion.

The Atlantic Forest is present along the Brazilian coast extending to the interior of the country in the south. This region had more latitudinal variation in feather δ^2^H, with higher values in the northern portion and lower in the southern. The pattern aligns with the MAP values, the δ^2^H_p(Feb-April)_ surface, the PWQ and the MARS values, with southern portion holding higher precipitation amounts, lower δ^2^H_p_ values and lower solar radiation. This biome is affected by the Tropical Atlantic air mass in the coast and by the Polar Atlantic mass in the south [69]. The presence of a polar air mass, associated with higher altitudes can be responsible for the higher ^2^H depletion in the region.

The southernmost region (Pampas) is a biome dominated by grasslands. It has the highest ATR (20 ºC) and intermediate values for the other bioclimatic variables. The region is affected by the Polar Atlantic air mass [69] and presented low δ^2^H_f_ values consistent with the most southern and coldest region in Brazil.

### Model validation and uncertainty

Our model presented a spatial pattern consistent with the expected result for Brazil. However, users should pay attention to some weaknesses of the model regarding the spatial pattern of the samples and the model’s performance in explaining the variance of observed data. We are aware that the testing dataset should be spatially independent of the training dataset [75]. The high performance from the holdout testing data (R^2^ = 0.9) may indicate a strong spatial dependency between the testing and training dataset. That is expected because our data comes from museums and is highly related to bird capture hotspots. To minimize this limitation, we also used the 10-fold cross-validation with five repetitions to access the model performance. We believe it is the most reliable and conservative method to assess the model performance.

As shown by model validation using the training dataset after 10-fold cross-validation, our model explained 25.6% of δ^2^H_f_ variation, significantly departing from the 1:1 correspondence line. As shown in Methods, the initial relationship between δ^2^H_p_ from waterisotope database and observed δ^2^H_f_ in Brazil explained 16.7% of the variation in δ^2^H_f_, while the relationship found for waterisotope database selected quarter (February to April) explained 25.2% of the variation (S4 File). This result indicates that our model presented a very low improvement when compared to a simple linear regression using only the δ^2^H_p(Feb-April)_ variable, and that improvements on GNIP network in Brazil can likely bring great benefits for the development of future tissue-specific hydrogen isoscapes.

The isoscape presented here can help direct future sampling for new isoscapes and for origin assignments, based on designed sampling strategies. According to Contina et al. (2021) [76], an end-point strategy might help to reduce sampling effort and the uncertainties due to model extrapolation. This strategy is based on training data from the maximum and minimum latitudinal or isotopic values. Nevertheless, those authors indicate that the best strategy can be variable according to the research questions and the study constrains, and should be carefully evaluated.

### Concluding remarks

The isoscape developed and presented here was designed to test the possibility of using isotopic tracing techniques to assist in academic and enforcement applications in Brazil. Although presenting a lower predictability when compared to isoscapes from Northern regions, it is aligned with low predictability found in other tropical regions [77]. The observed low variation (-85.7 to -13.6‰) for δ^2^H_f_ values over large regions of Brazil can be compensated for through the use of informed Bayesian priors [78, 79] in assignments as well as the use of stable isotopes of other elements (as δ^13^C, δ^15^N, δ^18^O, δ^87^Sr). A multiple-isotopic approach has been successfully used in other regions, improving the origin-assignment of migratory bird species [17, 24, 26, 28, 80, 81] and should also be developed for Brazil and neighboring regions.

We encourage the improvement of this isoscape through the use of a larger sample size focused on a taxon-specific approach and on flight feathers, the use of new keratin standard material for higher δ^2^H_f_ values, and the use of a more robust Brazilian surface of water δ^2^H.

Despite the acknowledged improvements needed and weaknesses discussed, the provided isoscape can be very useful for broader scale questions, such as identifying whether an animal seized in Brazil comes from captivity or from the wild. Other uses are also viable if the isoscape is used with caution and attention to its limited spatial precision.

## Supporting information

S1 Table Samples’ information. Identification number (ID), year of collection, species name under CBRO (Brazilian Ornithological Records Committee [37]), family, subfamily, feather type (body, wing or tail), latitude and longitude, Brazilian state and biome where feathers were collected, corresponding university’s scientific collection (museum*) where feathers were deposited, and feather’s hydrogen isotopic (‰ VSMOW) raw values.
(PDF)

S1 File Exploratory analysis. R code and results for Analysis of Variance followed by a Tukey’s HSD (variable year of collection, feather type, biome, and sub-family), Linear Models (variable latitude and longitude), and Model Selection with all variables.
(PDF)

S2 File Bioclimatic variables calculation for a specific timeframe. R code to calculate WorldClim bioclimatic variables for a specific timeframe.
(PDF)

S3 File Defining a better timeframe for Isotopic precipitation values in Brazil. R code and results. Including S4 Fig (Linear regressions between δ^2^H_f_ and δ^2^H_p_) and S4 Table (Precipitation isotopic values extracted from waterisotopes.org).
(PDF)

S4 File Modeling the Brazilian hydrogen isoscape. First step- Prepare data. R code to input raster and observations, and extract values. Including S5 Table in S4 File with extracted values; Second step- Selection of variables using Recursive Feature Elimination–RFE. R code to run RFE and plot results; Third step- Prepare data and run Random Forest (RF). R code to run RF and explore results with partial plots; Fourth step- Model validation. R code to validate the model and plot results; Fifth step- Spatial prediction. R code to apply modeled values into an isoscape, calculate method uncertainty, mean, standard deviation and coefficient of variation.
(PDF)
