# Supplementary material for: A feather hydrogen (δ2H) isoscape for Brazil
Source: PLoS One. 2022 Aug 3;17(8):e0271573. doi: 10.1371/journal.pone.0271573 (PMC9348672; doi:10.1371/journal.pone.0271573)
Supplement: S1 Table — Identification number (ID), year of collection, species name under CBRO (Brazilian Ornithological Records Committee [37]), family, subfamily, feather type (body, wing or tail), latitude and longitude, Brazilian state and biome where feathers were collected, corresponding university’s scientific collection (museum*) where feathers were deposited, and feather’s hydrogen isotopic (‰ VSMOW) raw values. (PDF) [file pone.0271573.s001.pdf]

S1 Table. Samples' information.

Identification number (ID), year of collection, species name under CBRO (Brazilian Ornithological Records Committee [1]), family, subfamily, feather type (body, wing or tail), latitude and longitude, Brazilian state and biome where feathers were collected, corresponding university's scientific collection (museum*) where feathers were deposited, and feather's hydrogen isotopic (‰ VSMOW) raw values.

ID	Year	Species	Family	Subfamily	Feather type	Lat	Long	State	Biome	Museum	δ^2H_f (‰)
RDA001	2016	*Tangara cayana*	Thraupidae	Thraupinae	Body	-11.605	-41.041	BA	Caatinga	UFBA	-54.9
RDA002	2016	*Tangara cayana*	Thraupidae	Thraupinae	Body	-11.605	-41.041	BA	Caatinga	UFBA	-47.9
RDA003	2018	*Dacnis cayana*	Thraupidae	Dacninae	Body	-16.302	-40.738	MG	Atlantic Forest	UFBA	-61.0
RDA004	2018	*Dacnis cayana*	Thraupidae	Dacninae	Body	-16.302	-40.738	MG	Atlantic Forest	UFBA	-56.7
RDA005	2018	*Coryphospingus pileatus*	Thraupidae	Tachyphoninae	Body	-16.910	-42.689	MG	Cerrado	UFBA	-45.1
RDA006	2018	*Dacnis cayana*	Thraupidae	Dacninae	Body	-16.910	-42.689	MG	Cerrado	UFBA	-65.7
RDA007	2017	*Tangara cayana*	Thraupidae	Thraupinae	Body	-13.386	-41.345	BA	Caatinga	UFBA	-45.5
RDA008	2017	*Tangara cyanoventris*	Thraupidae	Thraupinae	Body	-13.386	-41.345	BA	Caatinga	UFBA	-49.9
RDA009	2015	*Coryphospingus pileatus*	Thraupidae	Tachyphoninae	Body	-13.681	-40.686	BA	Caatinga	UFBA	-28.1
RDA010	2017	*Trichothraupis melanops*	Thraupidae	Tachyphoninae	Body	-13.397	-40.364	BA	Atlantic Forest	UFBA	-23.9
RDA011	2019	*Sporophila maximiliani*	Thraupidae	Sporophilinae	Wing	-18.095	-53.580	MS	Cerrado	UFMT	-86.8
RDA012	2019	*Sicalis flaveola*	Thraupidae	Diglossinae	Tail	-15.605	-56.054	MT	Cerrado	UFMT	-93.0
RDA013	2013	*Tangara sayaca*	Thraupidae	Thraupinae	Wing	-16.367	-56.299	MT	Pantanal	UFMT	-62.2
RDA014	2013	*Saltator coerulescens*	Thraupidae	Saltatorinae	Wing	-3.251	-51.784	PA	Amazon	UFMT	-71.4
RDA015	2013	*Ramphocelus carbo*	Thraupidae	Tachyphoninae	Wing	-3.251	-51.784	PA	Amazon	UFMT	-52.5
RDA016	2014	*Dacnis cayana*	Thraupidae	Dacninae	Wing	-13.651	-59.769	MT	Amazon	UFMT	-67.4
RDA017	2014	*Tangara palmarum*	Thraupidae	Thraupinae	Wing	-12.225	-60.711	RO	Amazon	UFMT	-86.9
RDA018	2016	*Sporophila angolensis*	Thraupidae	Sporophilinae	Wing	-3.500	-51.894	PA	Amazon	UFMT	-68.0
RDA019	2014	*Dacnis cayana*	Thraupidae	Dacninae	Wing	-13.816	-59.687	MT	Cerrado	UFMT	-70.6
RDA020	2014	*Schistochlamys melanopis*	Thraupidae	Thraupinae	Wing	-14.946	-58.999	MT	Cerrado	UFMT	-94.7
RDA021	2014	*Sporophila angolensis*	Thraupidae	Sporophilinae	Wing	-12.233	-60.728	RO	Amazon	UFMT	-57.9
RDA022	2015	*Tangara sayaca*	Thraupidae	Thraupinae	Wing	-15.589	-56.106	MT	Cerrado	UFMT	-45.6
RDA023	2015	*Tangara cyanicollis*	Thraupidae	Thraupinae	Wing	-13.354	-55.176	MT	Cerrado	UFMT	-74.9
RDA024	2014	*Tachyphonus rufus*	Thraupidae	Tachyphoninae	Wing	-14.946	-58.999	MT	Cerrado	UFMT	-93.2
RDA025	2017	*Ramphocelus carbo*	Thraupidae	Tachyphoninae	Wing	-3.078	-51.776	PA	Amazon	UFMT	-61.4
RDA026	2017	*Tangara mexicana*	Thraupidae	Thraupinae	Wing	-9.824	-58.259	MT	Amazon	UFMT	-79.2
RDA027	2017	*Tangara mexicana*	Thraupidae	Thraupinae	Wing	-9.824	-58.259	MT	Amazon	UFMT	-57.0
RDA028	2017	*Paroaria capitata*	Thraupidae	Thraupinae	Wing	-16.355	-56.317	MT	Pantanal	UFMT	-60.4

ID	Year	Species	Family	Subfamily	Feather type	Lat	Long	State	Biome	Museum	δ²Hf (‰)
RDA029	2007	*Tachyphonus rufus*	Thraupidae	Tachyphoninae	Body	-9.774	-35.846	AL	Atlantic Forest	UFAL	-40.5
RDA030	2007	*Tangara desmaresti*	Thraupidae	Thraupinae	Body	-24.737	-47.868	SP	Atlantic Forest	UFAL	-57.3
RDA031	2008	*Euphonia violacea*	Fringilidae	Others	Body	-9.399	-36.145	AL	Atlantic Forest	UFAL	-49.1
RDA032	2008	*Nemosia pileata*	Thraupidae	Others	Body	-9.523	-36.290	AL	Atlantic Forest	UFAL	-37.1
RDA033	2008	*Lanio cristatus*	Thraupidae	Tachyphoninae	Body	-9.768	-36.341	AL	Atlantic Forest	UFAL	-33.2
RDA034	2008	*Tangara cayana*	Thraupidae	Thraupinae	Body	-9.523	-36.290	AL	Atlantic Forest	UFAL	-58.8
RDA035	2008	*Euphonia violacea*	Fringilidae	Others	Body	-9.399	-36.145	AL	Atlantic Forest	UFAL	-43.4
RDA036	2009	*Nemosia pileata*	Thraupidae	Others	Body	-9.267	-37.936	AL	Caatinga	UFAL	-6.05
RDA037	2009	*Sporophila albogularis*	Thraupidae	Sporophilinae	Body	-9.561	-37.430	AL	Caatinga	UFAL	-47.5
RDA038	2009	*Paroaria dominicana*	Thraupidae	Thraupinae	Body	-9.561	-37.430	AL	Caatinga	UFAL	-35.2
RDA039	2009	*Coryphospingus pileatus*	Thraupidae	Tachyphoninae	Body	-9.561	-37.430	AL	Caatinga	UFAL	-50.8
RDA040	2009	*Coryphospingus pileatus*	Thraupidae	Tachyphoninae	Body	-9.267	-37.936	AL	Caatinga	UFAL	5.01
RDA041	2011	*Tachyphonus rufus*	Thraupidae	Tachyphoninae	Body	-8.464	-35.083	PE	Atlantic Forest	UFAL	-59.2
RDA042	2012	*Tangara cayana*	Thraupidae	Thraupinae	Body	-8.281	-35.031	PE	Atlantic Forest	UFAL	-54.1
RDA043	2015	*Coryphospingus pileatus*	Thraupidae	Tachyphoninae	Wing	-7.136	-38.506	PB	Caatinga	UNIVASF	-40.4
RDA044	2014	*Volatinia jacarina*	Thraupidae	Tachyphoninae	Wing	-7.490	-38.986	CE	Caatinga	UNIVASF	-49.4
RDA045	2014	*Coryphospingus pileatus*	Thraupidae	Tachyphoninae	Wing	-7.714	-39.007	CE	Caatinga	UNIVASF	-29.1
RDA046	2016	*Campsothraupis loricata*	Thraupidae	Others	Wing	-8.454	-37.914	PE	Caatinga	UNIVASF	-25.3
RDA047	2017	*Cyanoloxia brissonii*	Cardinalidae	Others	Wing	-8.729	-38.334	PE	Caatinga	UNIVASF	-41.3
RDA048	2013	*Tangara cayana*	Thraupidae	Thraupinae	Wing	-5.923	-35.176	RN	Atlantic Forest	UFRN	-43.5
RDA049	2013	*Sicalis luteola*	Thraupidae	Diglossinae	Wing	-5.293	-35.404	RN	Atlantic Forest	UFRN	-60.1
RDA050	2013	*Tangara cayana*	Thraupidae	Thraupinae	Wing	-5.293	-35.404	RN	Atlantic Forest	UFRN	-64.9
RDA051	2014	*Paroaria dominicana*	Thraupidae	Thraupinae	Wing	-5.390	-36.697	RN	Caatinga	UFRN	-44.6
RDA052	2014	*Coereba flaveola*	Thraupidae	Others	Wing	-5.923	-35.176	RN	Atlantic Forest	UFRN	-53.8
RDA053	2014	*Tangara sayaca*	Thraupidae	Thraupinae	Wing	-6.398	-38.381	RN	Caatinga	UFRN	-34.2
RDA054	2014	*Coryphospingus pileatus*	Thraupidae	Tachyphoninae	Wing	-6.398	-38.381	RN	Caatinga	UFRN	-51.7
RDA055	2015	*Paroaria dominicana*	Thraupidae	Thraupinae	Wing	-6.076	-36.647	RN	Caatinga	UFRN	-40.4
RDA056	2016	*Paroaria dominicana*	Thraupidae	Thraupinae	Wing	-6.076	-36.647	RN	Caatinga	UFRN	-38.5
RDA057	2015	*Coryphospingus pileatus*	Thraupidae	Tachyphoninae	Wing	-6.580	-37.255	RN	Caatinga	UFRN	-26.6
RDA058	2014	*Coryphospingus pileatus*	Thraupidae	Tachyphoninae	Wing	-6.580	-37.255	RN	Caatinga	UFRN	-31.0
RDA059	2017	*Tangara cayana*	Thraupidae	Thraupinae	Tail	-15.949	-47.942	DF	Cerrado	UNB	-55.5
RDA060	2014	*Coryphospingus pileatus*	Thraupidae	Tachyphoninae	Wing	-16.173	-42.291	MG	Atlantic Forest	UNB	-52.1
RDA061	2014	*Neothraupis fasciata*	Thraupidae	Thraupinae	Wing	-15.542	-47.614	DF	Cerrado	UNB	-32.9
RDA062	2014	*Sporophila albogularis*	Thraupidae	Sporophilinae	Wing	-16.173	-42.291	MG	Atlantic Forest	UNB	-49.2

ID	Year	Species	Family	Subfamily	Feather type	Lat	Long	State	Biome	Museum	δ^2H_f (‰)
RDA063	2016	*Sicalis flaveola*	Thraupidae	Diglossinae	Wing	-32.733	-52.576	RS	Pampa	FURG	-67.0
RDA064	2018	*Coryphospingus pileatus*	Thraupidae	Tachyphoninae	Tail	-8.148	-43.748	PI	Caatinga	UNB	-30.4
RDA065	2018	*Coryphospingus pileatus*	Thraupidae	Tachyphoninae	Tail	-7.761	-43.860	PI	Cerrado	UNB	-67.6
RDA066	2018	*Cyanoloxia brissonii*	Cardinalidae	Others	Tail	-7.761	-43.860	PI	Cerrado	UNB	-68.8
RDA067	2013	*Coryphospingus pileatus*	Thraupidae	Tachyphoninae	Wing	-14.458	-47.037	GO	Cerrado	UNB	-58.5
RDA068	2013	*Saltator similis*	Thraupidae	Saltatorinae	Wing	-15.615	-46.926	GO	Cerrado	UNB	-67.5
RDA069	2014	*Tangara cayana*	Thraupidae	Thraupinae	Wing	-18.592	-45.029	MG	Cerrado	UNB	-53.0
RDA070	2015	*Paroaria gularis*	Thraupidae	Thraupinae	Wing	-10.104	-67.755	AC	Amazon	UNB	-59.7
RDA071	2015	*Tangara cayana*	Thraupidae	Thraupinae	Tail	-15.717	-47.961	DF	Cerrado	UNB	-59.8
RDA072	2015	*Tangara cayana*	Thraupidae	Thraupinae	Tail	-12.864	-38.275	BA	Atlantic Forest	UNB	-51.2
RDA073	2015	*Tangara cayana*	Thraupidae	Thraupinae	Tail	-12.864	-38.275	BA	Atlantic Forest	UNB	-46.8
RDA074	2014	*Tangara cayana*	Thraupidae	Thraupinae	Tail	-15.731	-47.957	DF	Cerrado	UNB	-68.3
RDA075	2014	*Tangara cayana*	Thraupidae	Thraupinae	Tail	-23.098	-47.131	SP	Atlantic Forest	UNB	-45.0
RDA076	2014	*Tangara cayana*	Thraupidae	Thraupinae	Tail	-23.098	-47.131	SP	Atlantic Forest	UNB	-56.8
RDA077	2014	*Tangara sayaca*	Thraupidae	Thraupinae	Tail	-23.002	-47.137	SP	Atlantic Forest	UNB	-62.0
RDA078	2014	*Tangara sayaca*	Thraupidae	Thraupinae	Tail	-23.002	-47.137	SP	Atlantic Forest	UNB	-59.7
RDA079	2013	*Lanio surinamus*	Thraupidae	Tachyphoninae	Wing	-5.793	-57.400	PA	Amazon	GOELDI	-73.6
RDA080	2013	*Lanio surinamus*	Thraupidae	Tachyphoninae	Wing	-5.622	-57.281	PA	Amazon	GOELDI	-47.2
RDA081	2013	*Lanio surinamus*	Thraupidae	Tachyphoninae	Wing	-5.622	-57.281	PA	Amazon	GOELDI	-55.3
RDA082	2013	*Lanio surinamus*	Thraupidae	Tachyphoninae	Wing	-6.104	-57.657	PA	Amazon	GOELDI	-55.8
RDA083	2013	*Coryphospingus pileatus*	Thraupidae	Tachyphoninae	Wing	-8.718	-42.551	PI	Caatinga	GOELDI	-29.2
RDA084	2013	*Coryphospingus pileatus*	Thraupidae	Tachyphoninae	Wing	-8.844	-42.617	PI	Caatinga	GOELDI	-41.6
RDA085	2013	*Lanio luctuosus*	Thraupidae	Tachyphoninae	Wing	-3.704	-46.762	MA	Amazon	GOELDI	-42.9
RDA086	2013	*Tangara episcopus*	Thraupidae	Thraupinae	Wing	-3.704	-46.762	MA	Amazon	GOELDI	-55.4
RDA087	2013	*Lanio surinamus*	Thraupidae	Tachyphoninae	Wing	-0.133	-67.017	AM	Amazon	GOELDI	-80.0
RDA088	2013	*Lanio surinamus*	Thraupidae	Tachyphoninae	Wing	-0.133	-67.017	AM	Amazon	GOELDI	-61.5
RDA089	2013	*Tangara sayaca*	Thraupidae	Thraupinae	Wing	-7.349	-49.245	TO	Amazon	GOELDI	-64.6
RDA090	2013	*Tangara sayaca*	Thraupidae	Thraupinae	Wing	-7.349	-49.245	TO	Amazon	GOELDI	-53.0
RDA091	2014	*Tangara cyanicollis*	Thraupidae	Thraupinae	Wing	-9.896	-54.375	MT	Amazon	GOELDI	-45.8
RDA092	2014	*Tangara chilensis*	Thraupidae	Thraupinae	Wing	-9.777	-54.353	MT	Amazon	GOELDI	-51.3
RDA093	2014	*Tangara cyanocephala*	Thraupidae	Thraupinae	Wing	-12.448	-38.416	BA	Atlantic Forest	GOELDI	-44.7
RDA094	2014	*Coryphospingus pileatus*	Thraupidae	Tachyphoninae	Wing	-12.528	-40.307	BA	Caatinga	GOELDI	-31.6
RDA095	2014	*Coryphospingus pileatus*	Thraupidae	Tachyphoninae	Wing	-12.528	-40.307	BA	Caatinga	GOELDI	-35.8
RDA096	2014	*Tangara cyanocephala*	Thraupidae	Thraupinae	Wing	-12.448	-38.416	BA	Atlantic Forest	GOELDI	-42.6

ID	Year	Species	Family	Subfamily	Feather type	Lat	Long	State	Biome	Museum	δ^2H_f (‰)
RDA097	2013	*Tangara episcopus*	Thraupidae	Thraupinae	Wing	-8.316	-55.101	PA	Amazon	GOELDI	-87.6
RDA098	2015	*Lanio cristatus*	Thraupidae	Tachyphoninae	Wing	-11.109	-54.260	MT	Amazon	GOELDI	-34.4
RDA099	2014	*Tangara cyanocephala*	Thraupidae	Thraupinae	Wing	-15.413	-39.493	BA	Atlantic Forest	GOELDI	-57.0
RDA100	2014	*Tangara cyanocephala*	Thraupidae	Thraupinae	Wing	-15.413	-39.493	BA	Atlantic Forest	GOELDI	-54.8
RDA101	2014	*Lanio cristatus*	Thraupidae	Tachyphoninae	Wing	-14.347	-39.083	BA	Atlantic Forest	GOELDI	-48.5
RDA102	2015	*Lanio surinamus*	Thraupidae	Tachyphoninae	Wing	-2.055	-50.360	PA	Amazon	GOELDI	-81.7
RDA103	2015	*Lanio surinamus*	Thraupidae	Tachyphoninae	Wing	-2.055	-50.360	PA	Amazon	GOELDI	-44.0
RDA104	2014	*Coryphospingus cucullatus*	Thraupidae	Tachyphoninae	Wing	-2.719	-47.560	PA	Amazon	GOELDI	-79.9
RDA105	2014	*Coryphospingus cucullatus*	Thraupidae	Tachyphoninae	Wing	-2.719	-47.560	PA	Amazon	GOELDI	-74.1
RDA106	2015	*Tangara episcopus*	Thraupidae	Thraupinae	Wing	-1.738	-51.456	PA	Amazon	GOELDI	-94.9
RDA107	2015	*Tangara episcopus*	Thraupidae	Thraupinae	Wing	-1.738	-51.456	PA	Amazon	GOELDI	-57.8
RDA108	2015	*Lanio surinamus*	Thraupidae	Tachyphoninae	Wing	3.798	-51.878	AP	Amazon	GOELDI	-75.7
RDA109	2015	*Lanio surinamus*	Thraupidae	Tachyphoninae	Wing	3.798	-51.878	AP	Amazon	GOELDI	-44.7
RDA110	2016	*Tangara episcopus*	Thraupidae	Thraupinae	Wing	-1.853	-50.702	PA	Amazon	GOELDI	-74.4
RDA111	2017	*Tangara episcopus*	Thraupidae	Thraupinae	Wing	-1.155	-49.199	PA	Amazon	GOELDI	-80.0
RDA112	2017	*Tangara palmarum*	Thraupidae	Thraupinae	Wing	-7.838	-49.373	PA	Amazon	GOELDI	-52.4
RDA113	2017	*Tangara palmarum*	Thraupidae	Thraupinae	Wing	-7.927	-49.403	PA	Amazon	GOELDI	-56.4
RDA114	2018	*Tangara nigrocincta*	Thraupidae	Thraupinae	Wing	-10.504	-54.399	MT	Amazon	GOELDI	-62.8
RDA115	2018	*Tangara chilensis*	Thraupidae	Thraupinae	Wing	-10.360	-53.502	MT	Amazon	GOELDI	-65.7
RDA116	2018	*Tangara cyanicollis*	Thraupidae	Thraupinae	Wing	-12.775	-55.719	MT	Cerrado	GOELDI	-72.9
RDA117	2018	*Lanio surinamus*	Thraupidae	Tachyphoninae	Wing	-7.695	-60.912	AM	Amazon	GOELDI	-80.6
RDA118	2018	*Tangara mexicana*	Thraupidae	Thraupinae	Wing	-7.695	-60.912	AM	Amazon	GOELDI	-107.0
RDA119	2016	*Tangara sayaca*	Thraupidae	Thraupinae	Wing	-32.162	-52.185	RS	Pampa	FURG	-73.0
RDA120	2015	*Paroaria coronata*	Thraupidae	Thraupinae	Wing	-31.072	-52.75	RS	Pampa	FURG	-78.8
RDA121	2015	*Tangara sayaca*	Thraupidae	Thraupinae	Wing	-32.087	-52.159	RS	Pampa	FURG	-64.3
RDA122	2013	*Coereba flaveola*	Thraupidae	Others	Wing	-30.058	-51.174	RS	Pampa	PUCRS	-61.4
RDA124	2013	*Pyrrhocoma ruficeps*	Thraupidae	Others	Wing	-30.050	-51.308	RS	Pampa	PUCRS	-69.3
RDA125	2013	*Porphyrospiza caerulescens*	Thraupidae	Others	Wing	-20.147	-46.659	MG	Cerrado	PUCRS	-79.6
RDA126	2013	*Schistochlamys ruficapillus*	Thraupidae	Thraupinae	Wing	-28.545	-51.396	PR	Atlantic Forest	PUCRS	-62.7
RDA127	2014	*Sicalis flaveola*	Thraupidae	Diglossinae	Wing	-30.216	-57.48	RS	Pampa	PUCRS	-97.4
RDA128	2013	*Ramphocelus bresilius*	Thraupidae	Tachyphoninae	Wing	-22.993	-42.016	RJ	Atlantic Forest	PUCRS	-49.3
RDA129	2018	*Sicalis flaveola*	Thraupidae	Diglossinae	Wing	-20.590	-40.459	ES	Atlantic Forest	UVV	-60.9
RDA130	2018	*Sicalis flaveola*	Thraupidae	Diglossinae	Wing	-20.590	-40.459	ES	Atlantic Forest	UVV	-57.5
RDA131	2017	*Sicalis flaveola*	Thraupidae	Diglossinae	Body	-21.150	-56.472	MS	Cerrado	UNB	-79.0

ID	Year	Species	Family	Subfamily	Feather type	Lat	Long	State	Biome	Museum	δ^2H_f (‰)
RDA132	2015	*Sicalis flaveola*	Thraupidae	Diglossinae	Body	-21.150	-56.472	MS	Cerrado	UNB	-88.7
RDA133	2017	*Sicalis flaveola*	Thraupidae	Diglossinae	Wing	-19.570	-56.139	MS	Pantanal	UNB	-81.3
RDA134	2017	*Sicalis flaveola*	Thraupidae	Diglossinae	Wing	-19.570	-56.139	MS	Pantanal	UNB	-98.8
RDA135	2017	*Paroaria capitata*	Thraupidae	Thraupinae	Body	-19.570	-56.139	MS	Pantanal	UNB	-59.2
RDA136	2017	*Paroaria capitata*	Thraupidae	Thraupinae	Body	-19.570	-56.139	MS	Pantanal	UNB	-25.1
RDA137	2017	*Tangara cayana*	Thraupidae	Thraupinae	Body	-17.718	-42.444	MG	Cerrado	UNB	-52.0
RDA138	2017	*Tangara cayana*	Thraupidae	Thraupinae	Body	-17.718	-42.444	MG	Cerrado	UNB	-53.4
RDA139	2017	*Sicalis flaveola*	Thraupidae	Diglossinae	Wing	-17.718	-42.444	MG	Cerrado	UNB	-59.0
RDA140	2017	*Sicalis flaveola*	Thraupidae	Diglossinae	Wing	-17.718	-42.444	MG	Cerrado	UNB	-66.5
RDA141	2017	*Sicalis flaveola*	Thraupidae	Diglossinae	Body	-16.678	-49.409	GO	Cerrado	UNB	-52.1
RDA142	2017	*Sicalis flaveola*	Thraupidae	Diglossinae	Wing	-16.678	-49.409	GO	Cerrado	UNB	-93.1
RDA143	2017	*Sporophila nigricollis*	Thraupidae	Sporophilinae	Body	-16.678	-49.409	GO	Cerrado	UNB	-38.9
RDA144	2017	*Sporophila nigricollis*	Thraupidae	Sporophilinae	Body	-16.678	-49.409	GO	Cerrado	UNB	-54.4
RDA145	2016	*Sicalis flaveola*	Thraupidae	Diglossinae	Body	-16.379	-56.621	MT	Pantanal	UNB	-101.0
RDA146	2016	*Paroaria capitata*	Thraupidae	Thraupinae	Body	-16.379	-56.621	MT	Pantanal	UNB	-42.8
RDA147	2016	*Sicalis flaveola*	Thraupidae	Diglossinae	Body	-16.389	-56.591	MT	Pantanal	UNB	-83.7
RDA148	2016	*Sicalis flaveola*	Thraupidae	Diglossinae	Body	-16.364	-56.630	MT	Pantanal	UNB	-71.8
RDA149	2016	*Sicalis flaveola*	Thraupidae	Diglossinae	Body	-16.364	-56.630	MT	Pantanal	UNB	-87.3
RDA150	2015	*Tangara cayana*	Thraupidae	Thraupinae	Body	-15.931	-47.902	DF	Cerrado	UNB	-78.3
RDA151	2015	*Tangara cayana*	Thraupidae	Thraupinae	Body	-15.931	-47.902	DF	Cerrado	UNB	-54.3
RDA152	2017	*Tangara cayana*	Thraupidae	Thraupinae	Body	-15.775	-48.822	GO	Cerrado	UNB	-63.7
RDA153	2017	*Tangara cayana*	Thraupidae	Thraupinae	Body	-15.775	-48.822	GO	Cerrado	UNB	-67.1
RDA154	2018	*Sicalis flaveola*	Thraupidae	Diglossinae	Wing	-15.775	-48.822	GO	Cerrado	UNB	-48.9
RDA155	2018	*Sicalis flaveola*	Thraupidae	Diglossinae	Wing	-15.775	-48.822	GO	Cerrado	UNB	-61.4
RDA156	2017	*Sicalis flaveola*	Thraupidae	Diglossinae	Wing	-15.402	-55.809	MT	Cerrado	UNB	-56.2
RDA157	2017	*Sicalis flaveola*	Thraupidae	Diglossinae	Wing	-15.302	-55.834	MT	Cerrado	UNB	-60.8
RDA158	2018	*Coryphospingus cucullatus*	Thraupidae	Tachyphoninae	Body	-15.302	-55.834	MT	Cerrado	UNB	-81.0
RDA159	2018	*Coryphospingus cucullatus*	Thraupidae	Tachyphoninae	Body	-15.406	-55.832	MT	Cerrado	UNB	-60.9
RDA160	2017	*Sicalis flaveola*	Thraupidae	Diglossinae	Wing	-14.148	-47.737	GO	Cerrado	UNB	-68.7
RDA161	2017	*Sicalis flaveola*	Thraupidae	Diglossinae	Wing	-14.148	-47.737	GO	Cerrado	UNB	-82.3
RDA162	2017	*Tangara cayana*	Thraupidae	Thraupinae	Body	-14.167	-47.735	GO	Cerrado	UNB	-63.7
RDA163	2017	*Tangara cayana*	Thraupidae	Thraupinae	Body	-14.167	-47.735	GO	Cerrado	UNB	-55.1
RDA164	2017	*Sicalis flaveola*	Thraupidae	Diglossinae	Wing	-7.332	-46.683	MA	Cerrado	UNB	-66.5
RDA165	2017	*Sicalis flaveola*	Thraupidae	Diglossinae	Wing	-7.332	-46.683	MA	Cerrado	UNB	-96.9

ID	Year	Species	Family	Subfamily	Feather type	Lat	Long	State	Biome	Museum	δ^2H_f (‰)
RDA166	2015	*Tangara cayana*	Thraupidae	Thraupinae	Body	-7.133	-47.129	MA	Cerrado	UNB	-48.9
RDA167	2015	*Tangara cayana*	Thraupidae	Thraupinae	Body	-7.133	-47.129	MA	Cerrado	UNB	-42.8
RDA168	2014	*Sicalis citrina*	Thraupidae	Diglossinae	Body	-0.385	-51.050	AP	Amazon	UNB	-75.7
RDA169	2014	*Sicalis citrina*	Thraupidae	Diglossinae	Body	-0.385	-51.050	AP	Amazon	UNB	-80.4
RDA170	2016	*Lanio surinamus*	Thraupidae	Tachyphoninae	Tail	-1.489	-59.787	AM	Amazon	INPA	-60.5
RDA171	2016	*Lanio surinamus*	Thraupidae	Tachyphoninae	Tail	-1.684	-59.654	AM	Amazon	INPA	-85.2
RDA172	2016	*Tangara sayaca*	Thraupidae	Thraupinae	Tail	-12.905	-38.322	BA	Atlantic Forest	UNB	-55.7
RDA173	2016	*Tangara sayaca*	Thraupidae	Thraupinae	Tail	-12.905	-38.322	BA	Atlantic Forest	UNB	-45.7
RDA174	2009	*Sporophila albogularis*	Thraupidae	Sporophilinae	Body	-9.561	-37.430	AL	Caatinga	UFAL	-40.3
RDA175	2017	*Coryphospingus pileatus*	Thraupidae	Tachyphoninae	Body	-9.534	-37.827	AL	Caatinga	UFAL	-26.7
RDA176	2014	*Tachyphonus surinamus*	Thraupidae	Tachyphoninae	Body	-2.317	-55.333	PA	Amazon	INPA	-61.1
RDA177	2013	*Tachyphonus surinamus*	Thraupidae	Tachyphoninae	Body	-2.317	-55.333	PA	Amazon	INPA	-59.7
RDA178	2013	*Tachyphonus surinamus*	Thraupidae	Tachyphoninae	Body	-2.317	-55.333	PA	Amazon	INPA	-60.0
RDA179	2013	*Lanio penicillata*	Thraupidae	Tachyphoninae	Body	-2.609	-60.876	AM	Amazon	INPA	-60.6
RDA180	2013	*Lanio penicillata*	Thraupidae	Tachyphoninae	Body	-2.686	-60.770	AM	Amazon	INPA	-47.4
RDA181	2013	*Lanio penicillata*	Thraupidae	Tachyphoninae	Body	-2.609	-60.876	AM	Amazon	INPA	-57.9
RDA182	2016	*Saltator maximus*	Thraupidae	Saltatorinae	Body	1.600	-61.900	RR	Amazon	INPA	-48.7
RDA183	2016	*Saltator maximus*	Thraupidae	Saltatorinae	Body	1.600	-61.900	RR	Amazon	INPA	-45.2
RDA184	2015	*Saltator maximus*	Thraupidae	Saltatorinae	Body	-3.054	-60.083	AM	Amazon	INPA	-61.7
RDA185	2015	*Tangara palmarum*	Thraupidae	Thraupinae	Body	-3.097	-59.987	AM	Amazon	INPA	-72.7
RDA186	2014	*Tangara palmarum*	Thraupidae	Thraupinae	Body	-3.095	-59.988	AM	Amazon	INPA	-93.5
RDA187	2015	*Tangara palmarum*	Thraupidae	Thraupinae	Body	-3.097	-59.987	AM	Amazon	INPA	-47.7
RDA188	2016	*Tachyphonus cristatus*	Thraupidae	Tachyphoninae	Body	-3.003	-59.933	AM	Amazon	INPA	-60.4
RDA189	2007	*Tachyphonus cristatus*	Thraupidae	Tachyphoninae	Body	-1.877	-61.586	AM	Amazon	INPA	-47.2
RDA190	2007	*Tachyphonus cristatus*	Thraupidae	Tachyphoninae	Body	-0.065	-64.098	AM	Amazon	INPA	-51.4
RDA191	2017	*Tangara cayana*	Thraupidae	Thraupinae	Wing	-18.681	-50.454	GO	Atlantic Forest	UNB	-71.8
RDA192	2017	*Tangara cayana*	Thraupidae	Thraupinae	Wing	-18.681	-50.454	GO	Atlantic Forest	UNB	-55.1
RDA193	2015	*Tangara sayaca*	Thraupidae	Thraupinae	Tail	-15.881	-47.944	DF	Cerrado	UNB	-52.5

Museum*: UFBA- Universidade Federal da Bahia; UFMT- Universidade Federal de Mato Grosso; UFAL- Universidade Federal de Alagoas; UNIVASF: Universidade Federal do Vale do São Francisco; UFRN- Universidade Federal do Rio Grande do Norte; UNB- Universidade de Brasília; FURG- Universidade Federal do Rio Grande; GOELDI- Museu Paraense Emílio Goeldi; PUCRS- Pontifícia Universidade Católica do Rio Grande do Sul; UVV- Universidade Vila Velha; e INPA: Instituto Nacional de Pesquisas da Amazônia. In case of UNB, samples came either from ornithological museum or ongoing projects.

1. Piacentini V de Q, Aleixo A, Agne CE, Maurício GN, Pacheco JF, Bravo GA, et al. Annotated checklist of the birds of Brazil by the Brazilian Ornithological Records Committee. Rev Bras Ornitol. 2015;23: 91–298. doi:urn:lsid:zoobank.org:pub:30856542-FFD1-44CA-B249-9F321CD4CF4C
