# Supplementary material for: A feather hydrogen (δ2H) isoscape for Brazil
Source: PLoS One. 2022 Aug 3;17(8):e0271573. doi: 10.1371/journal.pone.0271573 (PMC9348672; doi:10.1371/journal.pone.0271573)
Supplement: S1 File — R code and results for Analysis of Variance followed by a Tukey’s HSD (variable year of collection, feather type, biome, and sub-family), Linear Models (variable latitude and longitude), and Model Selection with all variables. (PDF) [file pone.0271573.s002.pdf]

S2 File. Exploratory analysis.

R code and results for Analysis of Variance followed by a Tukey's HSD (variable year of collection, feather type, biome, and sub-family), Linear Models (variable latitude and longitude), and Model Selection with all variables.

```
Data<-read.table("Feather_jan2020.txt",h=T)
shapiro.test(Data$d2H)
##  Shapiro-Wilk normality test
## data:  Data$d2H
## W = 0.98674, p-value = 0.06889
```

❖ Analysis of Variance (Biome, Feather type, Subfamily and Year of collection)

1- Biome

```
A<-aov(d2H~Biome, data=Data)
A
## Call:
##    aov(formula = d2H ~ Biome, data = Data)
## Terms:
##                    Biome Residuals
## Sum of Squares  23125.02  39256.94
## Deg. of Freedom        5       186
## Residual standard error: 14.52786
## Estimated effects may be unbalanced

TukeyHSD(A)
##   Tukey multiple comparisons of means
##     95% family-wise confidence level
## Fit: aov(formula = d2H ~ Biome, data = Data)
## $Biome
##                              diff        lwr        upr     p adj
## Caatinga-Amazonia        27.893904  18.406229  37.381579 0.0000000
## Cerrado-Amazonia         -1.270930  -9.356885   6.815024 0.9975834
## Mata_Atlantica-Amazonia  12.010316   3.237234  20.783399 0.0015752
## Pampa-Amazonia           -8.975012 -25.699022   7.748997 0.6353976
## Pantanal-Amazonia        -6.239168 -19.978643   7.500307 0.7805322
## Cerrado-Caatinga        -29.164835 -38.966361 -19.363308 0.0000000
## Mata_Atlantica-Caatinga -15.883588 -26.259244  -5.507932 0.0002518
## Pampa-Caatinga          -36.868916 -54.486514 -19.251318 0.0000001
## Pantanal-Caatinga       -34.133072 -48.947269 -19.318875 0.0000000
## Mata_Atlantica-Cerrado   13.281247   4.169665  22.392828 0.0005897
## Pampa-Cerrado            -7.704082 -24.608118   9.199955 0.7778954
## Pantanal-Cerrado         -4.968237 -18.926286   8.989811 0.9090667
## Pampa-Mata_Atlantica    -20.985328 -38.228610  -3.742047 0.0074562
## Pantanal-Mata_Atlantica -18.249484 -32.616510  -3.882458 0.0044112
## Pantanal-Pampa            2.735844 -17.491290  22.962978 0.9988250

boxplot(d2H~Biome, data=Data)
```

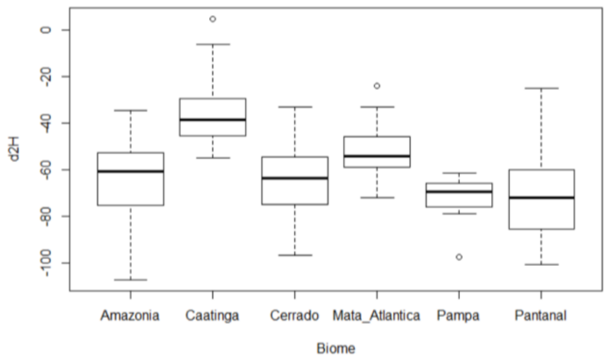

2- Feather Type

```
B<-aov(d2H~Feather_Type, data=Data)
B
## Call:
##    aov(formula = d2H ~ Feather_Type, data = Data)
## Terms:
##                 Feather_Type Residuals
## Sum of Squares       1765.28  60616.67
## Deg. of Freedom            2       189
## Residual standard error: 17.90874
## Estimated effects may be unbalanced

TukeyHSD(B)

##   Tukey multiple comparisons of means
##     95% family-wise confidence level
## Fit: aov(formula = d2H ~ Feather_Type, data = Data)
## $Feather_Type
##                diff       lwr        upr     p adj
## Tail-Body -4.685738 -15.91732 6.54584180 0.5868945
## Wing-Body -6.532104 -13.12309 0.05888223 0.0526669
## Wing-Tail -1.846366 -12.62421 8.93147870 0.9137301

boxplot(d2H~Feather_Type, data=Data)
```

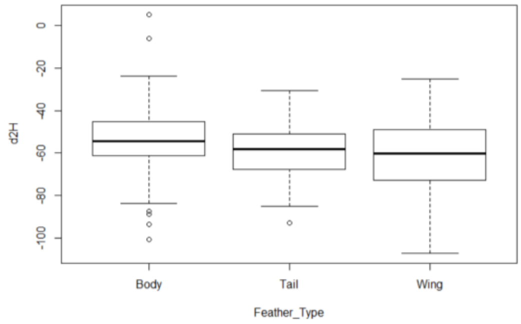

3- Subfamily

```
C<-aov(d2H~Sub_Family, data=Data)
C
## Call:
##    aov(formula = d2H ~ Sub_Family, data = Data)
## Terms:
##                 Sub_Family Residuals
## Sum of Squares    11228.94  51153.02
## Deg. of Freedom          6       185
## Residual standard error: 16.62837
## Estimated effects may be unbalanced

TukeyHSD(C)
##   Tukey multiple comparisons of means
##     95% family-wise confidence level
## Fit: aov(formula = d2H ~ Sub_Family, data = Data)
## $Sub_Family
##                                    diff         lwr       upr     p adj
## Diglossinae-Dacninae        -10.58264286 -34.6492266 13.483941 0.8461932
## Others-Dacninae              15.60472727 -11.1315010 42.340956 0.5902050
## Saltatorinae-Dacninae         5.40200000 -25.9490067 36.753007 0.9986391
## Sporophilinae-Dacninae        8.88325000 -19.3761655 37.142666 0.9660321
## Tachyphoninae-Dacninae       12.35378571 -10.7832329 35.490804 0.6878129
## Thraupinae-Dacninae           5.49832911 -17.3609531 28.357611 0.9914294
## Others-Diglossinae           26.18737013   8.5481882 43.826552 0.0003255
## Saltatorinae-Diglossinae     15.98464286  -8.0819409 40.051227 0.4309216
## Sporophilinae-Diglossinae    19.46589286  -0.4064346 39.338220 0.0590721
```

```
## Tachyphoninae-Diglossinae    22.93642857  11.4631349 34.409722 0.0000003
## Thraupinae-Diglossinae       16.08097197   5.1786044 26.983340 0.0003662
## Saltatorinae-Others         -10.20272727 -36.9389556 16.533501 0.9156314
## Sporophilinae-Others         -6.72147727 -29.7548194 16.311865 0.9765530
## Tachyphoninae-Others         -3.25094156 -19.5990888 13.097206 0.9969619
## Thraupinae-Others           -10.10639816 -26.0590490  5.846253 0.4904959
## Sporophilinae-Saltatorinae    3.48125000 -24.7781655 31.740666 0.9998023
## Tachyphoninae-Saltatorinae    6.95178571 -16.1852329 30.088804 0.9728462
## Thraupinae-Saltatorinae       0.09632911 -22.7629531 22.955611 1.0000000
## Tachyphoninae-Sporophilinae   3.47053571 -15.2652743 22.206346 0.9979546
## Thraupinae-Sporophilinae     -3.38492089 -21.7766508 15.006809 0.9980272
## Thraupinae-Tachyphoninae     -6.85545660 -15.5147174  1.803804 0.2217738
```

```
boxplot(d2H~Sub_Family, data=Data)
```

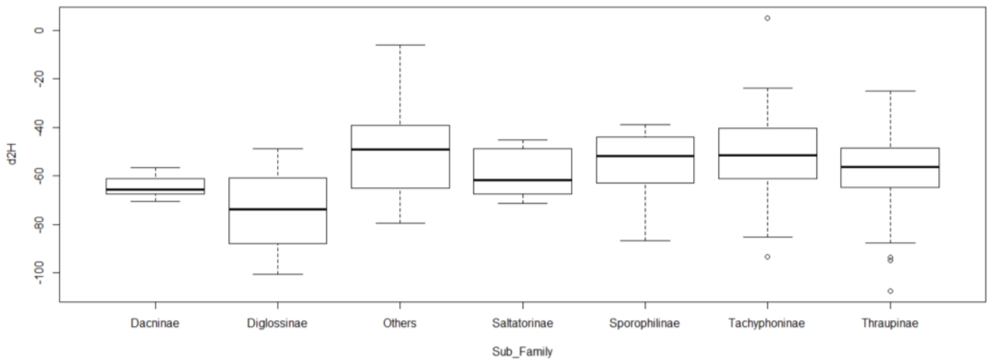

4- Year of collection

```
D<-aov(d2H~as.factor(Year), data=Data)
D
## Call:
##    aov(formula = d2H ~ as.factor(Year), data = Data)
## Terms:
##                 as.factor(Year) Residuals
## Sum of Squares          9602.03  52779.93
## Deg. of Freedom              11       180
## Residual standard error: 17.12372
## Estimated effects may be unbalanced
```

```
TukeyHSD(D)
##   Tukey multiple comparisons of means
##     95% family-wise confidence level
## Fit: aov(formula = d2H ~ as.factor(Year), data = Data)
## $`as.factor(Year)`
##                   diff        lwr        upr     p adj
## 2008-2007   4.777000000  -33.26336  42.817363 0.9999996
## 2009-2007  19.970000000  -16.63436  56.574356 0.8123412
## 2011-2007 -10.125000000  -73.52560  53.275605 0.9999952
## 2012-2007  -4.965000000  -68.36560  58.435605 1.0000000
## 2013-2007 -10.115967742  -40.24336  20.011427 0.9938243
## 2014-2007  -9.250526316  -39.05910  20.558051 0.9968716
## 2015-2007  -7.943214286  -38.25450  22.368072 0.9993294
## 2016-2007 -12.723000000  -43.78283  18.336826 0.9699874
## 2017-2007 -11.687894737  -41.49647  18.120682 0.9784054
## 2018-2007 -15.080555556  -46.42665  16.265542 0.9095128
## 2019-2007 -40.795000000  -89.90490   8.314897 0.2107137
## 2009-2008  15.193000000  -19.14493  49.530930 0.9479262
## 2011-2008 -14.902000000  -77.02165  47.217652 0.9997108
## 2012-2008  -9.742000000  -71.86165  52.377652 0.9999960
## 2013-2008 -14.892967742  -42.22197  12.436036 0.8134534
## 2014-2008 -14.027526316  -41.00466  12.949608 0.8558248
## 2015-2008 -12.720214286  -40.25181  14.811379 0.9302789
## 2016-2008 -17.500000000  -45.85361  10.853612 0.6627790
## 2017-2008 -16.464894737  -43.44203  10.512240 0.6784678
```

```
## 2018-2008 -19.857555556  -48.52448   8.809366 0.4847713
## 2019-2008 -45.572000000  -93.01667   1.872668 0.0725588
## 2011-2009 -30.095000000  -91.34580  31.155803 0.8968569
## 2012-2009 -24.935000000  -86.18580  36.315803 0.9713357
## 2013-2009 -30.085967742  -55.37795  -4.793990 0.0063735
## 2014-2009 -29.220526316  -54.13188  -4.309174 0.0077575
## 2015-2009 -27.913214286  -53.42396  -2.402465 0.0189509
## 2016-2009 -32.693000000  -59.08878  -6.297223 0.0035032
## 2017-2009 -31.657894737  -56.56925  -6.746542 0.0023318
## 2018-2009 -35.050555556  -61.78260  -8.318513 0.0013813
## 2019-2009 -60.765000000 -107.06626 -14.463745 0.0013596
## 2012-2011   5.160000000  -75.03613  85.356126 1.0000000
## 2013-2011   0.009032258  -57.60557  57.623630 1.0000000
## 2014-2011   0.874473684  -56.57405  58.323001 1.0000000
## 2015-2011   2.181785714  -55.52918  59.892756 1.0000000
## 2016-2011  -2.598000000  -60.70561  55.509614 1.0000000
## 2017-2011  -1.562894737  -59.01142  55.885632 1.0000000
## 2018-2011  -4.955555556  -63.21669  53.305580 1.0000000
## 2019-2011 -30.670000000 -100.12188  38.781883 0.9486164
## 2013-2012  -5.150967742  -62.76557  52.463630 1.0000000
## 2014-2012  -4.285526316  -61.73405  53.163001 1.0000000
## 2015-2012  -2.978214286  -60.68918  54.732756 1.0000000
## 2016-2012  -7.758000000  -65.86561  50.349614 0.9999993
## 2017-2012  -6.722894737  -64.17142  50.725632 0.9999998
## 2018-2012 -10.115555556  -68.37669  48.145580 0.9999885
## 2019-2012 -35.830000000 -105.28188  33.621883 0.8621771
## 2014-2013   0.865441426  -12.85886  14.589744 1.0000000
## 2015-2013   2.172753456  -12.61167  16.957182 0.9999980
## 2016-2013  -2.607032258  -18.87104  13.656978 0.9999950
## 2017-2013  -1.571926995  -15.29623  12.152376 0.9999999
## 2018-2013  -4.964587814  -21.76885  11.839670 0.9979800
## 2019-2013 -30.679032258  -72.05037  10.692301 0.3748614
## 2015-2014   1.307312030  -12.81610  15.430721 1.0000000
## 2016-2014  -3.472473684  -19.13802  12.193074 0.9998660
## 2017-2014  -2.437368421  -15.44690  10.572161 0.9999751
## 2018-2014  -5.830029240  -22.05576  10.395705 0.9891660
## 2019-2014 -31.544473684  -72.68422   9.595271 0.3229079
## 2016-2015  -4.779785714  -21.38196  11.822390 0.9984006
## 2017-2015  -3.744680451  -17.86809  10.378729 0.9992516
## 2018-2015  -7.137341270  -24.26910   9.994421 0.9660203
## 2019-2015 -32.851785714  -74.35722   8.653653 0.2759700
## 2017-2016   1.035105263  -14.63044  16.700653 1.0000000
## 2018-2016  -2.357555556  -20.78134  16.066233 0.9999995
## 2019-2016 -28.072000000  -70.12720  13.983204 0.5443100
## 2018-2017  -3.392660819  -19.61839  12.833073 0.9999248
## 2019-2017 -29.107105263  -70.24685  12.032640 0.4504263
## 2019-2018 -25.714444444  -67.98151  16.552625 0.6828736
```

```
boxplot(d2H~Year, data=Data)
```

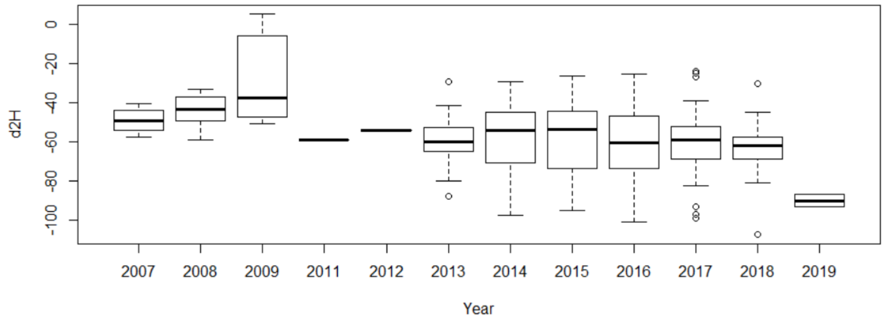

❖ Linear Models (Year, Latitude and Longitude)
5- Year

```
E<-lm(d2H~Year, data=Data)
summary(E)
## Call:
## lm(formula = d2H ~ Year, data = Data)
##
## Residuals:
##     Min      1Q  Median      3Q     Max
## -41.957 -10.434   0.757  11.195  51.567
## Coefficients:
##              Estimate Std. Error t value Pr(>|t|)
## (Intercept) 4153.7753   983.7516   4.222 3.74e-05 ***
## Year          -2.0908     0.4883  -4.282 2.94e-05 ***
## ---
## Signif. codes:  0 '***' 0.001 '**' 0.01 '*' 0.05 '.' 0.1 ' ' 1
##
## Residual standard error: 17.3 on 190 degrees of freedom
## Multiple R-squared:  0.08801,    Adjusted R-squared:  0.08321
## F-statistic: 18.33 on 1 and 190 DF,  p-value: 2.938e-05

plot(d2H~Year, data=Data)
abline(4153.775,-2.091)
```

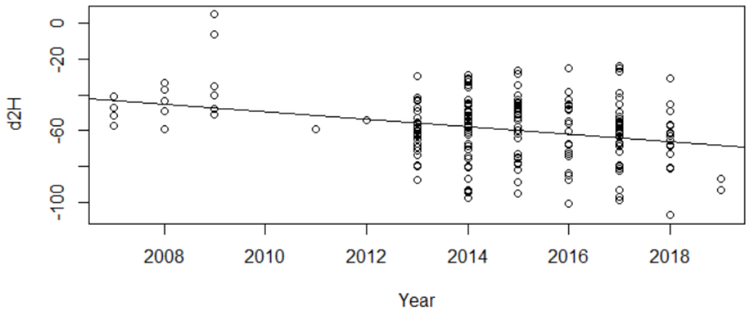


```
plot(a$residuals)
```

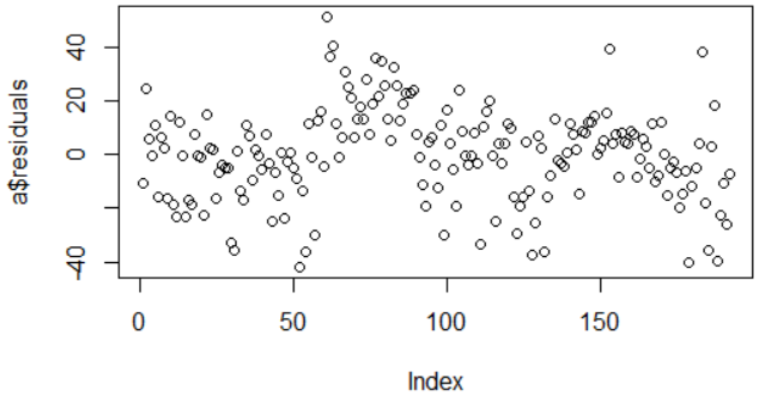


```
shapiro.test(a$residuals)

##      Shapiro-Wilk normality test
## data:  a$residuals
## W = 0.99012, p-value = 0.2092
```

```
data.frame(Data$Year,a$residuals)
```

OBS: This table was organized to allow better visualization.

Data.Year	a.residuals	Data.Year	a.residuals	Data.Year	a.residuals	Data.Year	a.residuals
**2007**	-4.7749	**2014**	-10.3796	**2015**	11.4412	**2017**	7.7627
**2007**	-9.0449	**2014**	5.7004	**2015**	31.0412	**2017**	11.1927
**2007**	1.8451	**2014**	11.2004	**2015**	18.7412	**2017**	-29.8573
**2007**	-14.9449	**2014**	-18.6896	**2015**	32.4912	**2017**	-5.3773
**2008**	-4.6241	**2014**	-23.3696	**2015**	18.7412	**2017**	-19.0373
**2008**	1.0359	**2014**	-22.9296	**2015**	-0.7088	**2017**	24.3627
**2008**	11.3059	**2014**	-17.0596	**2015**	-19.1888	**2017**	8.8827
**2008**	7.3359	**2014**	-4.0696	**2015**	4.7912	**2017**	-0.3873
**2008**	-14.3641	**2014**	-36.5196	**2015**	6.5912	**2017**	-3.8073
**2009**	-4.2634	**2014**	-0.8696	**2015**	10.2012	**2017**	-0.3673
**2009**	51.5666	**2014**	-29.8996	**2015**	16.2812	**2017**	8.1527
**2009**	40.5066	**2014**	25.4204	**2015**	-29.6188	**2017**	-3.2373
**2009**	11.4066	**2014**	21.1904	**2015**	-15.8188	**2017**	-33.5673
**2009**	-0.9734	**2014**	27.9104	**2015**	13.4612	**2017**	4.3127
**2009**	6.2866	**2014**	7.6304	**2015**	7.9312	**2017**	-3.2173
**2011**	-8.4918	**2014**	5.3004	**2015**	12.2912	**2017**	11.3227
**2012**	-1.2411	**2014**	26.0504	**2015**	-19.6688	**2017**	9.9327
**2013**	12.0697	**2014**	12.4404	**2015**	-5.1988	**2017**	-15.7273
**2013**	-0.5203	**2014**	22.7904	**2016**	-6.8480	**2017**	7.1227
**2013**	-18.7103	**2014**	24.1604	**2016**	-13.2180	**2017**	2.5227
**2013**	7.7697	**2014**	-11.2996	**2016**	0.6920	**2017**	39.4027
**2013**	-0.3303	**2014**	4.0104	**2016**	-24.0080	**2017**	-8.4873
**2013**	-0.8803	**2014**	-13.5496	**2016**	0.8220	**2017**	8.1727
**2013**	2.3997	**2014**	-37.6696	**2016**	12.5320	**2017**	4.1227
**2013**	-16.4303	**2014**	-36.1996	**2016**	16.0120	**2017**	38.2227
**2013**	-4.8203	**2014**	8.4704	**2016**	6.3320	**2017**	-18.0473
**2013**	-5.1203	**2014**	12.2704	**2016**	13.3320	**2017**	-35.5573
**2013**	-32.7203	**2014**	14.3904	**2016**	35.8920	**2017**	2.9227
**2013**	-9.6303	**2014**	0.0304	**2016**	22.7220	**2018**	-0.2765
**2013**	1.9397	**2014**	2.1904	**2016**	5.4720	**2018**	2.5335
**2013**	-5.6403	**2014**	4.9504	**2016**	15.5020	**2018**	-15.2665
**2013**	7.5597	**2014**	7.8304	**2016**	-5.8480	**2018**	-41.9565
**2013**	-2.9703	**2014**	3.2104	**2016**	-11.8080	**2018**	34.9435
**2013**	-25.0603	**2014**	12.0604	**2016**	18.3620	**2018**	16.4735
**2013**	-6.5403	**2014**	0.1704	**2016**	-39.4580	**2018**	3.9835
**2013**	25.7397	**2014**	-4.9396	**2016**	-22.4880	**2018**	20.3135
**2013**	13.2997	**2014**	-2.6696	**2016**	-10.5980	**2018**	-0.3465
**2013**	-3.5403	**2014**	-40.3796	**2016**	-26.1180	**2018**	-15.6565
**2013**	-12.5403	**2015**	24.6912	**2017**	-15.9473	**2018**	4.4835
**2013**	-24.6803	**2015**	-16.5688	**2017**	6.2527	**2018**	-7.5565
**2013**	5.6597	**2015**	14.3912	**2017**	1.8427	**2018**	-2.2565
**2013**	-5.2103	**2015**	-22.6388	**2017**	-16.7573	**2018**	-3.4565
**2013**	11.4297	**2015**	15.0812	**2017**	10.8627	**2018**	4.4535
**2013**	-9.9403	**2015**	-35.8388	**2017**	6.8527	**2018**	7.8635
**2013**	-7.7703	**2015**	1.3012	**2017**	36.5827	**2018**	4.3835
**2013**	-6.5103	**2015**	-0.5988	**2017**	17.7727	**2018**	8.7235
**2013**	-14.3503	**2015**	-2.5488	**2017**	13.4327	**2019**	-19.3458
**2013**	-7.2903	**2015**	-13.5888	**2017**	21.9827	**2019**	-25.5258

6- Latitude

```
summary(lm(d2H~Lat_GD, data=Data))
## Call:
## lm(formula = d2H ~ Lat_GD, data = Data)
## Residuals:
##     Min      1Q  Median      3Q     Max
## -49.979  -9.291   1.273  10.717  62.845
## Coefficients:
##             Estimate Std. Error t value Pr(>|t|)
## (Intercept)  -54.981      2.420  -22.72   <2e-16 ***
## Lat_GD         0.308      0.177    1.74   0.0835 .
## ---
## Signif. codes:  0 '***' 0.001 '**' 0.01 '*' 0.05 '.' 0.1 ' ' 1
##
## Residual standard error: 17.98 on 190 degrees of freedom
## Multiple R-squared:  0.01568,    Adjusted R-squared:  0.0105
## F-statistic: 3.027 on 1 and 190 DF,  p-value: 0.08351

plot(d2H~Lat_GD, data=Data)
abline(-54.98,0.308)
```

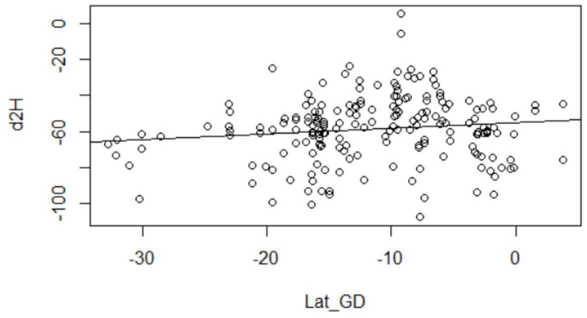

7- Longitude

```
summary(lm(d2H~Long_GD, data=Data))
## Call:
## lm(formula = d2H ~ Long_GD, data = Data)
## Residuals:
##     Min      1Q  Median      3Q     Max
## -40.542 -10.556   0.157   9.926  51.384
## Coefficients:
##             Estimate Std. Error t value Pr(>|t|)
## (Intercept)  -3.2862     6.7147  -0.489    0.625
## Long_GD       1.1358     0.1361   8.345 1.44e-14 ***
## ---
## Signif. codes:  0 '***' 0.001 '**' 0.01 '*' 0.05 '.' 0.1 ' ' 1
##
## Residual standard error: 15.5 on 190 degrees of freedom
## Multiple R-squared:  0.2682, Adjusted R-squared:  0.2643
## F-statistic: 69.63 on 1 and 190 DF,  p-value: 1.443e-14

plot(d2H~Long_GD, data=Data)
abline(-3.286,1.13)
```

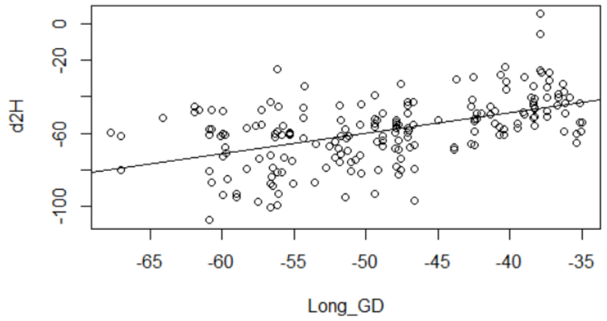

❖ Model Selection

```
library(MuMIn)
my_model<-dredge(lm(d2H~Year+Feather_Type+Long_GD+Lat_GD+Biome+Sub_Family, data=Data,na.action="na.f
ail"))
print(my_model)
```

```
## Global model call: lm(formula = d2H ~ Year + Feather_Type + Long_GD + Lat_GD + Biome
+ Sub_Family, data = Data, na.action = "na.fail")
## ---
## Model selection table
##        (Int) Bim Fth_Typ Lat_GD Lng_GD Sub_Fml Year df  logLik   AICc  delta  weight
## 28  -31.95  +     +           0.655       +        16 -763.259 1561.6   0.00  0.230
## 32  -28.13  +     +     -0.356 0.765       +        17 -762.370 1562.3   0.63  0.168
## 26  -34.30  +                  0.652       +        14 -766.053 1562.5   0.85  0.150
## 58 1099.00  +                  0.645       + -0.562 15 -765.207 1563.1   1.52  0.108
## 60  603.00  +     +            0.652       + -0.315 17 -763.010 1563.5   1.91  0.088
## 30  -31.61  +           -0.269 0.735       +        15 -765.553 1563.8   2.21  0.076
## 64  732.40  +     +     -0.379 0.768       + -0.377 18 -762.012 1564.0   2.35  0.071
## 62 1231.00  +           -0.317 0.741       + -0.626 16 -764.516 1564.1   2.51  0.065
## 20  -69.56  +     +                        +        15 -767.905 1568.5   6.91  0.007
## 18  -71.55  +                              +        13 -770.583 1569.2   7.58  0.005
## 27   -8.47        +            1.101       +        11 -772.895 1569.3   7.63  0.005
## 50 1137.00  +                              + -0.599 14 -769.667 1569.7   8.08  0.004
## 52  619.60  +     +                        + -0.341 16 -767.625 1570.4   8.73  0.003
## 24  -69.76  +     +     -0.028             +        16 -767.898 1570.9   9.28  0.002
## 59  568.60        +            1.087       + -0.286 12 -772.680 1571.1   9.48  0.002
## 44 1646.00  +     +            0.635         -0.829 11 -773.956 1571.4   9.75  0.002
## 22  -71.24  +            0.041             +        14 -770.570 1571.5   9.89  0.002
## 31   -8.68        +     -0.012  1.100      +        12 -772.892 1571.5   9.90  0.002
## 42 2092.00  +                  0.605         -1.054  9 -776.388 1571.8  10.14  0.001
## 54 1138.00  +           -0.002             + -0.599 15 -769.667 1572.1  10.43  0.001
## 25  -10.38                     1.106       +         9 -776.551 1572.1  10.47  0.001
## 12  -23.19  +     +            0.656                10 -775.675 1572.6  10.94  0.001
## 56  636.40  +     +     -0.047             + -0.350 17 -767.607 1572.7  11.11  0.001
## 57 1063.00                     1.080       + -0.532 10 -775.792 1572.8  11.17  0.001
## 48 1758.00  +     +     -0.201  0.696        -0.883 12 -773.676 1573.1  11.47  0.001
## 63  576.30        +     -0.019  1.086      + -0.290 13 -772.672 1573.4  11.76  0.001
## 46 2178.00  +           -0.136  0.646        -1.095 10 -776.262 1573.7  12.11  0.001
## 29  -10.04               0.019 1.107       +        10 -776.543 1574.3  12.68  0.000
## 16  -21.56  +     +     -0.116  0.692               11 -775.580 1574.6  13.00  0.000
## 61 1062.00               0.001 1.080       + -0.532 11 -775.792 1575.1  13.42  0.000
## 10  -28.63  +                  0.634                 8 -779.309 1575.4  13.78  0.000
## 34 2182.00  +                                -1.115  8 -780.041 1576.9  15.24  0.000
## 36 1742.00  +     +                          -0.894 10 -778.027 1577.3  15.64  0.000
## 14  -28.51  +           -0.008  0.636                9 -779.308 1577.6  15.98  0.000
## 38 2101.00  +            0.120                -1.075  9 -779.934 1578.9  17.23  0.000
## 4   -60.02  +     +                                  9 -779.947 1578.9  17.26  0.000
## 40 1694.00  +     +      0.079                -0.871 11 -777.980 1579.4  17.80  0.000
## Models ranked by AICc(x)
```

```
a <-get.models(my_model,subset=delta<2)
summary(model.avg(a))
## Call:
## model.avg(object = a)
## Component model call:
## lm(formula = d2H ~ <5 unique rhs>, data = Data, na.action = na.fail)
## Component models:
##       df  logLik    AICc delta weight
## 1245   16 -763.26 1561.63  0.00   0.31
## 12345 17 -762.37 1562.26  0.63   0.23
## 145   14 -766.05 1562.48  0.85   0.20
## 1456  15 -765.21 1563.14  1.52   0.14
## 12456 17 -763.01 1563.54  1.91   0.12
```

```
##
## Term codes:
##        Biome Feather_Type       Lat_GD      Long_GD   Sub_Family         Year
##            1            2            3            4            5            6
##
## Model-averaged coefficients:
## (full average)
##                          Estimate Std. Error Adjusted SE z value Pr(>|z|)
## (Intercept)             207.78972  631.70419   634.13951   0.328  0.74316
## BiomeCaatinga            15.09915    5.24438     5.27689   2.861  0.00422 **
## BiomeCerrado             -1.00581    4.13211     4.15365   0.242  0.80866
## BiomeMata_Atlantica       2.95922    5.35803     5.38903   0.549  0.58292
## BiomePampa               -7.12866    7.76006     7.80040   0.914  0.36078
## BiomePantanal             1.31369    5.72893     5.76086   0.228  0.81962
## Feather_TypeTail         -3.44889    3.96259     3.97886   0.867  0.38605
## Feather_TypeWing         -3.15275    2.91531     2.92286   1.079  0.28074
## Long_GD                   0.67772    0.23005     0.23157   2.927  0.00343 **
## Sub_FamilyDiglossinae    -7.19435    6.79582     6.84139   1.052  0.29299
## Sub_FamilyOthers          7.88649    7.67478     7.72576   1.021  0.30735
## Sub_FamilySaltatorinae   12.39028    8.76989     8.82935   1.403  0.16053
## Sub_FamilySporophilinae   5.53387    7.78434     7.83747   0.706  0.48014
## Sub_FamilyTachyphoninae  10.24915    6.61688     6.66076   1.539  0.12387
## Sub_FamilyThraupinae      4.93304    6.38565     6.42847   0.767  0.44286
## Lat_GD                   -0.08045    0.19938     0.19999   0.402  0.68750
## Year                     -0.11892    0.31364     0.31485   0.378  0.70565
##
## (conditional average)
##                         Estimate Std. Error Adjusted SE z value Pr(>|z|)
## (Intercept)             207.7897   631.7042    634.1395   0.328  0.74316
## BiomeCaatinga            15.0992     5.2444      5.2769   2.861  0.00422 **
## BiomeCerrado             -1.0058     4.1321      4.1537   0.242  0.80866
## BiomeMata_Atlantica       2.9592     5.3580      5.3890   0.549  0.58292
## BiomePampa               -7.1287     7.7601      7.8004   0.914  0.36078
## BiomePantanal             1.3137     5.7289      5.7609   0.228  0.81962
## Feather_TypeTail         -5.2788     3.7912      3.8172   1.383  0.16670
## Feather_TypeWing         -4.8256     2.2218      2.2369   2.157  0.03099 *
## Long_GD                   0.6777     0.2301      0.2316   2.927  0.00343 **
## Sub_FamilyDiglossinae    -7.1943     6.7958      6.8414   1.052  0.29299
## Sub_FamilyOthers          7.8865     7.6748      7.7258   1.021  0.30735
## Sub_FamilySaltatorinae   12.3903     8.7699      8.8293   1.403  0.16053
## Sub_FamilySporophilinae   5.5339     7.7843      7.8375   0.706  0.48014
## Sub_FamilyTachyphoninae  10.2491     6.6169      6.6608   1.539  0.12387
## Sub_FamilyThraupinae      4.9330     6.3856      6.4285   0.767  0.44286
## Lat_GD                   -0.3568     0.2788      0.2807   1.271  0.20372
## Year                     -0.4509     0.4726      0.4756   0.948  0.34304
## ---
## Signif. codes:  0 '***' 0.001 '**' 0.01 '*' 0.05 '.' 0.1 ' ' 1
```
