# Supplementary material for: A feather hydrogen (δ2H) isoscape for Brazil
Source: PLoS One. 2022 Aug 3;17(8):e0271573. doi: 10.1371/journal.pone.0271573 (PMC9348672; doi:10.1371/journal.pone.0271573)
Supplement: S2 File — R code to calculate WorldClim bioclimatic variables for a specific timeframe. (PDF) [file pone.0271573.s003.pdf]

S3 File. Bioclimatic variables calculation for a specific timeframe.
R code to calculate WorldClim bioclimatic variables for a specific timeframe.

1- 'Historical monthly weather data' for the timeframe of interest (2007-2019) were downloaded from Worldclim (https://www.worldclim.org/data/monthlywth.html)

2- The raster files for each month were cut based on a shape from Brazil

```
library(raster)
Tmin_jan10<-raster("wc2.1_2.5m_tmin_2010-01.tif")
Tmin_jan11<-raster("wc2.1_2.5m_tmin_2011-01.tif")
```

```
library(sf)
Area_of_interest<-st_read("Brazil_4326.shp")
Area_of_interest<-as(Area_of_interest,"Spatial
crs(Area_of_interest)<-NA
proj4string(Area_of_interest)<-CRS("+init=epsg:4326")
```

3- Raster of each month (from different years) were overlayed using mean values (only example) (function overlay() from package raster)

```
Jan_2007_2018<-overlay(Tmin_jan07, Tmin_jan08, Tmin_jan09, Tmin_jan10, Tmin_jan11, Tmin_jan12,
Tmin_jan13, Tmin_jan14, Tmin_jan15, Tmin_jan16, Tmin_jan17, Tmin_jan18, fun=mean)
proj4string (Jan_2007_2018)<- CRS("+init=epsg:4326")
Tmin_Jan_2007_2018<-mask(Jan_2007_2018,Area_of_interest)
```

4- All month/Year raster were stacked (only example) (function stack() from package raster)

```
Annual_Min_Temp_2007_2018 <- c(Tmin_Jan_2007_2018, Tmin_feb_2007_2018, Tmin_mar_2007_2018,
Tmin_april_2007_2018, Tmin_may_2007_2018, Tmin_jun_2007_2018,
Tmin_jul_2007_2018,Tmin_aug_2007_2018, Tmin_sep_2007_2018, Tmin_oct_2007_2018,
Tmin_nov_2007_2018, Tmin_dec_2007_2018)
Annual_Min_Temp_2007_2018 <-stack(Annual_Min_Temp_2007_2018)
```

5- Same procedure was run for maximum temperature and precipitation

6- Bioclimatic variables were calculated using 'dismo' package (only example)

```
library(dismo)

a<-biovars(Annual_Mean_Prec_2007_2018, Annual_Min_Temp_2007_2018,
Annual_Max_Temp_2007_2018)
as.matrix(a)
```
