# Supplementary material for: A feather hydrogen (δ2H) isoscape for Brazil
Source: PLoS One. 2022 Aug 3;17(8):e0271573. doi: 10.1371/journal.pone.0271573 (PMC9348672; doi:10.1371/journal.pone.0271573)
Supplement: S3 File — R code and results. Including S4 Fig (Linear regressions between δ2Hf and δ2Hp) and S4 Table (Precipitation isotopic values extracted from waterisotopes.org). (PDF) [file pone.0271573.s004.pdf]

S4 File. Defining a better timeframe for Isotopic precipitation values in Brazil.

R code and results. Including S4 Figure (Linear regressions between δ^2H_f and δ^2H_p, showing the regression line and each associated r^2. Associated maps correspond to waterisotopes.org data for Brazil and lines within the maps represent Brazilian biomes) and S4 Table (Precipitation isotopic values extracted from waterisotopes.org for the same coordinates as our samples).

1- Raster of monthly isotopic precipitation were downloaded from (https://wateriso.utah.edu/waterisotopes/index.html), as well as annual and growing season values

2- The raster were cropped to Brazil (functions crop() and mask() from package raster)

3- Isotopic precipitation values were extracted for each month, using our tissue sampling points (function extract() from package raster)

4- Linear regressions were run for each month, between δ^2H_f and δ^2H_p

```
RcodeBowen<-read.table("Prec_Isotope_Bowen.txt",h=T)
attach(Bowen)
```

```
summary(lm(X.²Hf~X.²Hp_jan))
```

January	Estimate	SE	t value	p value
Intercept	-46.467	2.44	-18.96	<0.001
δ^2H_p jan	0.522	0.09	5.66	<0.001

Residual standard error: 16.76 on 190 degrees of freedom
Multiple R-squared: 0.1445, Adjusted R-squared: 0.14
F-statistic: 32.08 on 1 and 190 DF, p-value: 5.4e-08

```
summary(lm(X.²Hf~X.²Hp_feb))
```

February	Estimate	SE	t value	p value
Intercept	-44.588	2.20	-20.19	<0.001
δ^2H_p feb	0.514	0.06	7.40	<0.001

Residual standard error: 15.96 on 190 degrees of freedom
Multiple R-squared: 0.224, Adjusted R-squared: 0.2199
F-statistic: 54.84 on 1 and 190 DF, p-value: 4.134e-12

```
summary(lm(X.²Hf~X.²Hp_mar))
```

March	Estimate	SE	t value	p value
Intercept	-41.598	2.44	-16.99	<0.001
δ^2H_p mar	0.538	0.06	7.81	<0.001

Residual standard error: 15.77 on 190 degrees of freedom
Multiple R-squared: 0.243, Adjusted R-squared: 0.239
F-statistic: 60.99 on 1 and 190 DF, p-value: 3.774e-13

```
summary(lm(X.²Hf~X.²Hp_apr))
```

April	Estimate	SE	t value	p value
Intercept	-33.590	3.26	-10.27	<0.001
δ^2H_p apr	0.758	0.09	8.12	<0.001

Residual standard error: 15.61 on 190 degrees of freedom
Multiple R-squared: 0.258, Adjusted R-squared: 0.2541
F-statistic: 66.05 on 1 and 190 DF, p-value: 5.495e-14

```
summary(lm(X.²Hf~X.²Hp_may))
```

May	Estimate	SE	t value	p value
Intercept	-48.029	2.32	-20.64	<0.001
δ^2H_p may	0.463	0.08	5.30	<0.001

Residual standard error: 16.91 on 190 degrees of freedom
Multiple R-squared: 0.129, Adjusted R-squared: 0.1244
F-statistic: 28.13 on 1 and 190 DF, p-value: 3.129e-07

```
summary(lm(X.²Hf~X.²Hp_jun))
```

June	Estimate	SE	t value	p value
Intercept	-51.766	2.09	-24.73	<0.001
δ^2H_p jun	0.569	0.14	4.04	<0.001

Residual standard error: 17.39 on 190 degrees of freedom
Multiple R-squared: 0.07916, Adjusted R-squared:0.07432
F-statistic: 16.33 on 1 and 190 DF, p-value: 7.706e-05

```
summary(lm(X.²Hf~X.²Hp_jul))
```

July	Estimate	SE	t value	p value
Intercept	-54.881	1.74	-31.45	<0.001
δ^2H_p jul	0.576	0.18	3.07	0.002

Residual standard error: 17.69 on 190 degrees of freedom
Multiple R-squared: 0.04725, Adjusted R-squared:0.04223
F-statistic: 9.422 on 1 and 190 DF, p-value: 0.002458

```
summary(lm(X.²Hf~X.²Hp_aug))
```

August	Estimate	SE	t value	p value
Intercept	-58.578	1.30	-44.78	<0.001
δ^2H_p aug	-0.102	0.15	-0.65	0.515

Residual standard error: 18.1 on 190 degrees of freedom
Multiple R-squared: 0.00223, Adjusted R-squared: -0.0030
F-statistic: 0.4246 on 1 and 190 DF, p-value: 0.5154

```
summary(lm(X.²Hf~X.²Hp_sep))
```

September	Estimate	SE	t value	p value
Intercept	-58.095	1.28	-45.27	<0.001
δ^2H_p sep	-0.659	0.21	-3.11	0.002

Residual standard error: 17.67 on 190 degrees of freedom
Multiple R-squared: 0.04856, Adjusted R-squared:0.04356
F-statistic: 9.698 on 1 and 190 DF, p-value: 0.00213

```
summary(lm(X.²Hf~X.²Hp_oct))
```

October	Estimate	SE	t value	p value
Intercept	-55.295	1.61	-34.31	<0.001
δ^2H_p oct	0.602	0.18	3.27	0.001

Residual standard error: 17.63 on 190 degrees of freedom
Multiple R-squared: 0.05348, Adjusted R-squared: 0.0485
F-statistic: 10.73 on 1 and 190 DF, p-value: 0.00125

```
summary(lm(X.²Hf~X.²Hp_nov))
```

November	Estimate	SE	t value	p value
Intercept	-49.111	2.27	-21.63	<0.001
δ^2H_p nov	0.570	0.11	4.94	<0.001

Residual standard error: 17.06 on 190 degrees of freedom
Multiple R-squared: 0.1138, Adjusted R-squared: 0.1092
F-statistic: 24.41 on 1 and 190 DF, p-value: 1.705e-06

```
summary(lm(X.²Hf~X.²Hp_dec))
```

December	Estimate	SE	t value	p value
Intercept	-49.195	2.13	-23.08	<0.001
δ^2H_p dec	0.444	0.08	5.34	<0.001

Residual standard error: 16.89 on 190 degrees of freedom
Multiple R-squared: 0.1307, Adjusted R-squared: 0.1261
F-statistic: 28.56 on 1 and 190 DF, p-value: 2.59e-07

5- We also run linear regressions for Annual and Growing season values of δ^2H_p, in order to compar e the values (S3 Figure)

```
summary(lm(X.²Hf~X.²Hp_Annual))
```

Mean Annual	Estimate	SE	t value	p value
Intercept	-43.646	2.65	-16.96	<0.001
δ^2H_p annual	0.635	0.10	6.28	<0.001

Residual standard error: 16.49 on 190 degrees of freedom
Multiple R-squared: 0.1722, Adjusted R-squared: 0.1678
F-statistic: 39.51 on 1 and 190 DF, p-value: 2.18e-09

```
summary(lm(X.²Hf~X.²Hp_GS))
```

Growing season	Estimate	SE	t value	p value
Intercept	-42.034	2.64	-15.87	<0.001
δ^2H_p GS	0.734	0.10	6.94	<0.001

Residual standard error: 16.18 on 190 degrees of freedom
Multiple R-squared: 0.2024, Adjusted R-squared: 0.1982
F-statistic: 48.22 on 1 and 190 DF, p-value: 5.887e-11

6- Monthly isoscapes were overlayed by means, using different consecutive-months combinations (functions overlay() from package raster)

7- Isotopic precipitation values were extracted for each month combination, using our tissue sampling points (function extract() from package raster)

8- New linear regressions were run for each month combination, between δ^2H_f and δ^2H_p

9- The combination Feb+Mar+April was chosen as the best (S3 Figure)

```
summary(lm(X.²Hf~X.²Hp_Nov_May))
```

November-May

	Estimate	SE	t value	p value
Intercept	-41.614	2.58	-16.11	<0.001
δ^2H_p nov-may	0.677	0.09	7.32	<0.001

Residual standard error: 16 on 190 degrees of freedom
Multiple R-squared: 0.2203, Adjusted R-squared: 0.2162
F-statistic: 53.68 on 1 and 190 DF, p-value: 6.55e-12

```
summary(lm(X.²Hf~X.²Hp_Jan_April))
```

January-April

	Estimate	SE	t value	p value
Intercept	-40.492	2.61	-15.49	<0.001
δ^2H_p jan-apr	0.630	0.08	7.67	<0.001

Residual standard error: 15.83 on 190 degrees of freedom
Multiple R-squared: 0.2368, Adjusted R-squared: 0.2328
F-statistic: 58.95 on 1 and 190 DF, p-value: 8.295e-13

```
summary(lm(X.²Hf~X.²Hp_Dec_May))
```

December-May

	Estimate	SE	t value	p value
Intercept	-40.937	2.59	-15.76	<0.001
δ^2H_p dec-may	0.667	0.08	7.55	<0.001

Residual standard error: 15.89 on 190 degrees of freedom
Multiple R-squared: 0.2308, Adjusted R-squared: 0.2268
F-statistic: 57.02 on 1 and 190 DF, p-value: 1.758e-12

```
summary(lm(X.²Hf~X.²Hp_Feb_April))
```

February-April

	Estimate	SE	t value	p value
Intercept	-39.437	2.61	-15.07	<0.001
δ^2H_p feb-apr	0.626	0.07	8.09	<0.001

Residual standard error: 15.63 on 190 degrees of freedom
Multiple R-squared: 0.2562, Adjusted R-squared: 0.2523
F-statistic: 65.44 on 1 and 190 DF, p-value: 6.924e-14

```
summary(lm(X.²Hf~X.²Hp_Dec_April))
```

December-April

	Estimate	SE	t value	p value
Intercept	-41.641	2.55	-16.29	<0.001
δ^2H_p dec-apr	0.623	0.08	7.40	<0.001

Residual standard error: 15.96 on 190 degrees of freedom
Multiple R-squared: 0.224, Adjusted R-squared: 0.2199
F-statistic: 54.84 on 1 and 190 DF, p-value: 4.147e-12

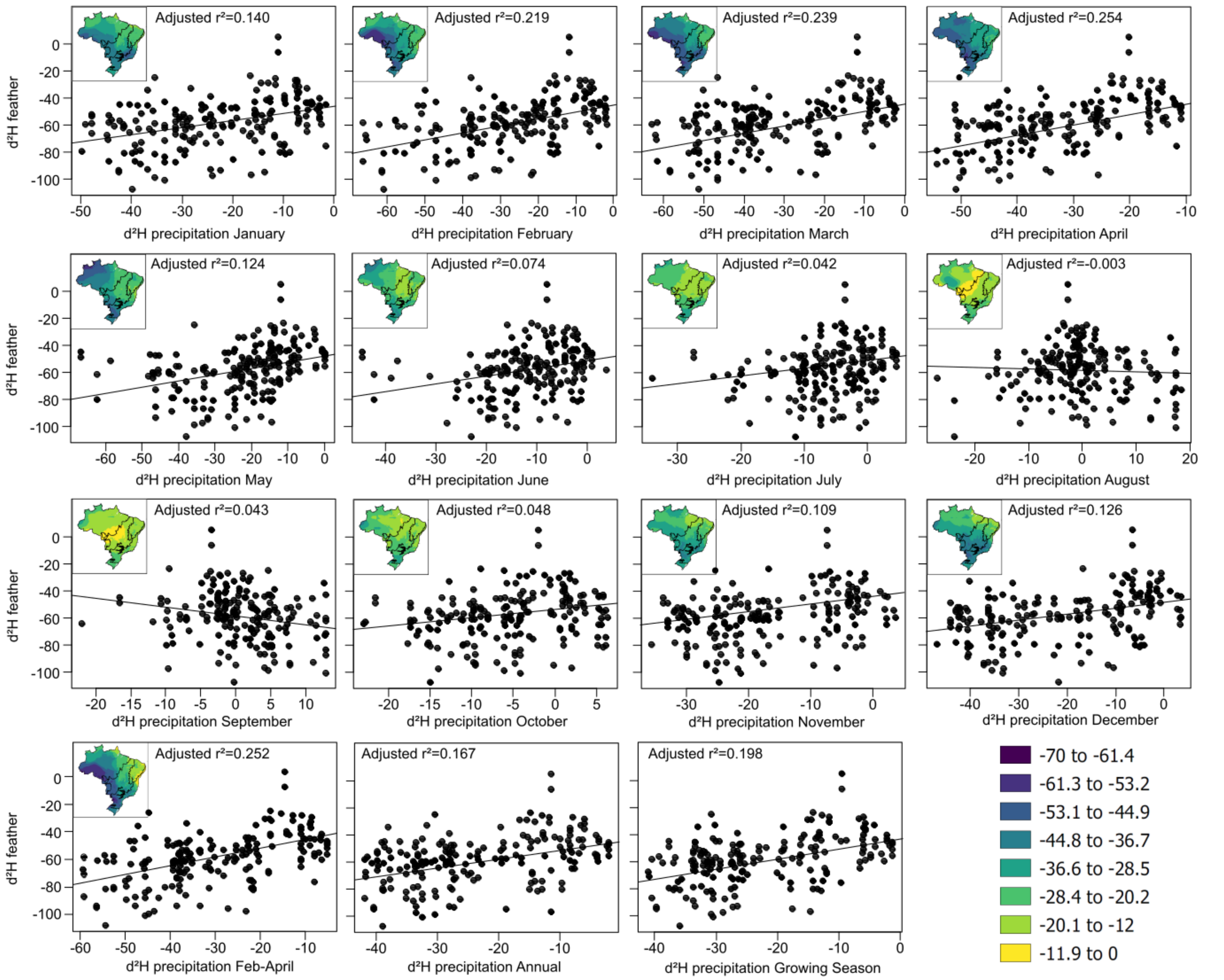

S4 Figure. Linear regressions between δ^2H_f and δ^2H_p, showing the regression line and each associated r^2. Associated maps correspond to waterisotopes.org data for Brazil and lines within the maps represent Brazilian biomes.

S4 Table. Precipitation isotopic values extracted from waterisotopes.org for the same coordinates as our samples.

		δ²Hp (‰)																	
**ID**	**δ²Hf (‰)**	**Jan**	**Feb**	**Mar**	**Apr**	**May**	**Jun**	**Jul**	**Aug**	**Sep**	**Oct**	**Nov**	**Dec**	**Nov-May**	**Dec-May**	**Dec-April**	**Jan-April**	**Feb-April**	**Annual**	**GS**
RDA001	-54.86	-19.32	-19.09	-17.58	-25.85	-13.82	-12.54	-5.61	-4.31	-9.76	-9.84	-18.51	-18.05	-18.89	-18.95	-19.98	-20.46	-20.84	-17.11	-16.37
RDA002	-47.86	-19.32	-19.09	-17.58	-25.85	-13.82	-12.54	-5.61	-4.31	-9.76	-9.84	-18.51	-18.05	-18.89	-18.95	-19.98	-20.46	-20.84	-17.11	-16.37
RDA003	-60.99	-23.92	-12.91	-24.08	-24.03	-9.63	-7.69	-2.90	-2.12	-2.64	-5.40	-18.46	-23.71	-19.53	-19.71	-21.73	-21.24	-20.34	-16.32	-16.51
RDA004	-56.65	-23.92	-12.91	-24.08	-24.03	-9.63	-7.69	-2.90	-2.12	-2.64	-5.40	-18.46	-23.71	-19.53	-19.71	-21.73	-21.24	-20.34	-16.32	-16.51
RDA005	-45.06	-40.47	-18.86	-44.97	-30.10	-12.38	-7.18	-5.36	-4.01	1.27	-6.00	-25.66	-36.21	-29.81	-30.50	-34.12	-33.60	-31.31	-36.16	-29.47
RDA006	-65.72	-40.47	-18.86	-44.97	-30.10	-12.38	-7.18	-5.36	-4.01	1.27	-6.00	-25.66	-36.21	-29.81	-30.50	-34.12	-33.60	-31.31	-36.16	-29.47
RDA007	-45.51	-21.94	-20.64	-19.79	-28.07	-15.32	-14.32	-6.65	-5.53	-10.67	-12.29	-22.15	-22.84	-21.54	-21.43	-22.66	-22.61	-22.83	-19.17	-18.97
RDA008	-49.85	-21.94	-20.64	-19.79	-28.07	-15.32	-14.32	-6.65	-5.53	-10.67	-12.29	-22.15	-22.84	-21.54	-21.43	-22.66	-22.61	-22.83	-19.17	-18.97
RDA009	-28.06	-7.34	-5.74	-5.82	-14.36	-2.89	-2.55	2.19	2.45	-2.38	-1.23	-7.63	-8.10	-7.41	-7.37	-8.27	-8.32	-8.64	-6.00	-6.72
RDA010	-23.88	-16.62	-15.66	-14.19	-23.33	-11.37	-11.16	-4.11	-3.50	-9.57	-8.94	-16.88	-16.79	-16.41	-16.33	-17.32	-17.45	-17.73	-14.84	-14.33
RDA011	-86.81	-39.37	-41.69	-49.20	-52.62	-35.87	-18.37	-8.59	2.05	3.41	-9.64	-30.01	-40.38	-41.31	-43.19	-44.65	-45.72	-47.84	-35.17	-34.90
RDA012	-92.99	-36.86	-50.26	-49.41	-44.10	-26.36	-16.24	-3.89	17.26	10.98	-7.00	-23.68	-34.94	-37.95	-40.32	-43.12	-45.16	-47.93	-33.56	-33.53
RDA013	-62.21	-33.53	-47.00	-46.47	-43.34	-24.75	-14.17	-1.38	17.42	12.80	-4.24	-21.41	-33.47	-35.71	-38.09	-40.76	-42.59	-45.61	-32.58	-30.65
RDA014	-71.35	-15.70	-24.08	-29.63	-30.94	-19.49	-6.57	-2.02	2.94	2.55	2.55	-7.10	-9.80	-19.54	-21.61	-22.03	-25.09	-28.22	-23.33	-19.46
RDA015	-52.52	-15.70	-24.08	-29.63	-30.94	-19.49	-6.57	-2.02	2.94	2.55	2.55	-7.10	-9.80	-19.54	-21.61	-22.03	-25.09	-28.22	-23.33	-19.46
RDA016	-67.39	-44.92	-65.63	-62.12	-50.68	-33.06	-19.86	-8.25	8.08	7.06	-10.15	-26.09	-35.19	-45.38	-48.60	-51.71	-55.84	-59.48	-41.85	-41.08
RDA017	-86.91	-44.61	-66.38	-61.67	-50.14	-33.35	-18.78	-10.14	-10.64	4.13	-12.51	-24.47	-30.16	-44.40	-47.72	-50.59	-55.70	-59.39	-41.95	-40.52
RDA018	-68.04	-16.75	-25.46	-31.09	-31.57	-20.28	-7.20	-2.46	2.26	2.15	2.00	-7.86	-10.19	-20.46	-22.56	-23.01	-26.22	-29.37	-24.50	-20.08
RDA019	-70.56	-44.74	-65.35	-61.92	-50.65	-32.96	-19.91	-8.10	9.06	7.22	-10.01	-26.21	-35.56	-45.34	-48.53	-51.65	-55.67	-59.31	-41.81	-40.99
RDA020	-94.68	-42.65	-61.45	-58.48	-49.69	-32.04	-19.72	-7.45	12.71	7.72	-9.55	-26.91	-37.36	-44.08	-46.95	-49.93	-53.07	-56.54	-40.49	-39.72
RDA021	-57.88	-44.61	-66.38	-61.67	-50.14	-33.35	-18.78	-10.14	-10.64	4.13	-12.51	-24.47	-30.16	-44.40	-47.72	-50.59	-55.70	-59.39	-41.95	-40.52
RDA022	-45.64	-36.93	-50.36	-49.49	-44.13	-26.41	-16.30	-3.96	17.20	10.93	-7.07	-23.71	-34.98	-38.00	-40.38	-43.18	-45.23	-47.99	-33.50	-33.47
RDA023	-74.92	-39.40	-53.07	-52.11	-45.27	-26.54	-15.63	-3.39	16.82	11.53	-6.98	-25.57	-35.74	-39.67	-42.02	-45.12	-47.46	-50.15	-38.86	-36.31
RDA024	-93.21	-42.65	-61.45	-58.48	-49.69	-32.04	-19.72	-7.45	12.71	7.72	-9.55	-26.91	-37.36	-44.08	-46.95	-49.93	-53.07	-56.54	-40.49	-39.72
RDA025	-61.44	-15.13	-23.44	-28.99	-30.88	-19.54	-6.76	-2.29	2.60	2.44	2.43	-7.17	-10.50	-19.38	-21.41	-21.79	-24.61	-27.77	-22.76	-19.08
RDA026	-79.23	-39.28	-57.77	-52.38	-47.89	-33.38	-19.21	-8.60	-8.09	4.04	-11.03	-23.80	-26.82	-40.19	-42.92	-44.83	-49.33	-52.68	-37.65	-36.76
RDA027	-57.03	-39.28	-57.77	-52.38	-47.89	-33.38	-19.21	-8.60	-8.09	4.04	-11.03	-23.80	-26.82	-40.19	-42.92	-44.83	-49.33	-52.68	-37.65	-36.76
RDA028	-60.36	-33.53	-47.00	-46.47	-43.34	-24.75	-14.17	-1.38	17.42	12.80	-4.24	-21.41	-33.47	-35.71	-38.09	-40.76	-42.59	-45.61	-32.58	-30.65
RDA029	-40.53	-2.84	-2.37	-4.08	-12.44	-4.32	-0.57	2.18	1.60	1.49	4.83	1.21	1.33	-3.36	-4.12	-4.08	-5.43	-6.30	-3.97	-2.31
RDA030	-57.32	-31.40	-25.78	-34.83	-30.39	-23.21	-13.20	-11.85	-3.72	-0.38	-8.09	-19.50	-27.31	-27.49	-28.82	-29.94	-30.60	-30.33	-20.58	-22.86

		δ^2H_p (‰)																		
**ID**	**δ^2H_f (‰)**	**Jan**	**Feb**	**Mar**	**Apr**	**May**	**Jun**	**Jul**	**Aug**	**Sep**	**Oct**	**Nov**	**Dec**	**Nov-May**	**Dec-May**	**Dec-April**	**Jan-April**	**Feb-April**	**Annual**	**GS**
RDA031	-49.09	-5.30	-5.24	-6.81	-15.15	-7.20	-2.93	0.11	-0.52	0.36	3.07	-0.92	-0.73	-5.91	-6.74	-6.65	-8.12	-9.07	-6.31	-4.21
RDA032	-37.13	-7.25	-7.38	-8.46	-16.92	-8.65	-4.77	-1.03	-1.57	-1.25	1.11	-3.39	-2.97	-7.86	-8.61	-8.60	-10.00	-10.92	-8.27	-5.78
RDA033	-33.16	-5.33	-5.28	-6.59	-14.92	-6.49	-2.86	0.57	0.06	-0.34	2.46	-1.85	-1.41	-5.98	-6.67	-6.71	-8.03	-8.93	-6.51	-4.27
RDA034	-58.83	-7.25	-7.38	-8.46	-16.92	-8.65	-4.77	-1.03	-1.57	-1.25	1.11	-3.39	-2.97	-7.86	-8.61	-8.60	-10.00	-10.92	-8.27	-5.78
RDA035	-43.43	-5.30	-5.24	-6.81	-15.15	-7.20	-2.93	0.11	-0.52	0.36	3.07	-0.92	-0.73	-5.91	-6.74	-6.65	-8.12	-9.07	-6.31	-4.21
RDA036	-6.05	-11.14	-11.79	-11.96	-20.17	-12.00	-8.05	-3.58	-2.70	-3.53	-1.97	-7.35	-6.50	-11.56	-12.26	-12.31	-13.77	-14.64	-11.47	-9.59
RDA037	-47.53	-7.43	-7.76	-8.62	-16.69	-8.33	-4.49	-0.78	-0.60	-1.39	0.92	-3.88	-3.12	-7.98	-8.66	-8.73	-10.13	-11.03	-8.25	-6.03
RDA038	-35.15	-7.43	-7.76	-8.62	-16.69	-8.33	-4.49	-0.78	-0.60	-1.39	0.92	-3.88	-3.12	-7.98	-8.66	-8.73	-10.13	-11.03	-8.25	-6.03
RDA039	-50.82	-7.43	-7.76	-8.62	-16.69	-8.33	-4.49	-0.78	-0.60	-1.39	0.92	-3.88	-3.12	-7.98	-8.66	-8.73	-10.13	-11.03	-8.25	-6.03
RDA040	5.01	-11.14	-11.79	-11.96	-20.17	-12.00	-8.05	-3.58	-2.70	-3.53	-1.97	-7.35	-6.50	-11.56	-12.26	-12.31	-13.77	-14.64	-11.47	-9.59
RDA041	-59.23	-2.58	-1.70	-4.35	-13.28	-6.95	-1.15	0.84	0.92	3.05	6.36	3.68	3.54	-3.09	-4.22	-3.68	-5.48	-6.45	-3.75	-2.69
RDA042	-54.07	-3.00	-2.13	-4.83	-13.74	-7.46	-1.52	0.53	0.69	2.91	6.14	3.40	3.31	-3.49	-4.64	-4.08	-5.93	-6.90	-4.14	-3.09
RDA043	-40.36	-12.66	-14.93	-15.64	-23.05	-18.95	-12.57	-7.93	-4.84	-3.63	-3.82	-7.28	-6.60	-14.16	-15.31	-14.58	-16.57	-17.87	-15.93	-15.76
RDA044	-49.38	-12.39	-14.63	-15.17	-22.65	-18.24	-12.21	-7.55	-4.58	-3.74	-3.84	-7.38	-6.75	-13.89	-14.97	-14.32	-16.21	-17.48	-14.77	-14.62
RDA045	-29.10	-13.11	-15.19	-15.45	-23.10	-18.13	-12.42	-7.37	-4.57	-4.15	-4.32	-8.40	-7.67	-14.44	-15.44	-14.90	-16.71	-17.91	-15.37	-15.13
RDA046	-25.30	-11.70	-12.26	-12.83	-21.13	-14.73	-9.89	-5.14	-3.40	-3.61	-2.68	-7.01	-6.29	-12.28	-13.16	-12.84	-14.48	-15.41	-12.90	-12.74
RDA047	-41.30	-9.84	-10.56	-11.16	-19.14	-12.22	-7.81	-3.40	-1.68	-2.48	-1.31	-5.69	-4.96	-10.51	-11.31	-11.13	-12.68	-13.62	-10.82	-10.58
RDA048	-43.49	-5.52	-5.45	-8.92	-16.59	-9.83	-2.81	-0.82	0.22	2.87	5.32	1.92	2.77	-5.95	-7.26	-6.74	-9.12	-10.32	-6.84	-6.14
RDA049	-60.13	-5.40	-6.31	-9.83	-16.53	-9.95	-2.64	-0.83	0.84	3.38	5.56	2.11	3.20	-6.10	-7.47	-6.97	-9.52	-10.89	-7.52	-6.67
RDA050	-64.86	-5.40	-6.31	-9.83	-16.53	-9.95	-2.64	-0.83	0.84	3.38	5.56	2.11	3.20	-6.10	-7.47	-6.97	-9.52	-10.89	-7.52	-6.67
RDA051	-44.57	-4.36	-5.96	-8.77	-15.14	-10.70	-3.38	-0.72	2.36	4.15	5.20	2.35	3.51	-5.58	-6.90	-6.14	-8.56	-9.96	-8.66	-8.88
RDA052	-53.80	-5.52	-5.45	-8.92	-16.59	-9.83	-2.81	-0.82	0.22	2.87	5.32	1.92	2.77	-5.95	-7.26	-6.74	-9.12	-10.32	-6.84	-6.14
RDA053	-34.22	-15.34	-18.31	-18.94	-25.76	-22.09	-15.12	-10.05	-6.35	-4.88	-5.78	-9.64	-8.68	-16.96	-18.18	-17.40	-19.58	-21.00	-19.68	-19.17
RDA054	-51.71	-15.34	-18.31	-18.94	-25.76	-22.09	-15.12	-10.05	-6.35	-4.88	-5.78	-9.64	-8.68	-16.96	-18.18	-17.40	-19.58	-21.00	-19.68	-19.17
RDA055	-40.36	-9.86	-10.17	-12.39	-19.91	-14.71	-7.71	-3.89	-1.35	0.43	1.17	-2.60	-1.48	-10.16	-11.42	-10.76	-13.08	-14.16	-12.72	-13.18
RDA056	-38.47	-9.86	-10.17	-12.39	-19.91	-14.71	-7.71	-3.89	-1.35	0.43	1.17	-2.60	-1.48	-10.16	-11.42	-10.76	-13.08	-14.16	-12.72	-13.18
RDA057	-26.61	-7.70	-9.05	-11.01	-18.36	-13.77	-6.94	-3.54	-1.12	0.98	1.82	-1.24	-0.55	-8.81	-10.08	-9.34	-11.53	-12.81	-11.70	-11.61
RDA058	-30.96	-7.70	-9.05	-11.01	-18.36	-13.77	-6.94	-3.54	-1.12	0.98	1.82	-1.24	-0.55	-8.81	-10.08	-9.34	-11.53	-12.81	-11.70	-11.61
RDA059	-55.52	-30.60	-38.35	-38.84	-37.62	-21.46	-10.40	-10.23	-3.82	-0.41	-15.31	-32.03	-43.14	-34.58	-35.00	-37.71	-36.35	-38.27	-33.32	-32.53
RDA060	-52.06	-31.28	-15.01	-35.60	-25.81	-9.10	-4.45	-2.53	-2.33	2.11	-3.70	-20.25	-28.95	-23.72	-24.29	-27.33	-26.93	-25.48	-28.69	-23.01
RDA061	-32.85	-28.41	-35.66	-37.41	-35.97	-19.38	-8.35	-7.39	-2.69	1.73	-12.70	-28.93	-40.18	-32.28	-32.84	-35.53	-34.36	-36.35	-31.83	-30.20

		δ^2H_p (‰)																		
**ID**	**δ^2H_f (‰)**	**Jan**	**Feb**	**Mar**	**Apr**	**May**	**Jun**	**Jul**	**Aug**	**Sep**	**Oct**	**Nov**	**Dec**	**Nov-May**	**Dec-May**	**Dec-April**	**Jan-April**	**Feb-April**	**Annual**	**GS**
RDA062	-49.18	-31.28	-15.01	-35.60	-25.81	-9.10	-4.45	-2.53	-2.33	2.11	-3.70	-20.25	-28.95	-23.72	-24.29	-27.33	-26.93	-25.48	-28.69	-23.01
RDA063	-67.04	-26.50	-34.31	-41.73	-38.62	-37.91	-25.99	-20.68	-12.30	-9.41	-15.26	-23.08	-29.03	-33.03	-34.68	-34.04	-35.29	-38.22	-25.70	-25.74
RDA064	-30.43	-11.58	-15.42	-15.79	-21.39	-11.12	-4.77	-1.65	0.03	2.06	0.52	-5.70	-7.51	-12.64	-13.80	-14.34	-16.04	-17.53	-14.08	-11.70
RDA065	-67.63	-11.87	-16.10	-16.48	-22.02	-12.28	-5.40	-2.30	-0.33	2.14	0.61	-5.78	-7.40	-13.13	-14.36	-14.78	-16.62	-18.20	-14.46	-12.12
RDA066	-68.83	-11.87	-16.10	-16.48	-22.02	-12.28	-5.40	-2.30	-0.33	2.14	0.61	-5.78	-7.40	-13.13	-14.36	-14.78	-16.62	-18.20	-14.46	-12.12
RDA067	-58.46	-16.98	-24.40	-27.23	-24.69	-7.44	3.73	1.24	4.21	11.50	-4.12	-17.27	-28.42	-20.92	-21.53	-24.34	-23.32	-25.44	-23.33	-19.81
RDA068	-67.46	-23.30	-29.40	-32.74	-30.80	-13.80	-2.96	-2.99	0.09	6.15	-9.07	-23.32	-34.52	-26.84	-27.43	-30.15	-29.06	-30.98	-27.69	-25.86
RDA069	-53.00	-44.46	-19.92	-49.27	-31.83	-13.99	-8.14	-7.23	-5.07	2.36	-6.11	-27.55	-40.94	-32.56	-33.40	-37.28	-36.37	-33.67	-36.07	-31.91
RDA070	-59.70	-49.12	-62.07	-63.01	-43.71	-30.23	-6.95	-5.47	1.09	7.98	-5.80	-15.18	-19.68	-40.43	-44.64	-47.52	-54.48	-56.27	-34.04	-34.12
RDA071	-59.81	-29.98	-37.92	-38.36	-36.95	-20.79	-9.65	-9.81	-3.22	0.12	-14.98	-31.41	-42.57	-34.00	-34.43	-37.16	-35.80	-37.74	-32.59	-31.97
RDA072	-51.17	-2.95	-2.18	-2.59	-10.89	0.09	0.78	4.55	4.17	-2.61	1.79	-2.44	-2.72	-3.38	-3.54	-4.27	-4.65	-5.22	-1.91	-1.32
RDA073	-46.81	-2.95	-2.18	-2.59	-10.89	0.09	0.78	4.55	4.17	-2.61	1.79	-2.44	-2.72	-3.38	-3.54	-4.27	-4.65	-5.22	-1.91	-1.32
RDA074	-68.31	-29.98	-37.92	-38.36	-36.95	-20.79	-9.65	-9.81	-3.22	0.12	-14.98	-31.41	-42.57	-34.00	-34.43	-37.16	-35.80	-37.74	-32.59	-31.97
RDA075	-44.95	-42.38	-29.49	-42.31	-35.66	-24.82	-17.87	-15.72	-7.17	-4.76	-13.55	-29.06	-36.29	-34.29	-35.16	-37.23	-37.46	-35.82	-29.30	-30.11
RDA076	-56.84	-42.38	-29.49	-42.31	-35.66	-24.82	-17.87	-15.72	-7.17	-4.76	-13.55	-29.06	-36.29	-34.29	-35.16	-37.23	-37.46	-35.82	-29.30	-30.11
RDA077	-61.95	-41.76	-28.59	-41.83	-35.05	-24.00	-17.16	-15.20	-6.72	-4.19	-12.89	-28.39	-35.75	-33.63	-34.50	-36.60	-36.81	-35.16	-28.70	-29.42
RDA078	-59.68	-41.76	-28.59	-41.83	-35.05	-24.00	-17.16	-15.20	-6.72	-4.19	-12.89	-28.39	-35.75	-33.63	-34.50	-36.60	-36.81	-35.16	-28.70	-29.42
RDA079	-73.63	-25.25	-34.57	-37.65	-41.24	-40.16	-16.23	-7.86	-1.00	0.03	-3.08	-14.95	-15.47	-29.90	-32.39	-30.84	-34.68	-37.82	-24.07	-25.71
RDA080	-47.15	-25.51	-34.64	-38.01	-41.47	-40.61	-16.46	-8.08	-1.02	-0.26	-3.22	-15.23	-15.66	-30.16	-32.65	-31.06	-34.91	-38.04	-24.69	-26.09
RDA081	-55.25	-25.51	-34.64	-38.01	-41.47	-40.61	-16.46	-8.08	-1.02	-0.26	-3.22	-15.23	-15.66	-30.16	-32.65	-31.06	-34.91	-38.04	-24.69	-26.09
RDA082	-55.80	-25.49	-35.29	-37.58	-41.47	-39.83	-16.40	-7.86	-1.46	0.21	-3.43	-15.15	-15.75	-30.08	-32.57	-31.12	-34.96	-38.11	-23.45	-25.65
RDA083	-29.18	-14.40	-17.01	-16.54	-23.36	-13.86	-8.61	-4.05	-2.13	-2.20	-3.47	-9.91	-10.62	-15.10	-15.96	-16.39	-17.83	-18.97	-16.50	-14.56
RDA084	-41.62	-13.43	-15.79	-15.62	-22.20	-12.55	-7.35	-3.04	-1.12	-1.53	-2.68	-9.14	-9.94	-14.09	-14.92	-15.39	-16.76	-17.87	-15.24	-13.59
RDA085	-42.85	-12.72	-18.59	-24.06	-26.96	-14.71	-2.28	1.01	8.17	4.67	5.75	-1.49	-5.70	-14.89	-17.12	-17.61	-20.58	-23.20	-12.44	-16.14
RDA086	-55.44	-12.72	-18.59	-24.06	-26.96	-14.71	-2.28	1.01	8.17	4.67	5.75	-1.49	-5.70	-14.89	-17.12	-17.61	-20.58	-23.20	-12.44	-16.14
RDA087	-79.98	-14.23	-15.08	-35.88	-43.51	-62.37	-42.32	-22.03	-11.58	-9.94	-11.07	-16.86	-17.68	-29.37	-31.46	-25.28	-27.18	-31.49	-27.60	-27.56
RDA088	-61.46	-14.23	-15.08	-35.88	-43.51	-62.37	-42.32	-22.03	-11.58	-9.94	-11.07	-16.86	-17.68	-29.37	-31.46	-25.28	-27.18	-31.49	-27.60	-27.56
RDA089	-64.55	-14.35	-20.77	-21.32	-25.27	-12.52	-0.70	2.39	8.50	7.37	5.29	-2.85	-8.92	-15.14	-17.19	-18.13	-20.43	-22.46	-7.95	-12.37
RDA090	-52.98	-14.35	-20.77	-21.32	-25.27	-12.52	-0.70	2.39	8.50	7.37	5.29	-2.85	-8.92	-15.14	-17.19	-18.13	-20.43	-22.46	-7.95	-12.37
RDA091	-45.81	-38.85	-51.85	-51.63	-43.64	-27.34	-14.01	-4.30	11.46	8.75	-6.58	-23.96	-31.18	-38.35	-40.75	-43.43	-46.49	-49.04	-39.13	-33.51
RDA092	-51.31	-38.05	-51.00	-50.89	-42.99	-26.85	-13.38	-3.91	11.54	9.04	-5.96	-23.11	-30.27	-37.59	-40.01	-42.64	-45.73	-48.29	-38.04	-32.77

		δ^2H_p (‰)																		
**ID**	**δ^2H_f (‰)**	**Jan**	**Feb**	**Mar**	**Apr**	**May**	**Jun**	**Jul**	**Aug**	**Sep**	**Oct**	**Nov**	**Dec**	**Nov-May**	**Dec-May**	**Dec-April**	**Jan-April**	**Feb-April**	**Annual**	**GS**
RDA093	-44.74	-4.43	-3.72	-3.85	-12.05	-0.73	-0.05	3.87	3.54	-2.62	1.36	-3.66	-3.33	-4.54	-4.68	-5.48	-6.01	-6.54	-3.54	-2.24
RDA094	-31.59	-7.05	-6.19	-5.99	-14.31	-2.78	-2.12	2.28	2.24	-3.23	-0.60	-6.60	-6.49	-7.06	-7.13	-8.01	-8.38	-8.83	-5.92	-5.77
RDA095	-35.82	-7.05	-6.19	-5.99	-14.31	-2.78	-2.12	2.28	2.24	-3.23	-0.60	-6.60	-6.49	-7.06	-7.13	-8.01	-8.38	-8.83	-5.92	-5.77
RDA096	-42.62	-4.43	-3.72	-3.85	-12.05	-0.73	-0.05	3.87	3.54	-2.62	1.36	-3.66	-3.33	-4.54	-4.68	-5.48	-6.01	-6.54	-3.54	-2.24
RDA097	-87.64	-31.31	-43.31	-43.24	-41.25	-31.18	-14.30	-5.41	5.16	5.40	-4.81	-18.67	-22.73	-33.10	-35.50	-36.37	-39.78	-42.60	-26.33	-28.66
RDA098	-34.41	-36.93	-50.03	-50.13	-41.73	-23.64	-11.62	-1.82	16.43	12.55	-4.76	-22.67	-31.39	-36.65	-38.97	-42.04	-44.70	-47.30	-38.07	-33.01
RDA099	-56.98	-6.94	-4.06	-5.62	-14.11	-2.44	-2.17	2.31	1.80	-2.33	-0.50	-7.05	-8.97	-7.03	-7.02	-7.94	-7.68	-7.93	-3.95	-4.84
RDA100	-54.82	-6.94	-4.06	-5.62	-14.11	-2.44	-2.17	2.31	1.80	-2.33	-0.50	-7.05	-8.97	-7.03	-7.02	-7.94	-7.68	-7.93	-3.95	-4.84
RDA101	-48.54	-3.46	-2.03	-2.13	-11.17	-0.04	-0.17	3.97	3.15	-2.15	1.27	-3.70	-4.35	-3.84	-3.86	-4.63	-4.70	-5.11	-2.24	-1.88
RDA102	-81.74	-9.74	-15.85	-20.69	-28.29	-14.73	-2.03	0.77	7.57	5.06	5.79	-2.80	-5.03	-13.87	-15.72	-15.92	-18.64	-21.61	-13.58	-12.99
RDA103	-44.02	-9.74	-15.85	-20.69	-28.29	-14.73	-2.03	0.77	7.57	5.06	5.79	-2.80	-5.03	-13.87	-15.72	-15.92	-18.64	-21.61	-13.58	-12.99
RDA104	-79.94	-10.82	-16.72	-21.71	-27.79	-14.35	-1.39	1.23	8.19	5.09	6.14	-1.73	-4.79	-13.99	-16.03	-16.37	-19.26	-22.07	-12.86	-14.25
RDA105	-74.07	-10.82	-16.72	-21.71	-27.79	-14.35	-1.39	1.23	8.19	5.09	6.14	-1.73	-4.79	-13.99	-16.03	-16.37	-19.26	-22.07	-12.86	-14.25
RDA106	-94.94	-13.79	-21.25	-26.10	-34.18	-21.43	-9.45	-5.51	0.56	0.03	-0.16	-9.53	-11.60	-19.70	-21.39	-21.39	-23.83	-27.18	-18.75	-17.58
RDA107	-57.80	-13.79	-21.25	-26.10	-34.18	-21.43	-9.45	-5.51	0.56	0.03	-0.16	-9.53	-11.60	-19.70	-21.39	-21.39	-23.83	-27.18	-18.75	-17.58
RDA108	-75.67	-1.61	-3.82	-8.12	-11.81	-19.31	-14.89	-8.35	-10.76	1.44	-2.69	-11.28	-6.01	-8.85	-8.45	-6.28	-6.34	-7.92	-14.65	-9.79
RDA109	-44.71	-1.61	-3.82	-8.12	-11.81	-19.31	-14.89	-8.35	-10.76	1.44	-2.69	-11.28	-6.01	-8.85	-8.45	-6.28	-6.34	-7.92	-14.65	-9.79
RDA110	-74.41	-9.79	-16.40	-21.30	-29.29	-15.52	-3.26	-0.02	5.91	4.59	5.10	-3.71	-6.12	-14.59	-16.40	-16.58	-19.20	-22.33	-14.09	-12.71
RDA111	-80.04	-8.76	-14.83	-19.20	-31.22	-15.34	-2.95	0.01	6.92	4.82	5.09	-4.48	-4.15	-14.00	-15.58	-15.63	-18.50	-21.75	-15.01	-13.49
RDA112	-52.42	-15.67	-22.60	-23.23	-26.39	-12.92	-1.17	1.88	8.38	7.88	3.55	-5.76	-12.07	-16.95	-18.82	-19.99	-21.97	-24.08	-9.43	-13.96
RDA113	-56.43	-15.49	-22.50	-23.23	-26.18	-12.59	-0.84	2.07	8.62	8.26	3.59	-5.85	-12.23	-16.87	-18.70	-19.93	-21.85	-23.97	-9.35	-13.90
RDA114	-62.84	-38.63	-51.77	-51.65	-43.22	-25.79	-13.26	-3.26	14.22	10.63	-6.12	-24.03	-32.05	-38.16	-40.52	-43.46	-46.32	-48.88	-39.76	-33.77
RDA115	-65.65	-35.82	-48.29	-48.79	-40.45	-22.92	-10.40	-1.98	13.64	11.48	-4.61	-22.06	-30.31	-35.52	-37.76	-40.73	-43.34	-45.84	-37.85	-30.91
RDA116	-72.93	-38.14	-52.18	-51.35	-44.02	-25.41	-14.34	-2.35	18.42	12.77	-5.70	-23.88	-33.60	-38.37	-40.78	-43.85	-46.42	-49.18	-37.83	-35.68
RDA117	-80.64	-39.85	-60.95	-51.30	-51.00	-37.95	-23.01	-11.46	-23.75	-0.28	-15.03	-24.84	-21.87	-41.11	-43.82	-44.99	-50.78	-54.42	-39.08	-36.01
RDA118	-107.33	-39.85	-60.95	-51.30	-51.00	-37.95	-23.01	-11.46	-23.75	-0.28	-15.03	-24.84	-21.87	-41.11	-43.82	-44.99	-50.78	-54.42	-39.08	-36.01
RDA119	-73.00	-27.92	-36.06	-41.53	-38.58	-39.86	-25.71	-21.01	-14.82	-9.18	-16.64	-22.02	-29.91	-33.70	-35.64	-34.80	-36.02	-38.72	-27.32	-26.41
RDA120	-78.77	-29.34	-37.79	-42.12	-39.25	-41.42	-26.10	-21.19	-15.49	-8.98	-17.73	-22.05	-31.01	-34.71	-36.82	-35.90	-37.13	-39.72	-27.89	-27.03
RDA121	-64.30	-18.00	-26.00	-32.00	-39.00	-47.00	-39.00	-34.00	-27.00	-22.00	-23.00	-24.00	-27.00	-30.43	-31.50	-28.40	-28.75	-32.33	-29.83	-27.00
RDA122	-61.43	-28.23	-36.77	-38.68	-37.40	-41.72	-24.03	-20.09	-13.71	-7.05	-16.91	-18.84	-29.36	-33.00	-35.36	-34.09	-35.27	-37.62	-25.41	-25.40
RDA124	-69.27	-27.68	-36.20	-38.23	-36.90	-41.14	-23.53	-19.68	-13.35	-6.65	-16.39	-18.32	-28.82	-32.47	-34.83	-33.57	-34.75	-37.11	-25.00	-24.99

		δ^2H_p (‰)																		
**ID**	**δ^2H_f (‰)**	**Jan**	**Feb**	**Mar**	**Apr**	**May**	**Jun**	**Jul**	**Aug**	**Sep**	**Oct**	**Nov**	**Dec**	**Nov-May**	**Dec-May**	**Dec-April**	**Jan-April**	**Feb-April**	**Annual**	**GS**
RDA125	-79.60	-49.85	-29.85	-52.37	-41.31	-24.60	-18.83	-15.64	-9.71	-5.17	-15.33	-35.71	-46.95	-40.09	-40.82	-44.07	-43.35	-41.18	-39.05	-37.75
RDA126	-62.69	-36.59	-46.10	-47.05	-45.88	-48.26	-31.22	-24.36	-17.44	-11.83	-22.79	-28.52	-37.81	-41.46	-43.61	-42.69	-43.90	-46.34	-33.29	-32.19
RDA127	-97.39	-24.02	-36.63	-53.25	-41.36	-33.42	-27.92	-18.68	-11.56	-9.71	-14.06	-21.75	-33.13	-34.80	-36.97	-37.68	-38.82	-43.75	-33.55	-28.98
RDA128	-49.26	-35.74	-28.59	-35.40	-27.76	-20.75	-10.14	-13.21	-6.82	-3.67	-10.24	-21.32	-26.76	-28.05	-29.17	-30.85	-31.87	-30.58	-22.50	-21.89
RDA129	-60.92	-29.00	-16.58	-35.00	-26.00	-16.00	-16.00	-20.00	-16.00	-8.00	-10.00	-19.00	-22.00	-23.37	-24.10	-25.72	-26.65	-25.86	-19.47	-24.00
RDA130	-57.51	-29.00	-16.58	-35.00	-26.00	-16.00	-16.00	-20.00	-16.00	-8.00	-10.00	-19.00	-22.00	-23.37	-24.10	-25.72	-26.65	-25.86	-19.47	-24.00
RDA131	-79.01	-38.34	-43.71	-51.46	-54.27	-41.88	-21.26	-12.43	-5.78	-1.03	-10.58	-30.01	-40.66	-42.90	-45.05	-45.69	-46.95	-49.81	-33.92	-33.82
RDA132	-88.72	-38.34	-43.71	-51.46	-54.27	-41.88	-21.26	-12.43	-5.78	-1.03	-10.58	-30.01	-40.66	-42.90	-45.05	-45.69	-46.95	-49.81	-33.92	-33.82
RDA133	-81.33	-35.37	-37.77	-46.73	-50.28	-35.82	-15.89	-7.41	0.83	4.64	-6.10	-25.39	-35.69	-38.15	-40.27	-41.17	-42.54	-44.93	-30.43	-30.79
RDA134	-98.84	-35.37	-37.77	-46.73	-50.28	-35.82	-15.89	-7.41	0.83	4.64	-6.10	-25.39	-35.69	-38.15	-40.27	-41.17	-42.54	-44.93	-30.43	-30.79
RDA135	-59.16	-35.37	-37.77	-46.73	-50.28	-35.82	-15.89	-7.41	0.83	4.64	-6.10	-25.39	-35.69	-38.15	-40.27	-41.17	-42.54	-44.93	-30.43	-30.79
RDA136	-25.06	-35.37	-37.77	-46.73	-50.28	-35.82	-15.89	-7.41	0.83	4.64	-6.10	-25.39	-35.69	-38.15	-40.27	-41.17	-42.54	-44.93	-30.43	-30.79
RDA137	-51.96	-47.88	-23.03	-51.92	-34.23	-16.48	-12.06	-9.91	-8.23	-1.58	-8.96	-30.70	-42.23	-35.21	-35.96	-39.86	-39.26	-36.39	-39.94	-33.74
RDA138	-53.35	-47.88	-23.03	-51.92	-34.23	-16.48	-12.06	-9.91	-8.23	-1.58	-8.96	-30.70	-42.23	-35.21	-35.96	-39.86	-39.26	-36.39	-39.94	-33.74
RDA139	-58.97	-47.88	-23.03	-51.92	-34.23	-16.48	-12.06	-9.91	-8.23	-1.58	-8.96	-30.70	-42.23	-35.21	-35.96	-39.86	-39.26	-36.39	-39.94	-33.74
RDA140	-66.50	-47.88	-23.03	-51.92	-34.23	-16.48	-12.06	-9.91	-8.23	-1.58	-8.96	-30.70	-42.23	-35.21	-35.96	-39.86	-39.26	-36.39	-39.94	-33.74
RDA141	-52.09	-24.42	-31.57	-33.85	-34.95	-17.58	-6.35	-4.46	-0.80	5.55	-9.81	-25.10	-36.32	-29.11	-29.78	-32.22	-31.20	-33.46	-28.02	-26.58
RDA142	-93.14	-24.42	-31.57	-33.85	-34.95	-17.58	-6.35	-4.46	-0.80	5.55	-9.81	-25.10	-36.32	-29.11	-29.78	-32.22	-31.20	-33.46	-28.02	-26.58
RDA143	-38.92	-24.42	-31.57	-33.85	-34.95	-17.58	-6.35	-4.46	-0.80	5.55	-9.81	-25.10	-36.32	-29.11	-29.78	-32.22	-31.20	-33.46	-28.02	-26.58
RDA144	-54.40	-24.42	-31.57	-33.85	-34.95	-17.58	-6.35	-4.46	-0.80	5.55	-9.81	-25.10	-36.32	-29.11	-29.78	-32.22	-31.20	-33.46	-28.02	-26.58
RDA145	-100.65	-33.48	-47.18	-46.71	-43.27	-24.74	-14.17	-1.34	17.33	12.88	-4.22	-21.34	-33.34	-35.72	-38.12	-40.80	-42.66	-45.72	-32.46	-30.58
RDA146	-42.83	-33.48	-47.18	-46.71	-43.27	-24.74	-14.17	-1.34	17.33	12.88	-4.22	-21.34	-33.34	-35.72	-38.12	-40.80	-42.66	-45.72	-32.46	-30.58
RDA147	-83.68	-33.48	-47.18	-46.71	-43.27	-24.74	-14.17	-1.34	17.33	12.88	-4.22	-21.34	-33.34	-35.72	-38.12	-40.80	-42.66	-45.72	-32.46	-30.58
RDA148	-71.79	-33.48	-47.18	-46.71	-43.27	-24.74	-14.17	-1.34	17.33	12.88	-4.22	-21.34	-33.34	-35.72	-38.12	-40.80	-42.66	-45.72	-32.46	-30.58
RDA149	-87.31	-33.48	-47.18	-46.71	-43.27	-24.74	-14.17	-1.34	17.33	12.88	-4.22	-21.34	-33.34	-35.72	-38.12	-40.80	-42.66	-45.72	-32.46	-30.58
RDA150	-78.29	-30.45	-38.12	-38.68	-37.40	-21.26	-10.19	-10.10	-3.74	-0.28	-15.19	-31.88	-42.99	-34.40	-34.82	-37.53	-36.16	-38.07	-33.21	-32.47
RDA151	-54.31	-30.45	-38.12	-38.68	-37.40	-21.26	-10.19	-10.10	-3.74	-0.28	-15.19	-31.88	-42.99	-34.40	-34.82	-37.53	-36.16	-38.07	-33.21	-32.47
RDA152	-63.67	-31.44	-39.59	-39.66	-39.25	-22.64	-11.51	-10.89	-6.06	-0.76	-17.43	-33.13	-44.20	-35.70	-36.13	-38.83	-37.49	-39.50	-33.74	-33.45
RDA153	-67.09	-31.44	-39.59	-39.66	-39.25	-22.64	-11.51	-10.89	-6.06	-0.76	-17.43	-33.13	-44.20	-35.70	-36.13	-38.83	-37.49	-39.50	-33.74	-33.45
RDA154	-48.90	-31.44	-39.59	-39.66	-39.25	-22.64	-11.51	-10.89	-6.06	-0.76	-17.43	-33.13	-44.20	-35.70	-36.13	-38.83	-37.49	-39.50	-33.74	-33.45
RDA155	-61.39	-31.44	-39.59	-39.66	-39.25	-22.64	-11.51	-10.89	-6.06	-0.76	-17.43	-33.13	-44.20	-35.70	-36.13	-38.83	-37.49	-39.50	-33.74	-33.45

		δ^2H_p (‰)																		
**ID**	**δ^2H_f (‰)**	**Jan**	**Feb**	**Mar**	**Apr**	**May**	**Jun**	**Jul**	**Aug**	**Sep**	**Oct**	**Nov**	**Dec**	**Nov-May**	**Dec-May**	**Dec-April**	**Jan-April**	**Feb-April**	**Annual**	**GS**
RDA156	-56.16	-43.99	-57.73	-55.41	-50.94	-32.78	-21.89	-8.64	12.57	5.88	-11.90	-30.93	-42.18	-44.85	-47.17	-50.05	-52.02	-54.69	-41.50	-39.30
RDA157	-60.76	-41.49	-55.48	-53.78	-48.96	-30.57	-19.70	-6.98	13.82	7.55	-9.67	-28.59	-40.07	-42.71	-45.06	-47.96	-49.93	-52.74	-38.84	-37.02
RDA158	-81.03	-41.49	-55.48	-53.78	-48.96	-30.57	-19.70	-6.98	13.82	7.55	-9.67	-28.59	-40.07	-42.71	-45.06	-47.96	-49.93	-52.74	-38.84	-37.02
RDA159	-60.89	-43.39	-57.19	-55.04	-50.44	-32.21	-21.28	-8.24	12.86	6.29	-11.32	-30.33	-41.65	-44.32	-46.65	-49.54	-51.51	-54.22	-40.79	-38.71
RDA160	-68.66	-29.59	-37.81	-38.82	-37.61	-19.46	-8.63	-6.84	-2.27	2.99	-14.09	-29.95	-40.71	-33.42	-34.00	-36.91	-35.96	-38.08	-35.21	-31.61
RDA161	-82.32	-29.59	-37.81	-38.82	-37.61	-19.46	-8.63	-6.84	-2.27	2.99	-14.09	-29.95	-40.71	-33.42	-34.00	-36.91	-35.96	-38.08	-35.21	-31.61
RDA162	-63.65	-29.35	-37.55	-38.62	-37.44	-19.32	-8.49	-6.73	-2.25	3.08	-13.93	-29.73	-40.55	-33.22	-33.80	-36.70	-35.74	-37.87	-34.95	-31.38
RDA163	-55.13	-29.35	-37.55	-38.62	-37.44	-19.32	-8.49	-6.73	-2.25	3.08	-13.93	-29.73	-40.55	-33.22	-33.80	-36.70	-35.74	-37.87	-34.95	-31.38
RDA164	-66.52	-16.50	-21.70	-22.21	-25.70	-14.43	-4.05	-0.28	5.69	4.05	1.96	-5.81	-10.46	-16.69	-18.50	-19.32	-21.53	-23.21	-11.46	-14.75
RDA165	-96.85	-16.50	-21.70	-22.21	-25.70	-14.43	-4.05	-0.28	5.69	4.05	1.96	-5.81	-10.46	-16.69	-18.50	-19.32	-21.53	-23.21	-11.46	-14.75
RDA166	-48.90	-15.71	-21.07	-21.68	-25.22	-13.74	-2.93	0.62	6.61	4.84	3.40	-4.12	-9.15	-15.81	-17.76	-18.56	-20.92	-22.65	-9.89	-13.90
RDA167	-42.82	-15.71	-21.07	-21.68	-25.22	-13.74	-2.93	0.62	6.61	4.84	3.40	-4.12	-9.15	-15.81	-17.76	-18.56	-20.92	-22.65	-9.89	-13.90
RDA168	-75.70	-10.00	-15.00	-20.00	-30.00	-17.00	-7.00	-5.00	0.00	2.00	1.00	-9.00	-6.00	-15.29	-16.33	-16.20	-18.75	-21.67	-9.67	-18.00
RDA169	-80.38	-10.00	-15.00	-20.00	-30.00	-17.00	-7.00	-5.00	0.00	2.00	1.00	-9.00	-6.00	-15.29	-16.33	-16.20	-18.75	-21.67	-9.67	-18.00
RDA170	-60.50	-20.08	-27.14	-36.51	-46.71	-47.06	-20.29	-11.13	-1.78	-2.68	-5.37	-19.73	-15.42	-30.38	-32.15	-29.17	-32.61	-36.79	-31.10	-27.47
RDA171	-85.20	-20.00	-27.24	-36.67	-46.22	-46.43	-19.44	-10.61	-1.41	-2.27	-4.81	-19.04	-15.42	-30.15	-32.00	-29.11	-32.53	-36.71	-30.27	-27.06
RDA172	-55.72	-2.95	-2.18	-2.59	-10.89	0.09	0.78	4.55	4.17	-2.61	1.79	-2.44	-2.72	-3.38	-3.54	-4.27	-4.65	-5.22	-1.91	-1.32
RDA173	-45.69	-2.95	-2.18	-2.59	-10.89	0.09	0.78	4.55	4.17	-2.61	1.79	-2.44	-2.72	-3.38	-3.54	-4.27	-4.65	-5.22	-1.91	-1.32
RDA174	-40.27	-7.43	-7.76	-8.62	-16.69	-8.33	-4.49	-0.78	-0.60	-1.39	0.92	-3.88	-3.12	-7.98	-8.66	-8.73	-10.13	-11.03	-8.25	-6.03
RDA175	-26.70	-7.38	-7.58	-8.38	-16.22	-7.81	-4.05	-0.36	0.02	-1.20	1.11	-3.72	-2.88	-7.71	-8.38	-8.49	-9.89	-10.73	-7.92	-5.99
RDA176	-61.08	-24.54	-31.84	-40.57	-44.67	-42.70	-20.32	-14.74	-8.00	-7.03	-8.08	-21.21	-21.22	-32.39	-34.26	-32.57	-35.40	-39.03	-31.87	-31.39
RDA177	-59.74	-24.54	-31.84	-40.57	-44.67	-42.70	-20.32	-14.74	-8.00	-7.03	-8.08	-21.21	-21.22	-32.39	-34.26	-32.57	-35.40	-39.03	-31.87	-31.39
RDA178	-60.04	-24.54	-31.84	-40.57	-44.67	-42.70	-20.32	-14.74	-8.00	-7.03	-8.08	-21.21	-21.22	-32.39	-34.26	-32.57	-35.40	-39.03	-31.87	-31.39
RDA179	-60.56	-20.27	-28.78	-38.88	-45.22	-45.58	-19.95	-10.02	-1.67	-1.52	-4.19	-17.05	-17.46	-30.46	-32.70	-30.12	-33.29	-37.63	-27.91	-26.17
RDA180	-47.36	-20.35	-29.09	-39.11	-45.15	-45.48	-19.69	-9.93	-1.60	-1.45	-4.15	-16.94	-17.31	-30.49	-32.75	-30.20	-33.43	-37.79	-27.74	-26.25
RDA181	-57.89	-20.27	-28.78	-38.88	-45.22	-45.58	-19.95	-10.02	-1.67	-1.52	-4.19	-17.05	-17.46	-30.46	-32.70	-30.12	-33.29	-37.63	-27.91	-26.17
RDA182	-48.66	-26.15	-29.15	-42.02	-51.01	-66.81	-44.64	-27.48	-15.82	-16.60	-21.55	-33.88	-26.09	-39.30	-40.21	-34.89	-37.08	-40.73	-38.64	-37.71
RDA183	-45.18	-26.15	-29.15	-42.02	-51.01	-66.81	-44.64	-27.48	-15.82	-16.60	-21.55	-33.88	-26.09	-39.30	-40.21	-34.89	-37.08	-40.73	-38.64	-37.71
RDA184	-61.65	-21.96	-30.96	-40.70	-45.87	-46.78	-19.43	-10.78	-2.24	-1.83	-5.21	-17.75	-17.76	-31.68	-34.01	-31.45	-34.87	-39.18	-27.76	-27.34
RDA185	-72.69	-22.42	-30.50	-40.31	-45.20	-46.31	-18.77	-10.63	-1.84	-2.47	-4.77	-17.68	-17.11	-31.36	-33.64	-31.10	-34.60	-38.67	-27.37	-27.28
RDA186	-93.53	-22.42	-30.50	-40.31	-45.20	-46.31	-18.77	-10.63	-1.84	-2.47	-4.77	-17.68	-17.11	-31.36	-33.64	-31.10	-34.60	-38.67	-27.37	-27.28

		δ^2H_p (‰)																		
**ID**	**δ^2H_f (‰)**	**Jan**	**Feb**	**Mar**	**Apr**	**May**	**Jun**	**Jul**	**Aug**	**Sep**	**Oct**	**Nov**	**Dec**	**Nov-May**	**Dec-May**	**Dec-April**	**Jan-April**	**Feb-April**	**Annual**	**GS**
RDA187	-47.66	-22.42	-30.50	-40.31	-45.20	-46.31	-18.77	-10.63	-1.84	-2.47	-4.77	-17.68	-17.11	-31.36	-33.64	-31.10	-34.60	-38.67	-27.37	-27.28
RDA188	-60.37	-22.18	-31.15	-40.87	-46.05	-46.98	-19.58	-10.95	-2.38	-1.91	-5.39	-17.97	-17.94	-31.88	-34.20	-31.64	-35.06	-39.36	-27.89	-27.48
RDA189	-47.15	-19.41	-26.43	-37.29	-45.40	-47.81	-23.34	-11.65	-2.81	-2.52	-5.23	-17.78	-16.37	-30.07	-32.12	-28.98	-32.13	-36.37	-29.72	-26.00
RDA190	-51.42	-12.75	-15.53	-34.07	-43.50	-58.81	-37.74	-18.98	-9.52	-7.23	-9.45	-16.66	-15.33	-28.09	-30.00	-24.24	-26.46	-31.03	-27.64	-27.13
RDA191	-71.77	-28.46	-30.22	-37.12	-39.35	-23.16	-9.89	-5.62	1.27	4.71	-8.46	-25.28	-36.33	-31.42	-32.44	-34.30	-33.79	-35.56	-27.03	-27.61
RDA192	-55.11	-28.46	-30.22	-37.12	-39.35	-23.16	-9.89	-5.62	1.27	4.71	-8.46	-25.28	-36.33	-31.42	-32.44	-34.30	-33.79	-35.56	-27.03	-27.61
RDA193	-52.51	-29.71	-37.70	-37.92	-36.50	-20.48	-9.34	-10.01	-5.71	0.15	-17.38	-31.50	-42.72	-33.79	-34.17	-36.91	-35.46	-37.37	-32.27	-32.22
