# Supplementary material for: A feather hydrogen (δ2H) isoscape for Brazil
Source: PLoS One. 2022 Aug 3;17(8):e0271573. doi: 10.1371/journal.pone.0271573 (PMC9348672; doi:10.1371/journal.pone.0271573)

S5 File. Modeling the Brazilian Hydrogen Isoscape.
First step- Prepare data. R code to input raster and observations, and extract values. Including S4 Table with extracted values; Second step- Selection of variables using Recursive Feature Elimination – RFE. R code to run RFE and plot results; Third step- Prepare data and run Random Forest (RF). R code to run RF and explore results with partial plots; Fourth step- Model validation. R code to validate the model and plot results; Fifth step- Spatial prediction. R code to apply modeled values into a isoscape, calculate method uncertainty, mean, standard deviation and coefficient of variation.
This R script was adapted from the script provided by Bataille et al. 2018 and Sena-Souza et al. 2020

Bataille, C.P.; von Holstein, I.C.C.; Laffoon, J.E.; Willmes, M.; Liu, X.M.; Davies, G. R. 2018. A bioavailable strontium isoscape for Western Europe: A machine learning approach. PLoS ONE 13:1–27.

Sena-Souza, J.P.; Houlton, B. Z.; Martinelli, L. A.; Nardoto, G. B. 2020. Reconstructing continental-scale variation in soil δ N: a machine learning approach in South America. Ecosphere, 11: e03223.

First Step - Prepare data

1- Input Raster

Obs: All rasters had already been extracted for Brazil and organized in the same folder. Rasters were also resampled to have the same number of cells (resample() function in raster package), and cropped to have the same size (mask() and crop() functions in raster package)

Datasets:
WORLDCLIM (2007-2018) Raster source: Worldclim (https://www.worldclim.org/data/monthlywth.html) Maximum and Minimum Temperature, and Precipitation (See Suppl. Material B to see how these variables were calculated for the timeframe of interest)

```
Library(raster)
Bio1<-raster("Bio1_Annual_Mean_Temp_2007_2018.tif")
Bio2<-raster("Bio2_Mean_diurnal_range_Temp_2007_2018.tif")
Bio3<-raster("Bio3_Isotermality_2007_2018.tif")
Bio4<-raster("Bio4_Temp_seasonality_2007_2018.tif")
Bio5<-raster("Bio5_Max_temp_warmest_month_2007_2018.tif")
Bio6<-raster("Bio6_Min_temp_coldest_month_2007_2018.tif")
Bio7<-raster("Bio7_Temp_annual_range_2007_2018.tif")
Bio8<-raster("Bio8_Mean_temp_wettest_quarter_2007_2018.tif")
Bio9<-raster("Bio9_Mean_temp_driest_quarter_2007_2018.tif")
Bio10<-raster("Bio10_Mean_temp_warmest_quarter_2007_2018.tif")
Bio11<-raster("Bio11_Mean_temp_coldest_quarter_2007_2018.tif")
Bio12<-raster("Bio12_Annual_precipitation_2007_2018.tif")
Bio13<-raster("Bio13_Prec_wettest_month_2007_2018.tif")
Bio14<-raster("Bio14_Prec_driest_month_2007_2018.tif")
Bio15<-raster("Bio15_Prec_seasonality_2007_2018.tif")
Bio16<-raster("Bio16_Prec_wettest_quarter_2007_2018.tif")
Bio17<-raster("Bio17_Prec_driest_quarter_2007_2018.tif")
Bio18<-raster("Bio18_Prec_warmest_quarter_2007_2018.tif")
Bio19<-raster("Bio19_Prec_coldest_quarter_2007_2018.tif")

plot(Bio1) . . .
```

Bio1_Annual_Mean_Temp_2007_2018

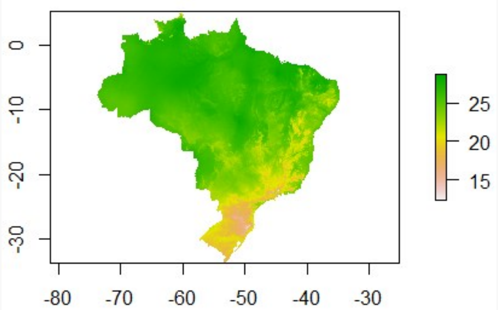

Bio2_Mean_diurnal_range_Temp_2007_2018

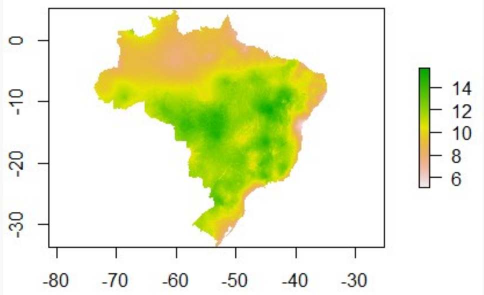

Bio3_Isotermality_2007_2018

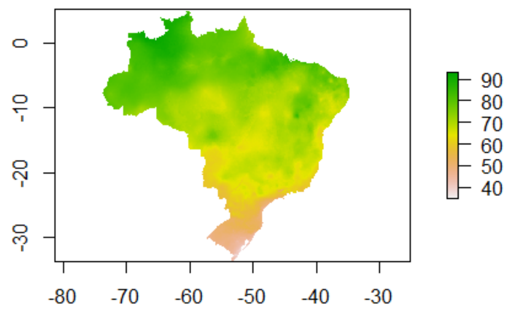

Bio4_Temp_seasonality_2007_2018

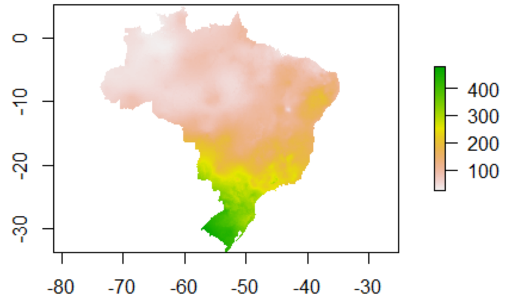

Bio5_Max_temp_warmest_month_2007_2018

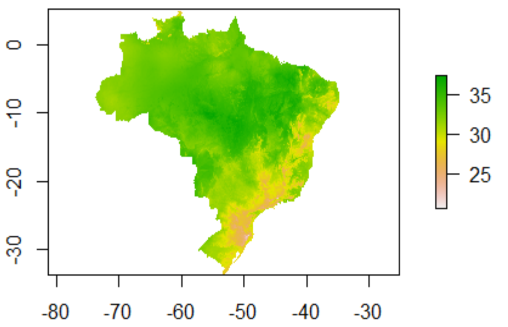

Bio6_Min_temp_coldest_month_2007_2018

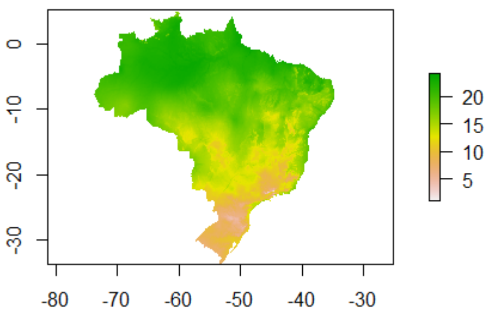

Bio7_Temp_annual_range_2007_2018

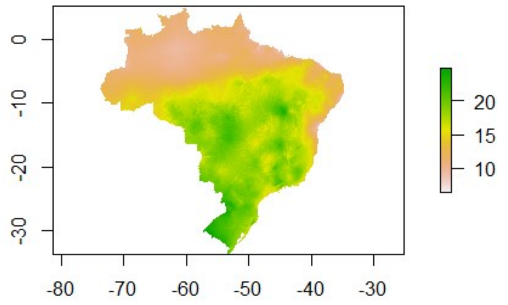

Bio8_Mean_temp_wettest_quarter_2007_2018

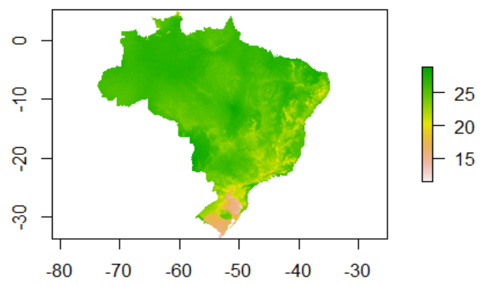

Bio9_Mean_temp_driest_quarter_2007_2018

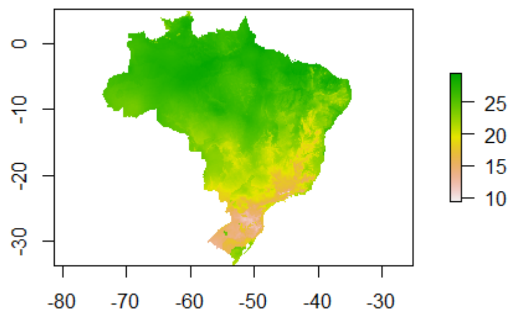

Bio10_Mean_temp_warmest_quarter_2007_2018

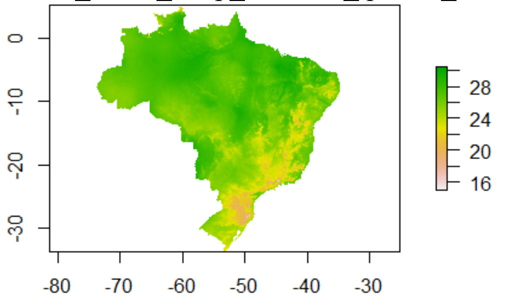

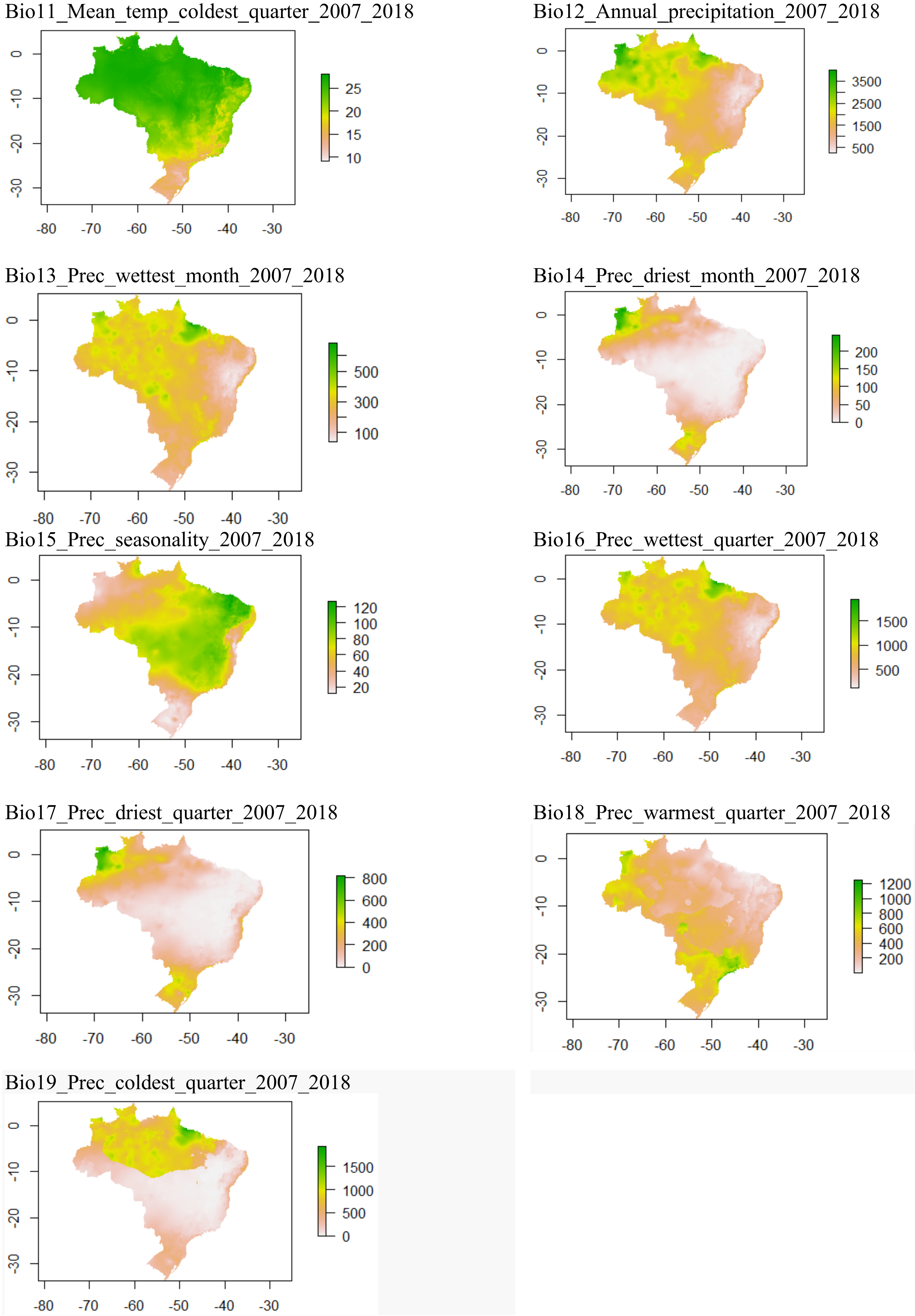
Bio11_Mean_temp_coldest_quarter_2007_2018
25
20
15
10
Bio12_Annual_precipitation_2007_2018
3500
2500
1500
500
Bio13_Prec_wettest_month_2007_2018
500
300
100
Bio14_Prec_driest_month_2007_2018
200
150
100
50
0
Bio15_Prec_seasonality_2007_2018
120
100
80
60
40
20
Bio16_Prec_wettest_quarter_2007_2018
1500
1000
500
Bio17_Prec_driest_quarter_2007_2018
800
600
400
200
0
Bio18_Prec_warmest_quarter_2007_2018
1200
1000
800
600
400
200
Bio19_Prec_coldest_quarter_2007_2018
1500
1000
500
0

WORLDCLIM (1970-2000) Raster source: Worldclim (https://www.worldclim.org/data/worldclim21.html)
Solar radiation, wind speed and water vapor pressure (1970-2000)

```
AM_windSpeed<-raster("R_Wind_resampled.grd")
AM_solarRad<-raster("R_Mean_Annual_srad_resampled.grd")
AM_vap<-raster("R_Mean_Annual_varp_resampled.grd")
Altitude<-raster("R_altitude_resampled.grd")
```

Mean_annual_Wind_speed_1970-2000

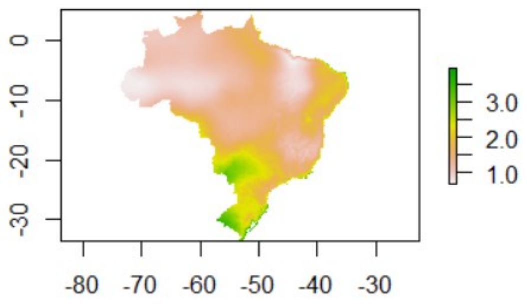

Mean_annual_Solar_Radiation_1970-2000

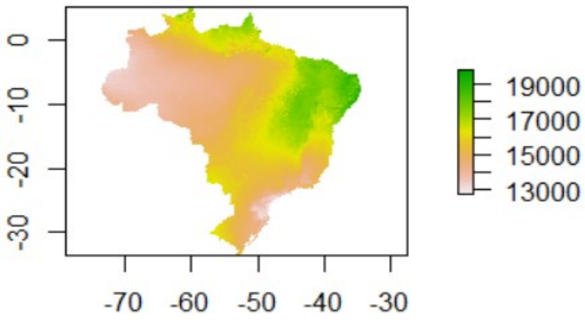

Annual_mean_Water_vapor_pressure_1970-2000

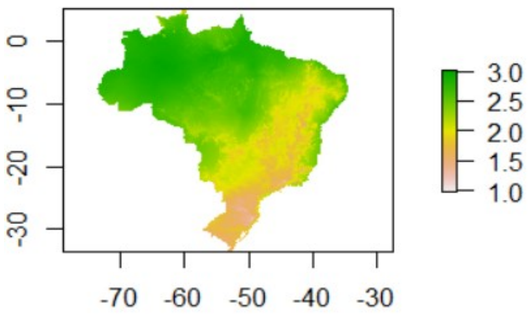

Altitude

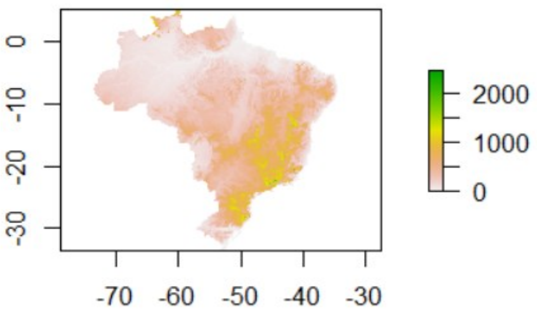

Climatic Research Unit (CRU) - (1901-2019) Raster source: CRU (http://www.cru.uea.ac.uk/data) Humidity, Potential evapotranpiration (1901-2019)

```
AM_humidity<-raster("R_humidity_resampled.grd")
AM_Evapo<-raster("R_Pot_evapotransp_resampled.grd")
```

Humidity_1901-2019

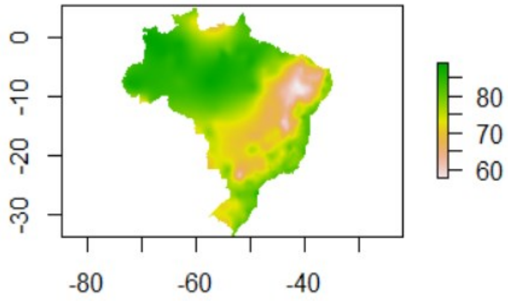

Potential_evapotranpiration_1901-2019

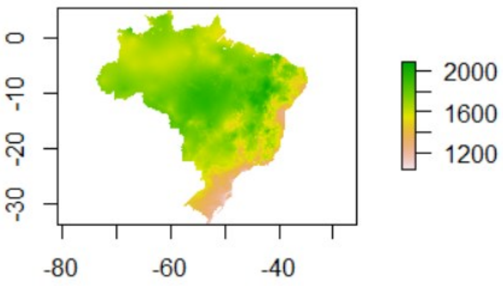

Waterisotope.org Raster source: https://wateriso.utah.edu/waterisotopes/index.html
Created raster with a timeframe of interest (See Suppl. Material C)

```
Prec_fev_abr_iso_Bowen<-raster("Prec_fev_abr_iso_Bowen-resampled.grd")
```

δ^2Hp February-April

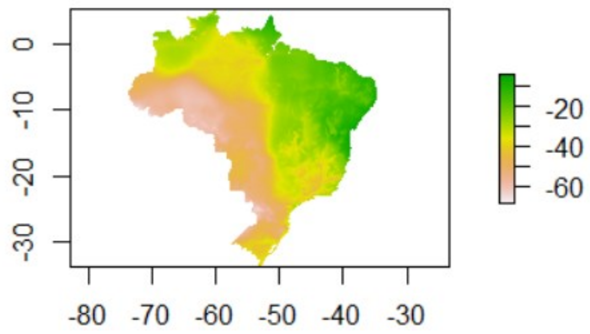

2- Input observations

- Open dataset and set coordinates (this table must contain latitude and longitude information)

```
d2H.orig <- read.table("Feather.txt", header = T)
y.red<-(d2H.orig$Lat_GD)
x.red<-(d2H.orig$Long_GD)
coord<-data.frame(x.red,y.red)
d2H.orig<-data.frame(d2H.orig,coord)
```

3- Extract values from raster

```
Bio1_xy<-extract(Bio1, d2H_spatial, method='bilinear', buffer=NULL,na.rm=TRUE)
Bio2_xy<-extract(Bio2, d2H_spatial, method='bilinear', buffer=NULL,na.rm=TRUE)
Bio3_xy<-extract(Bio3, d2H_spatial, method='bilinear', buffer=NULL,na.rm=TRUE)
Bio4_xy<-extract(Bio4, d2H_spatial, method='bilinear', buffer=NULL,na.rm=TRUE)
Bio5_xy<-extract(Bio5, d2H_spatial, method='bilinear', buffer=NULL,na.rm=TRUE)
Bio6_xy<-extract(Bio6, d2H_spatial, method='bilinear', buffer=NULL,na.rm=TRUE)
Bio7_xy<-extract(Bio7, d2H_spatial, method='bilinear', buffer=NULL,na.rm=TRUE)
Bio8_xy<-extract(Bio8, d2H_spatial, method='bilinear', buffer=NULL,na.rm=TRUE)
Bio9_xy<-extract(Bio9, d2H_spatial, method='bilinear', buffer=NULL,na.rm=TRUE)
Bio10_xy<-extract(Bio10, d2H_spatial, method='bilinear', buffer=NULL,na.rm=TRUE)
Bio11_xy<-extract(Bio11, d2H_spatial, method='bilinear', buffer=NULL,na.rm=TRUE)
Bio12_xy<-extract(Bio12, d2H_spatial, method='bilinear', buffer=NULL,na.rm=TRUE)
Bio13_xy<-extract(Bio13, d2H_spatial, method='bilinear', buffer=NULL,na.rm=TRUE)
Bio14_xy<-extract(Bio14, d2H_spatial, method='bilinear', buffer=NULL,na.rm=TRUE)
Bio15_xy<-extract(Bio15, d2H_spatial, method='bilinear', buffer=NULL,na.rm=TRUE)
Bio16_xy<-extract(Bio16, d2H_spatial, method='bilinear', buffer=NULL,na.rm=TRUE)
Bio17_xy<-extract(Bio17, d2H_spatial, method='bilinear', buffer=NULL,na.rm=TRUE)
Bio18_xy<-extract(Bio18, d2H_spatial, method='bilinear', buffer=NULL,na.rm=TRUE)
Bio19_xy<-extract(Bio19, d2H_spatial, method='bilinear', buffer=NULL,na.rm=TRUE)
AM_windSpeed_xy<-extract(AM_windSpeed, d2H_spatial, method='bilinear', buffer=NULL,na.rm=TRUE)
AM_solarRad_xy<-extract(AM_solarRad, d2H_spatial, method='bilinear', buffer=NULL,na.rm=TRUE)
AM_vap_xy<-extract(AM_vap, d2H_spatial, method='bilinear', buffer=NULL,na.rm=TRUE)
AM_humidity_xy<-extract(AM_humidity, d2H_spatial, method='bilinear', buffer=NULL,na.rm=TRUE)
AM_evapo_xy<-extract(AM_Evapo, d2H_spatial, method='bilinear', buffer=NULL,na.rm=TRUE)
Altitude_xy<-extract(Altitude, d2H_spatial, method='bilinear', buffer=NULL,na.rm=TRUE)
Bowen_Feb_April_xy<-extract(Prec_fev_abr_iso_Bowen, d2H_spatial, method='bilinear', buffer=NULL,na
.rm=TRUE)
```

- Combine dataset to create the regression matrix

```
d2H_proj_xy <- data.frame(d2H.orig$ID,d2H.orig$x.red,d2H.orig$y.red,d2H.orig$d2H.obs,Bio1_xy,Bio2_
xy,Bio3_xy,Bio4_xy,Bio5_xy,Bio6_xy,Bio7_xy,Bio8_xy,Bio9_xy,Bio10_xy,Bio11_xy,Bio12_xy,Bio13_xy,Bio
14_xy,Bio15_xy,Bio16_xy,Bio17_xy,Bio18_xy,Bio19_xy,AM_windSpeed_xy,AM_solarRad_xy,AM_vap_xy,AM_hum
idity_xy,AM_evapo_xy,Altitude_xy,Bowen_Feb_April_xy)
```

- Save the dataframe

```
write.table(d2H_proj_xy,file="d2H_proj_xy.txt”)
```

4- Explore and filter zero variance predictors (In this data we have no zero-variance data)

```
library(caret)

nzv <- nearZeroVar(d2H_proj_set[,-1:-3], saveMetrics= TRUE)
nzv

##                     freqRatio percentUnique zeroVar   nzv
## d2H.obs                     1      98.43750   FALSE FALSE
## Bio1_xy                     1      66.14583   FALSE FALSE
## Bio2_xy                     1      66.14583   FALSE FALSE
## Bio3_xy                     1      66.14583   FALSE FALSE
## Bio4_xy                     1      66.14583   FALSE FALSE
## Bio5_xy                     1      66.14583   FALSE FALSE
## Bio6_xy                     1      66.14583   FALSE FALSE
## Bio7_xy                     1      66.14583   FALSE FALSE
## Bio8_xy                     1      66.14583   FALSE FALSE
## Bio9_xy                     1      66.14583   FALSE FALSE
## Bio10_xy                    1      66.14583   FALSE FALSE
## Bio11_xy                    1      66.14583   FALSE FALSE
## Bio12_xy                    1      66.14583   FALSE FALSE
## Bio13_xy                    1      66.14583   FALSE FALSE
## Bio14_xy                    1      66.14583   FALSE FALSE
## Bio15_xy                    1      66.14583   FALSE FALSE
## Bio16_xy                    1      66.14583   FALSE FALSE
## Bio17_xy                    1      66.14583   FALSE FALSE
## Bio18_xy                    1      66.14583   FALSE FALSE
## Bio19_xy                    1      66.14583   FALSE FALSE
## AM_solarRad_xy              1      63.02083   FALSE FALSE
## AM_windSpeed_xy             1      66.14583   FALSE FALSE
## AM_vap_xy                   1      66.14583   FALSE FALSE
## Altitude_xy                 1      66.14583   FALSE FALSE
## AM_humidity_xy              1      66.14583   FALSE FALSE
## AM_evapo_xy                 1      66.14583   FALSE FALSE
## Bowen_Feb_April_xy          1      66.14583   FALSE FALSE
```

S5 Table. Bioclimatic variables values extracted from the same coordinates as our samples.

	WorldClim (2007-2018)																			WorldClim (1970-2000)					CRU (1901-2019)	
ID	Bio1	Bio2	Bio3	Bio4	Bio5	Bio6	Bio7	Bio8	Bio9	Bio10	Bio11	Bio12	Bio13	Bio14	Bio15	Bio16	Bio17	Bio18	Bio19	Altitude	SolarRad	Vapor	Wind	δ^2H_p	Hum	Evapo
RDA001	20.4	10.2	70.9	162.3	27.4	13.0	14.4	21.6	19.8	21.9	18.1	654.4	72.2	15.5	29.9	211.4	97.9	200.5	150.7	963.3	17002.8	1.8	2.0	-20.7	75.4	1456.8
RDA002	20.4	10.2	70.9	162.3	27.4	13.0	14.4	21.6	19.8	21.9	18.1	654.4	72.2	15.5	29.9	211.4	97.9	200.5	150.7	963.3	17002.8	1.8	2.0	-20.7	75.4	1456.8
RDA003	22.7	10.5	67.7	174.1	29.8	14.3	15.5	24.1	21.0	24.5	20.3	794.1	151.3	21.9	65.7	374.1	67.8	245.3	71.1	636.8	15819.5	2.0	1.5	-18.6	76.8	1571.0
RDA004	22.7	10.5	67.7	174.1	29.8	14.3	15.5	24.1	21.0	24.5	20.3	794.1	151.3	21.9	65.7	374.1	67.8	245.3	71.1	636.8	15819.5	2.0	1.5	-18.6	76.8	1571.0
RDA005	21.6	12.3	70.2	169.6	29.5	12.1	17.5	22.7	19.0	23.2	19.0	850.2	202.5	3.4	95.0	481.5	10.3	304.1	10.3	932.1	15616.5	1.8	1.3	-32.0	73.9	1620.0
RDA006	21.6	12.3	70.2	169.6	29.5	12.1	17.5	22.7	19.0	23.2	19.0	850.2	202.5	3.4	95.0	481.5	10.3	304.1	10.3	932.1	15616.5	1.8	1.3	-32.0	73.9	1620.0
RDA007	19.9	11.8	73.6	132.8	27.4	11.4	16.0	20.9	18.5	20.9	17.9	795.6	119.6	18.7	46.8	297.9	71.1	278.7	107.5	1164.5	16383.9	1.7	2.2	-22.6	74.0	1522.1
RDA008	19.9	11.8	73.6	132.8	27.4	11.4	16.0	20.9	18.5	20.9	17.9	795.6	119.6	18.7	46.8	297.9	71.1	278.7	107.5	1164.5	16383.9	1.7	2.2	-22.6	74.0	1522.1
RDA009	23.9	9.6	72.3	139.7	30.1	16.9	13.3	25.0	22.3	25.3	21.9	421.6	81.6	4.3	75.5	207.8	15.0	160.2	18.3	313.9	16461.3	2.2	1.7	-10.0	75.6	1537.7
RDA010	19.5	8.0	66.4	153.5	25.3	13.3	12.0	20.7	17.6	21.1	17.4	574.8	78.3	14.3	43.4	212.6	64.3	193.1	86.4	953.8	16708.4	1.8	2.0	-17.4	80.9	1234.0
RDA011	24.5	12.4	64.7	189.1	32.7	13.5	19.2	25.5	21.9	26.1	21.7	1531.4	297.3	20.0	72.7	752.7	72.0	366.2	102.3	360.5	15766.8	2.2	2.0	-47.7	71.3	1767.7
RDA012	25.9	12.2	66.3	153.4	34.1	15.7	18.4	26.8	23.7	27.1	23.6	1547.4	297.8	19.6	78.8	786.0	63.2	329.0	90.1	179.2	14621.4	2.3	1.4	-48.1	73.4	1763.9
RDA013	25.8	11.9	62.5	189.9	34.3	15.2	19.0	27.0	23.1	27.3	23.1	1399.5	281.4	20.8	78.7	719.3	66.6	460.2	92.6	127.8	15233.9	2.4	1.5	-45.6	74.5	1783.7
RDA014	26.7	9.7	78.2	79.7	33.6	21.2	12.4	25.8	27.7	27.8	25.8	2157.8	382.9	33.5	71.9	1026.9	117.5	146.9	947.7	137.3	14941.2	2.7	1.5	-28.1	85.2	1635.7
RDA015	26.7	9.7	78.2	79.7	33.6	21.2	12.4	25.8	27.7	27.8	25.8	2157.8	382.9	33.5	71.9	1026.9	117.5	146.9	947.7	137.3	14941.2	2.7	1.5	-28.1	85.2	1635.7
RDA016	23.6	13.2	69.1	111.7	32.3	13.2	19.1	23.9	22.4	24.8	22.0	1589.6	310.4	6.7	78.3	806.9	42.5	370.7	78.1	634.3	14593.7	2.3	2.1	-59.4	77.4	1725.3
RDA017	24.5	12.7	70.1	79.9	33.2	15.1	18.1	24.7	23.8	25.4	23.4	1519.7	297.3	4.1	83.7	829.9	34.2	371.5	64.9	339.6	14551.6	2.5	1.7	-59.3	80.3	1742.9
RDA018	26.4	9.6	78.4	75.1	33.2	21.0	12.2	25.6	27.4	27.5	25.6	2294.0	393.5	35.8	69.2	1059.5	130.4	153.3	977.5	90.1	14869.2	2.7	1.5	-29.4	85.4	1629.0
RDA019	23.6	13.0	69.0	115.1	32.0	13.2	18.8	23.9	22.2	24.8	21.8	1551.3	307.4	6.8	78.7	789.0	42.8	356.1	76.2	632.3	14589.5	2.3	2.1	-59.1	77.1	1736.3
RDA020	23.7	13.0	69.1	144.9	31.9	13.1	18.8	24.2	21.8	25.1	21.6	1525.3	307.0	13.0	77.1	761.5	59.1	322.9	86.9	616.2	14679.9	2.2	2.0	-57.0	75.6	1783.0
RDA021	24.6	12.6	70.2	79.9	33.2	15.2	18.0	24.7	23.9	25.4	23.4	1505.9	295.2	4.1	83.8	824.4	34.2	367.4	64.5	330.4	14578.8	2.5	1.7	-59.2	80.3	1744.7
RDA022	25.9	12.2	66.3	152.8	34.1	15.8	18.3	26.8	23.8	27.1	23.7	1531.3	293.4	19.4	78.4	776.2	63.6	325.3	91.1	196.4	14976.4	2.3	1.4	-48.3	73.4	1765.4
RDA023	24.6	14.3	71.4	92.6	33.8	13.8	20.0	24.8	23.4	25.4	23.4	1926.7	363.0	2.1	88.9	1039.8	25.4	423.8	38.7	421.8	15054.7	2.3	1.3	-50.1	76.5	1882.5
RDA024	23.7	13.0	69.1	144.9	31.9	13.1	18.8	24.2	21.8	25.1	21.6	1525.3	307.0	13.0	77.1	761.5	59.1	322.9	86.9	616.2	14679.9	2.2	2.0	-57.0	75.6	1783.0
RDA025	27.1	9.6	78.6	81.4	34.0	21.8	12.2	26.2	28.1	28.2	26.2	2146.3	377.4	33.6	71.1	1014.4	115.5	143.2	979.5	50.1	15037.2	2.8	1.5	-27.5	85.3	1659.5
RDA026	26.1	11.7	68.3	56.8	34.8	17.7	17.1	25.6	26.0	26.9	25.6	1822.3	304.4	2.8	75.4	873.5	43.3	230.5	822.3	229.5	14305.6	2.6	1.1	-52.5	85.1	1850.3
RDA027	26.1	11.7	68.3	56.8	34.8	17.7	17.1	25.6	26.0	26.9	25.6	1822.3	304.4	2.8	75.4	873.5	43.3	230.5	822.3	229.5	14305.6	2.6	1.1	-52.5	85.1	1850.3
RDA028	25.9	11.9	62.6	189.6	34.3	15.3	19.1	27.1	23.2	27.4	23.1	1402.9	281.4	21.3	78.4	719.2	67.0	462.3	92.5	129.0	15268.7	2.4	1.5	-45.6	74.6	1783.5
RDA029	25.2	7.2	67.7	129.3	30.6	20.0	10.6	24.0	25.8	26.5	23.4	1464.4	277.7	27.8	69.9	719.4	104.9	239.9	551.4	6.0	18831.8	2.7	2.6	-6.4	78.0	1379.1
RDA030	23.0	8.2	49.5	298.1	30.7	14.3	16.5	26.4	20.2	26.5	19.2	1960.3	295.6	66.9	47.8	824.6	246.8	762.2	280.5	58.0	13034.8	2.1	1.8	-30.7	82.3	1373.9
RDA031	24.6	8.1	68.1	147.4	30.8	18.9	11.9	23.2	25.4	26.1	22.6	1289.6	231.2	24.6	67.8	643.4	102.9	210.2	519.0	109.1	19043.6	2.5	1.7	-9.4	78.0	1455.3
RDA032	23.5	8.2	67.5	152.9	29.8	17.7	12.1	22.1	24.3	25.0	21.4	1224.3	229.6	20.5	70.2	623.5	90.8	186.2	497.7	346.7	18330.3	2.4	1.8	-11.2	77.9	1425.9
RDA033	24.3	8.3	67.1	158.5	30.7	18.3	12.4	22.9	25.2	25.9	22.2	979.8	193.2	17.3	71.6	496.8	69.9	152.5	379.9	146.4	19078.1	2.5	1.8	-8.9	77.6	1468.9

	WorldClim (2007-2018)																			WorldClim (1970-2000)					CRU (1901-2019)	
ID	Bio1	Bio2	Bio3	Bio4	Bio5	Bio6	Bio7	Bio8	Bio9	Bio10	Bio11	Bio12	Bio13	Bio14	Bio15	Bio16	Bio17	Bio18	Bio19	Altitude	SolarRad	Vapor	Wind	δ^2H_p	Hum	Evapo
RDA034	23.5	8.2	67.5	152.9	29.8	17.7	12.1	22.1	24.3	25.0	21.4	1224.3	229.6	20.5	70.2	623.5	90.8	186.2	497.7	346.7	18330.3	2.4	1.8	-11.2	77.9	1425.9
RDA035	24.6	8.1	68.1	147.4	30.8	18.9	11.9	23.2	25.4	26.1	22.6	1289.6	231.2	24.6	67.8	643.4	102.9	210.2	519.0	109.1	19043.6	2.5	1.7	-9.4	78.0	1455.3
RDA036	23.1	9.5	66.3	200.7	30.5	16.1	14.4	20.9	23.4	24.9	20.3	773.1	143.5	15.3	60.8	360.0	61.3	166.1	270.6	610.3	17628.2	2.1	2.1	-14.4	68.7	1563.4
RDA037	24.8	8.9	64.9	199.3	31.8	18.0	13.8	22.7	25.1	26.7	22.0	501.9	95.0	9.8	65.7	247.6	40.5	97.7	186.0	323.6	17842.5	2.3	1.9	-10.9	71.9	1529.0
RDA038	24.8	8.9	64.9	199.3	31.8	18.0	13.8	22.7	25.1	26.7	22.0	501.9	95.0	9.8	65.7	247.6	40.5	97.7	186.0	323.6	17842.5	2.3	1.9	-10.9	71.9	1529.0
RDA039	24.8	8.9	64.9	199.3	31.8	18.0	13.8	22.7	25.1	26.7	22.0	501.9	95.0	9.8	65.7	247.6	40.5	97.7	186.0	323.6	17842.5	2.3	1.9	-10.9	71.9	1529.0
RDA040	23.1	9.5	66.3	200.7	30.5	16.1	14.4	20.9	23.4	24.9	20.3	773.1	143.5	15.3	60.8	360.0	61.3	166.1	270.6	610.3	17628.2	2.1	2.1	-14.4	68.7	1563.4
RDA041	24.8	6.8	69.2	109.2	29.6	19.8	9.8	24.0	25.3	26.0	23.4	1634.2	259.6	34.9	61.1	750.7	126.5	358.4	432.1	45.1	18817.7	2.6	2.5	-6.9	79.0	1283.2
RDA042	25.1	7.1	70.7	107.7	30.0	20.0	10.0	24.2	25.6	26.3	23.7	1316.3	203.3	25.9	62.1	601.8	96.7	298.4	488.6	19.3	19196.3	2.6	2.5	-6.9	79.2	1300.3
RDA043	25.3	10.3	72.0	121.9	32.7	18.3	14.4	25.5	25.3	26.7	23.7	839.2	222.0	2.2	108.1	555.6	17.1	166.0	38.9	359.4	18105.0	2.1	1.8	-18.2	64.3	1717.7
RDA044	25.3	10.5	70.8	130.0	32.9	18.0	14.9	25.5	24.3	26.7	23.6	789.3	180.1	2.7	99.5	485.2	21.7	208.9	35.1	386.9	17705.7	2.1	1.8	-18.0	63.7	1708.1
RDA045	24.4	10.5	69.8	143.5	31.9	17.0	15.0	24.9	23.0	25.9	22.4	675.9	156.5	1.5	98.5	408.4	16.2	189.7	27.1	486.7	17531.0	2.0	1.9	-17.9	63.6	1671.2
RDA046	23.7	9.8	67.0	194.2	31.2	16.5	14.7	25.1	22.6	25.5	21.0	479.8	111.3	3.2	86.8	265.2	14.5	127.8	38.2	444.2	18154.5	2.2	2.0	-15.3	67.7	1580.4
RDA047	24.9	11.1	68.7	184.9	33.1	16.9	16.2	26.5	23.9	26.7	22.3	383.9	98.3	1.7	94.1	218.9	9.1	124.9	19.4	362.2	17870.1	2.2	2.0	-13.7	64.8	1648.5
RDA048	26.2	8.7	75.9	106.9	31.8	20.3	11.5	26.0	26.7	27.3	24.7	1372.2	236.3	13.3	71.4	657.0	66.1	335.5	500.6	30.6	18858.0	2.7	3.1	-10.5	77.8	1569.4
RDA049	26.4	8.4	74.7	106.2	31.8	20.6	11.3	26.2	26.9	27.5	24.9	1043.0	177.0	7.8	75.9	508.5	37.1	289.3	357.6	23.6	18734.8	2.7	3.1	-10.9	77.4	1565.7
RDA050	26.4	8.4	74.7	106.2	31.8	20.6	11.3	26.2	26.9	27.5	24.9	1043.0	177.0	7.8	75.9	508.5	37.1	289.3	357.6	23.6	18734.8	2.7	3.1	-10.9	77.4	1565.7
RDA051	27.4	9.0	76.3	100.0	33.1	21.4	11.7	28.0	27.6	28.3	25.9	592.7	157.8	0.9	110.6	387.5	6.1	138.7	62.8	40.8	18434.4	2.6	2.2	-10.0	69.2	1651.4
RDA052	26.2	8.7	75.9	106.9	31.8	20.3	11.5	26.0	26.7	27.3	24.7	1372.2	236.3	13.3	71.4	657.0	66.1	335.5	500.6	30.6	18858.0	2.7	3.1	-10.5	77.8	1569.4
RDA053	24.8	10.2	75.5	100.2	31.8	18.3	13.5	24.7	24.9	26.0	23.5	844.3	221.5	3.7	102.3	514.0	15.5	50.9	195.0	544.6	17956.7	2.2	2.0	-20.5	63.7	1667.8
RDA054	24.8	10.2	75.5	100.2	31.8	18.3	13.5	24.7	24.9	26.0	23.5	844.3	221.5	3.7	102.3	514.0	15.5	50.9	195.0	544.6	17956.7	2.2	2.0	-20.5	63.7	1667.8
RDA055	24.1	9.4	74.7	98.4	30.5	18.0	12.6	24.6	24.4	25.2	22.7	592.5	149.4	2.3	107.7	383.1	8.6	71.4	57.2	589.6	18685.9	2.2	2.0	-15.9	67.2	1572.3
RDA056	24.1	9.4	74.7	98.4	30.5	18.0	12.6	24.6	24.4	25.2	22.7	592.5	149.4	2.3	107.7	383.1	8.6	71.4	57.2	589.6	18685.9	2.2	2.0	-15.9	67.2	1572.3
RDA057	27.0	9.4	75.5	109.1	33.6	21.1	12.5	27.5	26.5	28.1	25.4	686.2	196.0	1.4	113.1	468.1	6.9	90.6	49.5	205.5	18578.8	2.4	1.9	-12.5	63.6	1678.1
RDA058	27.0	9.4	75.5	109.1	33.6	21.1	12.5	27.5	26.5	28.1	25.4	686.2	196.0	1.4	113.1	468.1	6.9	90.6	49.5	205.5	18578.8	2.4	1.9	-12.5	63.6	1678.1
RDA059	21.4	11.3	65.6	124.4	29.5	12.3	17.3	21.8	19.8	22.6	19.6	1501.9	285.8	2.4	85.6	761.9	12.6	411.2	29.5	1083.6	16437.7	1.8	1.8	-38.1	68.4	1551.9
RDA060	23.4	13.0	71.0	181.5	31.7	13.4	18.2	24.6	20.8	25.0	20.8	770.9	166.2	2.2	92.4	437.3	9.5	280.9	9.5	474.3	15691.4	2.1	1.3	-25.4	70.7	1748.8
RDA061	21.9	11.2	66.0	122.0	29.8	12.8	17.0	22.3	20.3	23.0	20.2	1423.5	268.9	1.5	86.7	728.4	9.9	381.1	26.1	1046.1	16563.5	1.8	1.8	-36.6	68.8	1540.9
RDA062	23.4	13.0	71.0	181.5	31.7	13.4	18.2	24.6	20.8	25.0	20.8	770.9	166.2	2.2	92.4	437.3	9.5	280.9	9.5	474.3	15691.4	2.1	1.3	-25.4	70.7	1748.8
RDA063	18.2	7.7	40.5	415.9	27.8	8.8	18.9	15.5	21.9	23.2	13.0	1225.3	135.4	66.5	21.6	387.7	235.6	295.9	325.1	2.5	15069.9	1.7	3.4	-38.1	80.2	1152.6
RDA064	27.0	12.4	72.1	114.7	36.1	19.0	17.1	26.3	26.5	28.7	26.1	850.3	146.6	1.7	83.6	406.6	12.0	175.8	29.9	310.0	17681.3	2.2	0.9	-17.2	63.8	1911.2
RDA065	27.1	11.9	70.7	118.6	36.0	19.3	16.8	26.4	26.6	28.9	26.1	876.7	147.5	1.0	83.6	423.5	7.9	175.0	32.3	236.2	17501.1	2.3	0.8	-18.4	65.4	1891.0
RDA066	27.1	11.9	70.7	118.6	36.0	19.3	16.8	26.4	26.6	28.9	26.1	876.7	147.5	1.0	83.6	423.5	7.9	175.0	32.3	236.2	17501.1	2.3	0.8	-18.4	65.4	1891.0
RDA067	25.8	11.6	66.7	116.5	34.0	16.7	17.4	26.1	24.4	27.1	24.3	1279.6	242.3	1.6	88.4	670.5	7.5	384.8	16.2	446.1	16677.8	2.1	1.4	-25.5	68.0	1673.5
RDA068	23.3	11.9	66.8	142.5	31.4	13.6	17.8	23.9	21.4	24.6	21.3	1279.0	268.8	1.4	90.9	695.1	8.1	349.6	23.3	701.9	16677.3	2.0	1.5	-31.3	69.8	1674.1

	WorldClim (2007-2018)																			WorldClim (1970-2000)					CRU (1901-2019)	
**ID**	**Bio1**	**Bio2**	**Bio3**	**Bio4**	**Bio5**	**Bio6**	**Bio7**	**Bio8**	**Bio9**	**Bio10**	**Bio11**	**Bio12**	**Bio13**	**Bio14**	**Bio15**	**Bio16**	**Bio17**	**Bio18**	**Bio19**	**Altitude**	**SolarRad**	**Vapor**	**Wind**	**δ^2H_p**	**Hum**	**Evapo**
RDA069	22.8	12.7	68.6	191.4	30.6	12.1	18.6	24.1	20.2	24.4	20.1	1095.5	238.8	3.7	89.0	606.3	15.8	462.2	37.1	652.4	16071.4	2.0	1.1	-34.5	74.2	1722.2
RDA070	25.6	10.5	70.6	82.8	32.8	17.9	14.9	25.9	24.6	26.4	24.4	1601.5	246.0	50.4	44.6	644.9	197.3	346.7	232.8	181.2	13938.5	2.7	1.2	-56.3	83.6	1732.9
RDA071	21.6	11.2	65.9	116.4	29.6	12.7	16.9	22.0	20.2	22.8	20.0	1474.8	263.4	2.4	83.6	736.3	13.5	412.2	32.4	1094.3	16416.3	1.8	1.8	-37.9	68.3	1522.5
RDA072	25.4	6.4	64.4	126.5	30.4	20.5	9.9	25.2	26.4	26.8	23.8	1484.5	302.1	54.6	57.7	680.6	213.8	252.8	419.4	17.3	17552.6	2.6	2.5	-5.2	82.1	1348.4
RDA073	25.4	6.4	64.4	126.5	30.4	20.5	9.9	25.2	26.4	26.8	23.8	1484.5	302.1	54.6	57.7	680.6	213.8	252.8	419.4	17.3	17552.6	2.6	2.5	-5.2	82.1	1348.4
RDA074	21.6	11.2	65.9	116.8	29.7	12.7	17.0	22.0	20.2	22.8	20.0	1475.4	264.0	2.4	83.6	736.7	13.6	412.5	32.5	1107.2	16431.2	1.8	1.8	-37.9	68.3	1517.7
RDA075	20.9	9.9	62.1	222.5	28.0	12.0	16.0	23.3	18.9	23.3	18.0	1396.9	274.8	30.9	70.0	689.2	119.1	689.2	130.4	645.8	15089.6	1.8	1.8	-35.5	76.1	1430.7
RDA076	20.9	9.9	62.1	222.5	28.0	12.0	16.0	23.3	18.9	23.3	18.0	1396.9	274.8	30.9	70.0	689.2	119.1	689.2	130.4	645.8	15089.6	1.8	1.8	-35.5	76.1	1430.7
RDA077	21.4	10.2	63.1	221.9	28.6	12.3	16.2	23.7	19.4	23.7	18.4	1398.6	277.0	30.9	70.8	695.9	115.8	669.0	126.6	647.7	14830.0	1.8	1.8	-34.8	75.6	1450.5
RDA078	21.4	10.2	63.1	221.9	28.6	12.3	16.2	23.7	19.4	23.7	18.4	1398.6	277.0	30.9	70.8	695.9	115.8	669.0	126.6	647.7	14830.0	1.8	1.8	-34.8	75.6	1450.5
RDA079	28.0	8.9	72.8	73.9	34.6	22.4	12.3	27.2	28.3	29.0	27.2	2241.8	322.2	37.9	57.5	941.1	149.4	300.9	941.1	75.8	14584.7	2.8	1.0	-37.8	87.9	1748.3
RDA080	27.9	8.9	73.4	75.6	34.4	22.3	12.2	27.0	28.2	28.9	27.0	2262.3	319.4	40.9	56.0	931.7	163.6	303.6	931.7	87.0	14428.0	2.8	1.0	-38.1	87.8	1742.5
RDA081	27.9	8.9	73.4	75.6	34.4	22.3	12.2	27.0	28.2	28.9	27.0	2262.3	319.4	40.9	56.0	931.7	163.6	303.6	931.7	87.0	14428.0	2.8	1.0	-38.1	87.8	1742.5
RDA082	28.0	8.9	71.9	72.5	34.7	22.3	12.4	27.2	28.3	29.0	27.2	2214.9	333.2	32.1	60.6	968.0	127.6	297.2	918.2	96.3	14463.1	2.8	1.0	-38.4	88.0	1748.0
RDA083	25.6	13.5	78.3	99.2	34.0	16.7	17.3	25.7	24.8	26.7	24.2	659.8	134.2	1.1	91.6	357.5	10.0	95.1	10.9	601.8	17862.9	1.9	1.1	-18.9	60.0	1977.0
RDA084	25.9	13.6	78.2	100.8	34.4	17.0	17.4	26.1	25.2	27.0	24.5	666.1	133.7	1.2	91.0	357.4	10.3	96.9	11.1	544.8	17814.5	2.0	1.1	-18.2	60.2	2000.9
RDA085	26.6	9.9	83.1	61.9	32.8	20.9	11.9	25.9	26.9	27.5	25.9	1667.5	334.4	21.3	81.6	888.6	82.0	144.2	832.0	141.7	15769.8	2.7	1.2	-23.3	80.8	1755.8
RDA086	26.6	9.9	83.1	61.9	32.8	20.9	11.9	25.9	26.9	27.5	25.9	1667.5	334.4	21.3	81.6	888.6	82.0	144.2	832.0	141.7	15769.8	2.7	1.2	-23.3	80.8	1755.8
RDA087	26.6	9.7	87.6	42.3	32.2	21.2	11.0	26.7	26.8	27.0	26.0	2913.9	372.7	153.3	26.6	959.7	496.7	515.8	646.4	98.9	14148.9	2.9	1.1	-31.4	88.1	1584.3
RDA088	26.6	9.7	87.6	42.3	32.2	21.2	11.0	26.7	26.8	27.0	26.0	2913.9	372.7	153.3	26.6	959.7	496.7	515.8	646.4	98.9	14148.9	2.9	1.1	-31.4	88.1	1584.3
RDA089	27.1	11.5	71.9	74.8	35.8	19.8	16.0	26.2	27.4	28.0	26.2	1742.8	321.0	8.9	73.9	836.6	52.1	202.3	836.6	138.4	15955.9	2.7	1.4	-22.3	82.5	1862.2
RDA090	27.1	11.5	71.9	74.8	35.8	19.8	16.0	26.2	27.4	28.0	26.2	1742.8	321.0	8.9	73.9	836.6	52.1	202.3	836.6	138.4	15955.9	2.7	1.4	-22.3	82.5	1862.2
RDA091	26.4	11.3	66.9	73.3	35.7	18.8	16.9	25.6	26.7	27.4	25.6	2257.0	353.5	6.5	73.3	1032.5	39.5	364.5	1032.5	411.3	14815.8	2.5	1.5	-48.9	83.4	1949.0
RDA092	26.6	11.3	66.2	76.3	36.1	19.0	17.1	25.8	27.0	27.7	25.8	2242.5	347.5	7.0	72.5	1015.0	41.0	369.2	1015.0	359.1	14775.2	2.5	1.5	-48.3	83.6	1958.6
RDA093	24.5	7.8	66.4	146.3	30.3	18.6	11.8	24.2	24.3	26.0	22.5	1196.0	254.9	46.2	58.6	536.5	169.8	220.0	339.2	151.6	17458.0	2.5	1.9	-6.5	82.1	1443.9
RDA094	23.6	9.9	70.2	160.7	30.4	16.3	14.1	25.1	21.6	25.2	21.4	583.5	79.2	10.3	48.5	229.2	57.1	227.5	81.6	267.5	16817.8	2.2	1.7	-9.2	74.0	1690.5
RDA095	23.6	9.9	70.2	160.7	30.4	16.3	14.1	25.1	21.6	25.2	21.4	583.5	79.2	10.3	48.5	229.2	57.1	227.5	81.6	267.5	16817.8	2.2	1.7	-9.2	74.0	1690.5
RDA096	24.5	7.8	66.4	146.3	30.3	18.6	11.8	24.2	24.3	26.0	22.5	1196.0	254.9	46.2	58.6	536.5	169.8	220.0	339.2	151.6	17458.0	2.5	1.9	-6.5	82.1	1443.9
RDA097	27.1	10.8	65.9	78.9	36.4	20.0	16.3	26.2	27.7	28.1	26.2	2264.2	343.6	9.9	67.5	993.9	64.4	168.9	947.2	253.0	14477.4	2.6	1.3	-42.6	86.4	1919.4
RDA098	26.3	13.0	68.6	69.7	35.8	16.9	19.0	26.0	25.8	27.2	25.7	2017.0	355.9	1.2	80.3	980.1	18.8	276.6	52.8	294.2	15197.4	2.5	1.4	-47.3	81.1	1972.0
RDA099	23.9	7.6	63.0	159.3	29.6	17.5	12.1	24.6	22.5	25.6	21.8	1160.3	143.1	63.5	24.6	371.3	243.9	274.5	262.8	176.7	16446.3	2.4	1.5	-8.3	82.1	1381.5
RDA100	23.9	7.6	63.0	159.3	29.6	17.5	12.1	24.6	22.5	25.6	21.8	1160.3	143.1	63.5	24.6	371.3	243.9	274.5	262.8	176.7	16446.3	2.4	1.5	-8.3	82.1	1381.5
RDA101	24.4	6.1	62.3	135.0	29.2	19.4	9.9	24.1	23.5	26.0	22.6	1671.5	180.7	84.4	23.3	529.7	313.0	400.1	470.7	117.4	16842.4	2.5	2.2	-5.0	82.5	1266.4
RDA102	27.0	9.7	81.1	62.9	33.4	21.5	11.9	26.2	27.6	27.8	26.2	2807.2	450.2	54.5	64.5	1283.9	186.0	216.6	1139.8	22.2	15514.5	2.9	1.8	-21.6	86.2	1784.9
RDA103	27.0	9.7	81.1	62.9	33.4	21.5	11.9	26.2	27.6	27.8	26.2	2807.2	450.2	54.5	64.5	1283.9	186.0	216.6	1139.8	22.2	15514.5	2.9	1.8	-21.6	86.2	1784.9

	WorldClim (2007-2018)																			WorldClim (1970-2000)					CRU (1901-2019)	
**ID**	**Bio1**	**Bio2**	**Bio3**	**Bio4**	**Bio5**	**Bio6**	**Bio7**	**Bio8**	**Bio9**	**Bio10**	**Bio11**	**Bio12**	**Bio13**	**Bio14**	**Bio15**	**Bio16**	**Bio17**	**Bio18**	**Bio19**	**Altitude**	**SolarRad**	**Vapor**	**Wind**	**δ^2H_p**	**Hum**	**Evapo**
RDA104	27.1	9.4	82.8	67.7	33.2	21.9	11.3	26.3	27.8	28.0	26.3	2323.4	472.2	38.0	79.2	1228.6	149.8	177.2	1228.6	87.3	15924.4	2.8	1.4	-22.1	83.9	1736.2
RDA105	27.1	9.4	82.8	67.7	33.2	21.9	11.3	26.3	27.8	28.0	26.3	2323.4	472.2	38.0	79.2	1228.6	149.8	177.2	1228.6	87.3	15924.4	2.8	1.4	-22.1	83.9	1736.2
RDA106	26.9	9.5	79.9	69.9	33.4	21.5	11.9	26.4	27.8	27.8	26.1	2197.5	332.0	41.9	61.8	987.9	140.0	165.2	809.2	29.1	15484.3	2.9	1.7	-27.0	85.9	1721.2
RDA107	26.9	9.5	79.9	69.9	33.4	21.5	11.9	26.4	27.8	27.8	26.1	2197.5	332.0	41.9	61.8	987.9	140.0	165.2	809.2	29.1	15484.3	2.9	1.7	-27.0	85.9	1721.2
RDA108	26.3	8.8	75.3	68.2	33.2	21.6	11.6	26.0	27.2	27.2	25.6	3221.8	477.3	56.9	55.8	1281.9	227.8	241.1	1143.9	40.0	17836.9	2.8	1.5	-7.9	83.8	1648.5
RDA109	26.3	8.8	75.3	68.2	33.2	21.6	11.6	26.0	27.2	27.2	25.6	3221.8	477.3	56.9	55.8	1281.9	227.8	241.1	1143.9	40.0	17836.9	2.8	1.5	-7.9	83.8	1648.5
RDA110	27.0	9.6	80.6	64.4	33.5	21.6	11.9	26.3	27.7	27.9	26.3	2506.5	388.4	63.5	58.2	1080.5	201.0	217.1	990.7	8.2	15627.2	2.9	1.8	-22.4	86.1	1783.1
RDA111	27.6	8.3	74.4	80.9	33.6	22.5	11.2	26.6	28.7	28.7	26.6	3027.4	515.6	32.4	73.0	1517.5	110.5	110.5	1517.5	9.2	15778.9	2.9	1.6	-21.8	84.6	1636.1
RDA112	26.8	11.9	71.6	78.4	35.9	19.3	16.6	25.9	27.2	27.9	25.9	1819.1	316.5	7.0	73.5	849.0	41.9	212.4	849.0	234.5	15326.3	2.6	1.5	-23.8	83.8	1877.9
RDA113	26.7	11.9	71.7	76.8	35.8	19.2	16.6	25.8	27.1	27.8	25.8	1809.7	312.2	6.9	74.0	844.0	38.4	208.7	844.0	272.7	15488.0	2.6	1.5	-23.9	84.0	1875.7
RDA114	26.3	12.2	67.9	67.9	35.8	17.9	17.9	25.7	26.3	27.3	25.7	2203.9	375.9	4.0	76.2	1044.8	31.5	334.6	1031.2	328.0	14960.9	2.5	1.4	-48.8	82.4	1968.5
RDA115	26.6	12.4	68.9	63.5	35.9	17.9	18.0	26.1	26.4	27.5	26.1	2267.4	392.5	3.0	77.8	1091.7	24.5	365.2	1045.1	309.7	15320.8	2.5	1.4	-45.7	82.0	1966.3
RDA116	25.1	13.9	71.5	84.1	34.2	14.8	19.4	25.2	24.1	26.0	24.1	2022.8	382.5	1.3	87.8	1069.0	25.3	448.0	47.6	335.7	14899.9	2.4	1.3	-49.3	78.4	1921.1
RDA117	26.9	10.5	73.4	55.5	34.5	20.2	14.3	26.4	27.1	27.7	26.3	2552.2	379.9	23.2	61.5	1103.1	112.5	402.1	1061.7	72.7	14039.4	2.8	0.8	-54.6	85.6	1740.6
RDA118	26.9	10.5	73.4	55.5	34.5	20.2	14.3	26.4	27.1	27.7	26.3	2552.2	379.9	23.2	61.5	1103.1	112.5	402.1	1061.7	72.7	14039.4	2.8	0.8	-54.6	85.6	1740.6
RDA119	18.9	7.4	39.5	415.3	28.3	9.5	18.8	16.2	22.5	23.8	13.7	1247.6	143.2	59.8	24.7	414.1	222.1	317.9	334.7	4.0	14763.2	1.7	3.6	-38.7	81.1	1127.0
RDA120	19.4	9.2	45.0	431.3	29.4	9.1	20.3	16.7	23.8	24.1	13.9	1504.4	163.2	91.0	20.3	482.0	306.5	355.4	408.5	172.7	14744.5	1.7	2.4	-40.7	77.8	1273.6
RDA121	19.0	7.3	38.9	418.1	28.3	9.6	18.8	16.2	22.6	23.9	13.7	1250.2	144.6	60.4	24.9	416.1	222.7	318.6	333.5	4.3	14661.7	1.7	3.8	-38.7	81.1	1124.0
RDA122	20.4	8.6	46.1	370.4	29.8	11.1	18.7	18.2	18.2	24.7	15.7	1640.1	181.1	92.0	19.5	476.6	352.7	449.1	383.2	30.6	14129.3	1.8	3.1	-38.0	77.4	1273.5
RDA124	20.5	8.4	45.5	369.1	29.6	11.2	18.4	18.3	18.3	24.8	15.7	1562.8	170.8	91.4	19.0	450.2	333.2	429.8	356.7	4.9	14725.6	1.8	3.3	-37.1	77.2	1281.5
RDA125	19.5	11.2	65.1	202.2	26.4	9.2	17.1	21.2	16.7	21.3	16.7	1668.2	322.2	15.5	76.7	831.6	61.5	707.1	69.5	1168.1	15197.2	1.7	1.9	-41.7	76.4	1448.5
RDA126	17.8	10.4	52.7	347.8	27.6	7.8	19.8	16.1	15.4	22.0	13.5	1810.0	226.7	108.8	21.4	574.9	381.8	461.5	441.1	607.0	14515.9	1.6	2.2	-46.4	77.2	1273.2
RDA127	20.1	11.1	46.0	474.8	32.2	8.1	24.1	25.8	14.1	25.8	14.1	1445.3	189.1	47.9	34.0	488.7	205.6	488.7	205.6	57.0	16518.7	1.7	3.3	-43.7	73.2	1453.0
RDA128	23.4	6.8	53.9	197.8	29.9	17.4	12.5	25.9	21.3	25.9	21.0	1125.4	155.2	29.0	47.7	440.6	109.6	440.6	124.4	12.0	16180.4	2.3	3.3	-30.6	82.0	1155.6
RDA129	24.1	9.1	59.8	199.1	31.4	16.2	15.2	24.7	21.9	26.5	21.6	1116.2	192.7	39.7	45.8	440.0	129.9	296.4	165.0	14.4	15420.3	2.4	2.6	-27.1	78.5	1400.8
RDA130	24.1	9.1	59.8	199.1	31.4	16.2	15.2	24.7	21.9	26.5	21.6	1116.2	192.7	39.7	45.8	440.0	129.9	296.4	165.0	14.4	15420.3	2.4	2.6	-27.1	78.5	1400.8
RDA131	24.1	11.9	60.3	290.2	32.8	13.1	19.7	26.9	20.2	26.9	20.1	1429.1	214.4	31.4	47.8	576.0	152.5	576.0	234.1	300.3	16150.7	2.1	2.7	-50.1	72.9	1674.1
RDA132	24.1	11.9	60.3	290.2	32.8	13.1	19.7	26.9	20.2	26.9	20.1	1429.1	214.4	31.4	47.8	576.0	152.5	576.0	234.1	300.3	16150.7	2.1	2.7	-50.1	72.9	1674.1
RDA133	25.7	11.3	62.1	250.3	33.6	15.5	18.1	27.7	22.2	27.8	22.1	1266.7	239.7	24.6	65.8	624.8	104.4	394.3	160.1	111.5	15827.5	2.3	2.2	-44.9	73.9	1732.5
RDA134	25.7	11.3	62.1	250.3	33.6	15.5	18.1	27.7	22.2	27.8	22.1	1266.7	239.7	24.6	65.8	624.8	104.4	394.3	160.1	111.5	15827.5	2.3	2.2	-44.9	73.9	1732.5
RDA135	25.7	11.3	62.1	250.3	33.6	15.5	18.1	27.7	22.2	27.8	22.1	1266.7	239.7	24.6	65.8	624.8	104.4	394.3	160.1	111.5	15827.5	2.3	2.2	-44.9	73.9	1732.5
RDA136	25.7	11.3	62.1	250.3	33.6	15.5	18.1	27.7	22.2	27.8	22.1	1266.7	239.7	24.6	65.8	624.8	104.4	394.3	160.1	111.5	15827.5	2.3	2.2	-44.9	73.9	1732.5
RDA137	21.2	12.3	67.9	185.4	29.1	11.1	18.1	22.4	18.4	23.0	18.4	1005.3	222.1	4.8	88.1	546.3	17.5	358.5	17.5	841.8	15400.6	1.9	1.2	-36.5	78.6	1602.3
RDA138	21.2	12.3	67.9	185.4	29.1	11.1	18.1	22.4	18.4	23.0	18.4	1005.3	222.1	4.8	88.1	546.3	17.5	358.5	17.5	841.8	15400.6	1.9	1.2	-36.5	78.6	1602.3
RDA139	21.2	12.3	67.9	185.4	29.1	11.1	18.1	22.4	18.4	23.0	18.4	1005.3	222.1	4.8	88.1	546.3	17.5	358.5	17.5	841.8	15400.6	1.9	1.2	-36.5	78.6	1602.3

	WorldClim (2007-2018)																			WorldClim (1970-2000)					CRU (1901-2019)	
**ID**	**Bio1**	**Bio2**	**Bio3**	**Bio4**	**Bio5**	**Bio6**	**Bio7**	**Bio8**	**Bio9**	**Bio10**	**Bio11**	**Bio12**	**Bio13**	**Bio14**	**Bio15**	**Bio16**	**Bio17**	**Bio18**	**Bio19**	**Altitude**	**SolarRad**	**Vapor**	**Wind**	**δ^2H_p**	**Hum**	**Evapo**
RDA140	21.2	12.3	67.9	185.4	29.1	11.1	18.1	22.4	18.4	23.0	18.4	1005.3	222.1	4.8	88.1	546.3	17.5	358.5	17.5	841.8	15400.6	1.9	1.2	-36.5	78.6	1602.3
RDA141	23.8	10.9	66.2	123.0	31.8	15.3	16.5	24.0	22.5	25.2	22.1	1623.5	309.2	3.4	85.4	842.9	17.0	434.0	38.3	803.1	16196.0	2.0	1.8	-33.6	67.3	1608.8
RDA142	23.8	10.9	66.2	123.0	31.8	15.3	16.5	24.0	22.5	25.2	22.1	1623.5	309.2	3.4	85.4	842.9	17.0	434.0	38.3	803.1	16196.0	2.0	1.8	-33.6	67.3	1608.8
RDA143	23.8	10.9	66.2	123.0	31.8	15.3	16.5	24.0	22.5	25.2	22.1	1623.5	309.2	3.4	85.4	842.9	17.0	434.0	38.3	803.1	16196.0	2.0	1.8	-33.6	67.3	1608.8
RDA144	23.8	10.9	66.2	123.0	31.8	15.3	16.5	24.0	22.5	25.2	22.1	1623.5	309.2	3.4	85.4	842.9	17.0	434.0	38.3	803.1	16196.0	2.0	1.8	-33.6	67.3	1608.8
RDA145	25.9	11.9	63.0	192.7	34.1	15.3	18.8	27.1	23.1	27.4	23.1	1354.3	273.0	21.0	78.1	693.4	69.0	442.3	78.8	119.1	15287.0	2.4	1.6	-45.7	75.4	1790.0
RDA146	25.9	11.9	63.0	192.7	34.1	15.3	18.8	27.1	23.1	27.4	23.1	1354.3	273.0	21.0	78.1	693.4	69.0	442.3	78.8	119.1	15287.0	2.4	1.6	-45.7	75.4	1790.0
RDA147	25.8	11.8	62.9	192.7	34.1	15.3	18.8	27.1	23.1	27.4	23.1	1348.9	272.0	21.0	78.2	692.0	68.6	440.9	78.8	120.7	15270.9	2.4	1.6	-45.7	75.4	1790.0
RDA148	25.9	12.0	63.3	192.5	34.2	15.2	19.0	27.2	23.1	27.4	23.1	1361.4	273.9	21.4	77.9	695.9	69.3	444.5	88.0	120.1	15287.3	2.4	1.6	-45.8	75.5	1789.2
RDA149	25.9	12.0	63.3	192.5	34.2	15.2	19.0	27.2	23.1	27.4	23.1	1361.4	273.9	21.4	77.9	695.9	69.3	444.5	88.0	120.1	15287.3	2.4	1.6	-45.8	75.5	1789.2
RDA150	21.4	11.4	65.6	125.2	29.6	12.2	17.4	21.9	19.8	22.7	19.7	1489.4	284.5	2.3	85.9	758.1	12.0	407.0	28.8	1063.2	16504.2	1.8	1.8	-37.9	68.5	1558.3
RDA151	21.4	11.4	65.6	125.2	29.6	12.2	17.4	21.9	19.8	22.7	19.7	1489.4	284.5	2.3	85.9	758.1	12.0	407.0	28.8	1063.2	16504.2	1.8	1.8	-37.9	68.5	1558.3
RDA152	22.0	10.1	64.8	110.4	29.8	14.2	15.6	22.1	20.9	23.3	20.6	1600.8	282.7	3.5	84.3	813.6	13.6	453.6	34.9	1135.4	16344.6	1.8	1.9	-39.2	69.0	1464.9
RDA153	22.0	10.1	64.8	110.4	29.8	14.2	15.6	22.1	20.9	23.3	20.6	1600.8	282.7	3.5	84.3	813.6	13.6	453.6	34.9	1135.4	16344.6	1.8	1.9	-39.2	69.0	1464.9
RDA154	22.0	10.1	64.8	110.4	29.8	14.2	15.6	22.1	20.9	23.3	20.6	1600.8	282.7	3.5	84.3	813.6	13.6	453.6	34.9	1135.4	16344.6	1.8	1.9	-39.2	69.0	1464.9
RDA155	22.0	10.1	64.8	110.4	29.8	14.2	15.6	22.1	20.9	23.3	20.6	1600.8	282.7	3.5	84.3	813.6	13.6	453.6	34.9	1135.4	16344.6	1.8	1.9	-39.2	69.0	1464.9
RDA156	23.0	12.4	68.9	136.6	30.9	12.9	18.0	23.9	21.1	24.0	21.0	1642.5	326.9	18.5	80.5	834.1	63.5	360.6	81.7	635.3	15227.5	2.1	1.5	-53.8	73.0	1684.2
RDA157	23.7	12.6	69.4	134.8	31.7	13.6	18.1	24.6	21.8	24.7	21.8	1601.4	312.5	19.6	78.8	805.1	69.2	634.0	85.5	656.9	15101.7	2.1	1.5	-53.0	73.0	1675.5
RDA158	23.7	12.6	69.4	134.8	31.7	13.6	18.1	24.6	21.8	24.7	21.8	1601.4	312.5	19.6	78.8	805.1	69.2	634.0	85.5	656.9	15101.7	2.1	1.5	-53.0	73.0	1675.5
RDA159	23.4	12.3	68.8	136.7	31.3	13.3	17.9	24.2	21.4	24.3	21.4	1619.6	319.0	19.4	79.6	818.8	65.8	355.0	84.4	614.0	15169.9	2.1	1.5	-52.8	73.0	1685.1
RDA160	22.2	10.9	67.5	99.8	30.1	13.9	16.2	22.3	21.2	23.4	21.0	1567.5	279.5	2.7	86.6	799.3	10.1	446.5	27.4	1139.6	16600.2	1.8	1.8	-37.1	68.6	1519.8
RDA161	22.2	10.9	67.5	99.8	30.1	13.9	16.2	22.3	21.2	23.4	21.0	1567.5	279.5	2.7	86.6	799.3	10.1	446.5	27.4	1139.6	16600.2	1.8	1.8	-37.1	68.6	1519.8
RDA162	22.2	10.9	67.4	99.6	30.1	13.9	16.1	22.3	21.2	23.4	21.0	1566.5	279.3	2.7	86.6	799.0	10.3	446.0	27.4	1059.0	16571.2	1.8	1.7	-37.1	68.6	1534.6
RDA163	22.2	10.9	67.4	99.6	30.1	13.9	16.1	22.3	21.2	23.4	21.0	1566.5	279.3	2.7	86.6	799.0	10.3	446.0	27.4	1059.0	16571.2	1.8	1.7	-37.1	68.6	1534.6
RDA164	26.0	12.3	71.2	88.5	35.0	17.7	17.3	25.2	26.0	27.3	25.2	1294.5	225.8	6.5	77.5	636.8	28.9	128.9	636.8	346.3	15960.2	2.3	1.1	-22.9	72.8	1806.5
RDA165	26.0	12.3	71.2	88.5	35.0	17.7	17.3	25.2	26.0	27.3	25.2	1294.5	225.8	6.5	77.5	636.8	28.9	128.9	636.8	346.3	15960.2	2.3	1.1	-22.9	72.8	1806.5
RDA166	26.1	12.0	70.7	87.0	35.1	18.1	17.0	25.2	26.3	27.4	25.2	1477.7	258.4	8.9	76.4	732.0	41.1	142.3	732.0	358.6	15755.9	2.4	1.2	-22.6	73.3	1781.6
RDA167	26.1	12.0	70.7	87.0	35.1	18.1	17.0	25.2	26.3	27.4	25.2	1477.7	258.4	8.9	76.4	732.0	41.1	142.3	732.0	358.6	15755.9	2.4	1.2	-22.6	73.3	1781.6
RDA168	27.3	8.5	76.8	81.3	33.4	22.3	11.1	26.3	28.3	28.3	26.3	2437.8	371.0	36.2	62.6	1072.8	118.1	118.1	1010.0	17.3	16367.8	2.9	1.7	-21.5	83.7	1582.1
RDA169	27.3	8.5	76.8	81.3	33.4	22.3	11.1	26.3	28.3	28.3	26.3	2437.8	371.0	36.2	62.6	1072.8	118.1	118.1	1010.0	17.3	16367.8	2.9	1.7	-21.5	83.7	1582.1
RDA170	27.6	7.6	78.7	65.8	32.9	23.2	9.7	27.1	28.3	28.5	26.9	2321.7	321.9	88.9	40.0	884.6	304.9	369.8	847.0	52.1	15225.1	2.9	1.2	-36.7	81.2	1654.4
RDA171	27.7	7.6	77.5	70.5	33.0	23.3	9.8	27.2	28.2	28.7	27.0	2301.4	319.9	80.8	42.9	879.7	277.3	363.5	871.7	50.0	15738.9	2.9	1.2	-36.8	81.4	1646.4
RDA172	25.5	6.3	64.5	124.2	30.3	20.6	9.7	25.3	26.4	26.9	23.9	1528.1	303.3	58.4	56.7	697.3	220.6	263.6	430.2	13.3	17605.4	2.6	2.5	-5.5	82.1	1321.9
RDA173	25.5	6.3	64.5	124.2	30.3	20.6	9.7	25.3	26.4	26.9	23.9	1528.1	303.3	58.4	56.7	697.3	220.6	263.6	430.2	13.3	17605.4	2.6	2.5	-5.5	82.1	1321.9
RDA174	24.8	8.9	64.9	199.3	31.8	18.0	13.8	22.7	25.1	26.7	22.0	501.9	95.0	9.8	65.7	247.6	40.5	97.7	186.0	323.6	17842.5	2.3	1.9	-10.9	71.9	1529.0

	WorldClim (2007-2018)																			WorldClim (1970-2000)					CRU (1901-2019)	
**ID**	**Bio1**	**Bio2**	**Bio3**	**Bio4**	**Bio5**	**Bio6**	**Bio7**	**Bio8**	**Bio9**	**Bio10**	**Bio11**	**Bio12**	**Bio13**	**Bio14**	**Bio15**	**Bio16**	**Bio17**	**Bio18**	**Bio19**	**Altitude**	**SolarRad**	**Vapor**	**Wind**	**δ^2H_p**	**Hum**	**Evapo**
RDA175	25.2	9.0	66.2	192.4	32.1	18.5	13.7	23.2	25.6	27.0	22.5	478.0	79.6	7.2	56.7	201.5	34.8	125.8	143.8	234.8	17842.3	2.4	2.0	-10.6	69.8	1603.1
RDA176	27.2	8.4	78.4	76.1	33.1	22.4	10.7	26.5	28.2	28.3	26.5	1923.2	318.1	32.0	64.7	878.3	122.3	178.7	878.3	31.8	15406.8	2.9	1.3	-39.0	85.2	1674.5
RDA177	27.2	8.4	78.4	76.1	33.1	22.4	10.7	26.5	28.2	28.3	26.5	1923.2	318.1	32.0	64.7	878.3	122.3	178.7	878.3	31.8	15406.8	2.9	1.3	-39.0	85.2	1674.5
RDA178	27.2	8.4	78.4	76.1	33.1	22.4	10.7	26.5	28.2	28.3	26.5	1923.2	318.1	32.0	64.7	878.3	122.3	178.7	878.3	31.8	15406.8	2.9	1.3	-39.0	85.2	1674.5
RDA179	27.7	7.5	78.7	62.2	32.9	23.4	9.5	27.1	28.3	28.6	27.1	2252.9	307.5	54.8	51.4	894.7	202.6	350.2	875.6	20.6	15029.1	2.9	1.1	-37.6	84.2	1633.1
RDA180	27.8	7.4	77.9	63.8	32.9	23.4	9.5	27.1	28.3	28.6	27.1	2235.2	309.6	50.6	52.4	894.3	194.6	348.8	876.9	19.9	15054.0	2.9	1.1	-37.7	84.0	1629.5
RDA181	27.7	7.5	78.7	62.2	32.9	23.4	9.5	27.1	28.3	28.6	27.1	2252.9	307.5	54.8	51.4	894.7	202.6	350.2	875.6	20.6	15029.1	2.9	1.1	-37.6	84.2	1633.1
RDA182	24.7	9.2	86.7	44.8	30.4	19.8	10.6	24.2	24.7	25.3	24.2	2004.4	347.5	72.5	59.6	959.8	239.8	314.8	959.8	622.3	15810.9	2.5	1.1	-40.3	79.5	1645.5
RDA183	24.7	9.2	86.7	44.8	30.4	19.8	10.6	24.2	24.7	25.3	24.2	2004.4	347.5	72.5	59.6	959.8	239.8	314.8	959.8	622.3	15810.9	2.5	1.1	-40.3	79.5	1645.5
RDA184	27.9	7.7	75.2	77.7	33.5	23.3	10.2	27.0	28.6	28.9	27.0	2320.5	321.5	44.1	53.1	918.6	191.0	374.5	912.3	74.1	14447.2	2.9	1.1	-38.9	83.4	1602.5
RDA185	28.1	8.2	75.5	82.1	34.1	23.3	10.8	27.2	28.9	29.1	27.2	2434.5	325.8	57.8	48.9	932.3	232.0	259.2	929.9	65.2	14132.8	2.9	1.1	-38.8	83.3	1606.6
RDA186	28.1	8.1	75.5	82.0	34.1	23.3	10.8	27.2	28.9	29.1	27.2	2432.4	325.7	57.6	49.0	932.1	231.2	258.9	929.6	65.0	14134.7	2.9	1.1	-38.8	83.3	1606.6
RDA187	28.1	8.2	75.5	82.1	34.1	23.3	10.8	27.2	28.9	29.1	27.2	2434.5	325.8	57.8	48.9	932.3	232.0	259.2	929.9	65.2	14132.8	2.9	1.1	-38.8	83.3	1606.6
RDA188	27.7	7.8	75.5	77.9	33.4	23.0	10.4	26.8	28.4	28.7	26.8	2385.5	323.1	52.7	49.7	917.9	214.4	391.3	913.6	102.7	14509.7	2.9	1.2	-39.5	83.2	1603.9
RDA189	27.4	8.1	84.7	45.1	32.6	23.0	9.6	27.1	27.9	28.1	27.0	2399.0	344.1	98.5	38.7	872.2	322.4	382.2	816.2	39.9	14862.7	2.9	1.1	-36.4	85.2	1665.2
RDA190	27.3	9.5	90.4	31.1	32.8	22.2	10.6	26.9	27.6	27.6	26.9	2430.2	342.7	123.0	36.2	890.5	410.9	410.9	890.5	51.9	15000.0	2.9	1.1	-31.0	86.2	1728.0
RDA191	24.1	12.3	68.3	161.8	32.1	14.0	18.0	25.1	22.1	25.5	21.7	1452.0	274.9	9.8	82.6	740.0	33.1	356.1	57.9	533.2	15782.2	2.1	2.1	-35.3	73.9	1726.7
RDA192	24.1	12.3	68.3	161.8	32.1	14.0	18.0	25.1	22.1	25.5	21.7	1452.0	274.9	9.8	82.6	740.0	33.1	356.1	57.9	533.2	15782.2	2.1	2.1	-35.3	73.9	1726.7
RDA193	21.8	11.4	65.8	123.9	30.0	12.6	17.3	22.2	20.2	23.0	20.0	1490.6	279.2	2.3	85.2	754.3	12.4	409.1	29.7	1068.9	16453.2	1.8	1.8	-37.4	68.4	1541.4

WorldClim variables (2007-2018): Bio1: Annual mean temperature; Bio2: Mean diurnal range temperature; Bio3: Isotermality; Bio4: Temperature seasonality; Bio5: Maximum temperature at the warmest month; Bio6: Minimum temperature at the coldest month; Bio7: Temperature annual range; Bio8: Mean temperature at the wettest quarter; Bio9: Mean temperature at the driest quarter; Bio10: Mean temperature at the warmest quarter; Bio11: Mean temperature at the coldest quarter; Bio12: Mean annual precipitation; Bio13: Precipitation at the wettest month; Bio14: Precipitation at the driest month; Bio15: Precipitation seasonality; Bio16: Precipitation at the wettest quarter; Bio17: Precipitation at the driest quarter; Bio18: Precipitation at the warmest quarter; Bio19: Precipitation at the coldest quarter. WorldClim variables (1970-2000): Altitude; Mean annual solar radiation; Mean annual water vapor pressure; Mean annual wind speed. δ^2Hp: Precipitation isotopic values from February to April. Climatic Research Unit (CRU) variables (1901-2019): Humidity; Potential evapotranspiration.

Second Step - Selection of variables using Recursive Feature Elimination – RFE

1- Define the control using a random forest selection function

(This function generates a control object that can be used to specify the details of the feature selection algorithms used in this package)

```
library(caret)
control <- rfeControl(functions=rfFuncs, method="cv", number=10)
```

2- Run the RFE algorithm (Backwards Feature Selection) = RFE (Recursive Feature Elimination)

rfe(predictores, observed values,) -> select the dataset to be used

```
predictor<-data.frame(Bio1_xy,Bio2_xy,Bio3_xy,Bio4_xy,Bio5_xy,Bio6_xy,Bio7_xy,Bio8_xy,Bio9_xy,Bio1
0_xy,Bio11_xy,Bio12_xy,Bio13_xy,Bio14_xy,Bio15_xy,Bio16_xy,Bio17_xy,Bio18_xy,Bio19_xy,AM_solarRad_
xy,AM_windSpeed_xy,AM_vap_xy,Altitude_xy,AM_humidity_xy,AM_evapo_xy,Bowen_Feb_April_xy)

subsets <- c(1:26) #max number of variables
results_rfe <- rfe(predictor, d2H_proj_set$d2H.obs, sizes=subsets, rfeControl=control)

print(results_rfe)
## Recursive feature selection
## Outer resampling method: Cross-Validated (10 fold)
## Resampling performance over subset size:
##  Variables  RMSE Rsquared   MAE RMSESD RsquaredSD MAESD Selected
##          1 16.81   0.2363 12.92  1.922     0.1998 1.157
##          2 15.43   0.3157 11.78  3.139     0.2062 2.409
##          3 15.23   0.3148 11.73  3.136     0.2081 2.397
##          4 15.12   0.3247 11.66  3.095     0.2144 2.222
##          5 15.07   0.3259 11.66  3.164     0.2285 2.324        *
##          6 15.10   0.3261 11.62  3.309     0.2323 2.388
##          7 15.17   0.3183 11.74  3.263     0.2294 2.312
##          8 15.17   0.3174 11.73  3.310     0.2261 2.345
##          9 15.19   0.3191 11.79  3.314     0.2285 2.337
##         10 15.18   0.3181 11.73  3.314     0.2246 2.359
## The top 5 variables (out of 5):
##    Bowen_Feb_April_xy, Bio12_xy, AM_solarRad_xy, Bio7_xy, Bio18_xy

predictors(results_rfe)
## [1] "Bowen_Feb_April_xy" "Bio12_xy"           "AM_solarRad_xy"
## [4] "Bio7_xy"            "Bio18_xy"
```

3- Plot results

```
library(ggplot2)
ggplot(results_rfe, aes(x = Variables, y = Rsquared)) +
  geom_point(colour="black",size=2) +
  xlab("Variables") +
  ylab("RMSE (Cross-Validation)") +
  theme_classic()
```

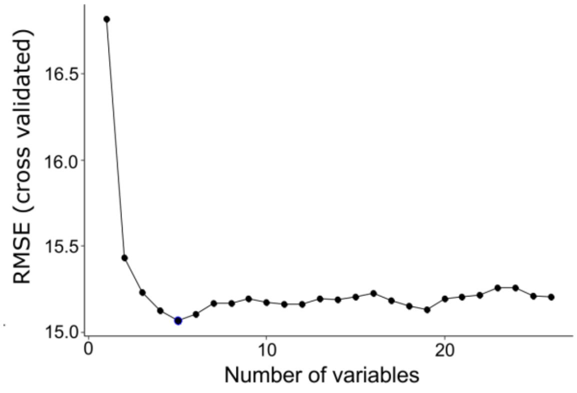

4- Exclude all useless features pointed by the RFE method

```
selected<-data.frame(ID,Long,Lat,d2H.obs,Bio7_xy,Bio12_xy,Bio18_xy,AM_solarRad_xy,Bowen_Feb_April_
xy)
```

Third Step - Prepare data and run Random Forest

1- Dataset partition in training and testing data

Useful link to understand Random Forest: https://www.listendata.com/2014/11/random-forest-with-r.html

```
set.seed(123)
inTrain <- createDataPartition(y=selected$d2H.obs, p=0.80, list=FALSE)
training <- selected[inTrain,];
testing <- selected[-inTrain,];

write.csv(training,file="training.csv")
write.csv(testing,file="testing.csv")
```

2- Random Forest Model

```
library(randomForest)
library(caret)
fitControl <- trainControl(
  method = "repeatedcv",
  number = 10,
  repeats = 5,
  verboseIter=FALSE ,
  returnResamp="final",
  savePredictions="all")

set.seed(123)
head(training)

##        ID   Long    Lat d2H.obs Bio7_xy Bio12_xy Bio18_xy AM_solarRad_xy
## 1 RDA001 -41.04 -11.61  -54.86   14.41   654.39   200.54       17002.81
## 2 RDA002 -41.04 -11.61  -47.86   14.41   654.39   200.54       17002.81
## 4 RDA004 -40.74 -16.30  -56.65   15.53   794.06   245.26       15819.48
## 5 RDA005 -42.69 -16.91  -45.06   17.47   850.23   304.14       15616.53
## 6 RDA006 -42.69 -16.91  -65.72   17.47   850.23   304.14       15616.53
## 7 RDA007 -41.35 -13.39  -45.51   15.97   795.64   278.70       16383.87
##
```

```
 Bowen_Feb_April_xy
## 1              -20.68
## 2              -20.68
## 4              -18.57
## 5              -32.01
## 6              -32.01
## 7              -22.57
```

```
training1<-training[,4:9]
bestmtry <- tuneRF(training1[,-1], training$d2H.obs, stepFactor=3, improve=2, ntree=500)
```

```
## mtry = 1 OOB error = 244.5054
## Searching left ...
## Searching right ...
## mtry = 3     OOB error = 246.2862
## -0.007283265 2
```

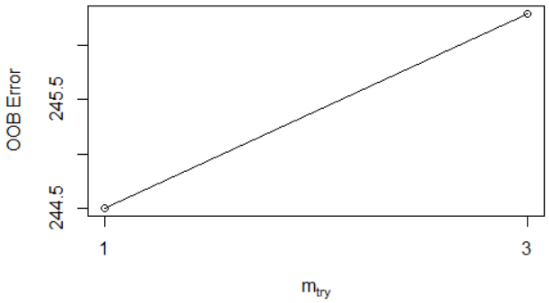


```
mtry <- 3
tunegrid <- expand.grid(.mtry=mtry)
RF1 <- train(d2H.obs ~ ., data = training1, method = "rf", tuneGrid=tunegrid, trControl= fitContro
l, importance = TRUE)
varImp(RF1)
```

```
## rf variable importance
##                     Overall
## Bio12_xy             100.00
## Bowen_Feb_April_xy    64.77
## Bio18_xy              53.78
## AM_solarRad_xy        16.00
## Bio7_xy                0.00
```

```
imp<-varImp(RF1$finalModel)
imp$varnames <- rownames(imp) # row names to column
rownames(imp) <- NULL

ggplot(imp, aes(x=reorder(varnames, Overall), y=Overall)) +
  geom_point() +
  geom_segment(aes(x=varnames,xend=varnames,y=0,yend=Overall)) +
  ylab("%IncMSE") +
  xlab("Variable Name") +
  coord_flip()+
  theme_bw()+
  theme(panel.grid = element_blank(),axis.title = element_text(size = 14),axis.text = element_text
(size = 12))
```

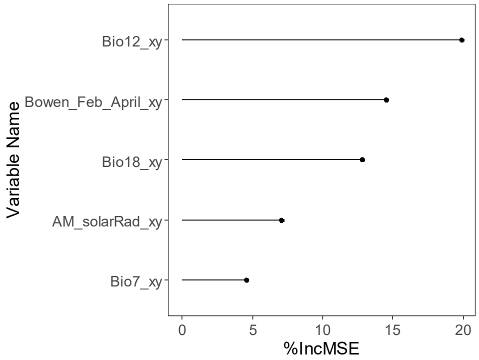

3- Explore results

```
library(pdp)
library(ggplot2)
library(Rmisc)

Mean Anual Precipitation (Bio12)
plot.Bio12  <- partial(RF1, pred.var = c("Bio12_xy"), chull = TRUE, rug = TRUE, plot = TRUE,plot.e
ngine = "ggplot2") +
  xlab(expression ("Bio12:Mean Annual Precipitation")) +
  ylab(expression (delta^{2}~H[feather]~"(\u2030)"))+
  ggtitle("A")+
  theme_bw()+
  theme(panel.grid = element_blank(),axis.title = element_text(size = 14),
        axis.text = element_text(size = 12),
        plot.title=element_text( hjust=.01, vjust=-1))

plot.Isotop.Prec  <- partial(RF1, pred.var = c("Bowen_Feb_April_xy"), chull = TRUE, rug = TRUE, pl
ot = TRUE,plot.engine = "ggplot2") +
  xlab(expression (delta^{2}~H[prec_Feb-April]~"(\u2030)")) +
  ylab(expression (delta^{2}~H[feather]~"(\u2030)"))+
  ggtitle("B")+
  theme_bw()+
  theme(panel.grid = element_blank(),axis.title = element_text(size = 14),
        axis.text = element_text(size = 12),
        plot.title=element_text( hjust=.01, vjust=-1))

plot.Bio18  <- partial(RF1, pred.var = c("Bio18_xy"), chull = TRUE, rug = TRUE, plot = TRUE,plot.e
ngine = "ggplot2") +
  xlab(expression ("Bio18:Precipitation at the warmest quarter")) +
  ylab(expression (delta^{2}~H[feather]~"(\u2030)"))+
  ggtitle("C")+
  theme_bw()+
  theme(panel.grid = element_blank(),axis.title = element_text(size = 14),
        axis.text = element_text(size = 12),
        plot.title=element_text( hjust=.01, vjust=-1))

plot.Solar  <- partial(RF1, pred.var = c("AM_solarRad_xy"), chull = TRUE, rug = TRUE, plot = TRUE,
plot.engine = "ggplot2") +
  xlab(expression ("Mean Annual Solar Radiation")) +
  ylab(expression (delta^{2}~H[feather]~"(\u2030)"))+
  ggtitle("D")+
  theme_bw()+
  theme(panel.grid = element_blank(),axis.title = element_text(size = 14),
        axis.text = element_text(size = 12),
        plot.title=element_text( hjust=.01, vjust=-1))
```

```
plot.Bio7  <- partial(RF1, pred.var = c("Bio7_xy"), chull = TRUE, rug = TRUE, plot = TRUE,plot.eng
ine = "ggplot2") +
  xlab(expression ("Bio7:Annual Temperature Range")) +
  ylab(expression (delta^{2}~H[feather]~"(\u2030)"))+
  ggtitle("E")+
  theme_bw()+
  theme(panel.grid = element_blank(),axis.title = element_text(size = 14),
        axis.text = element_text(size = 12),
        plot.title=element_text( hjust=.01, vjust=-1))
plot.Bio12
plot.Isotop.Prec
plot.Bio18
plot.Solar
plot.Bio7
```

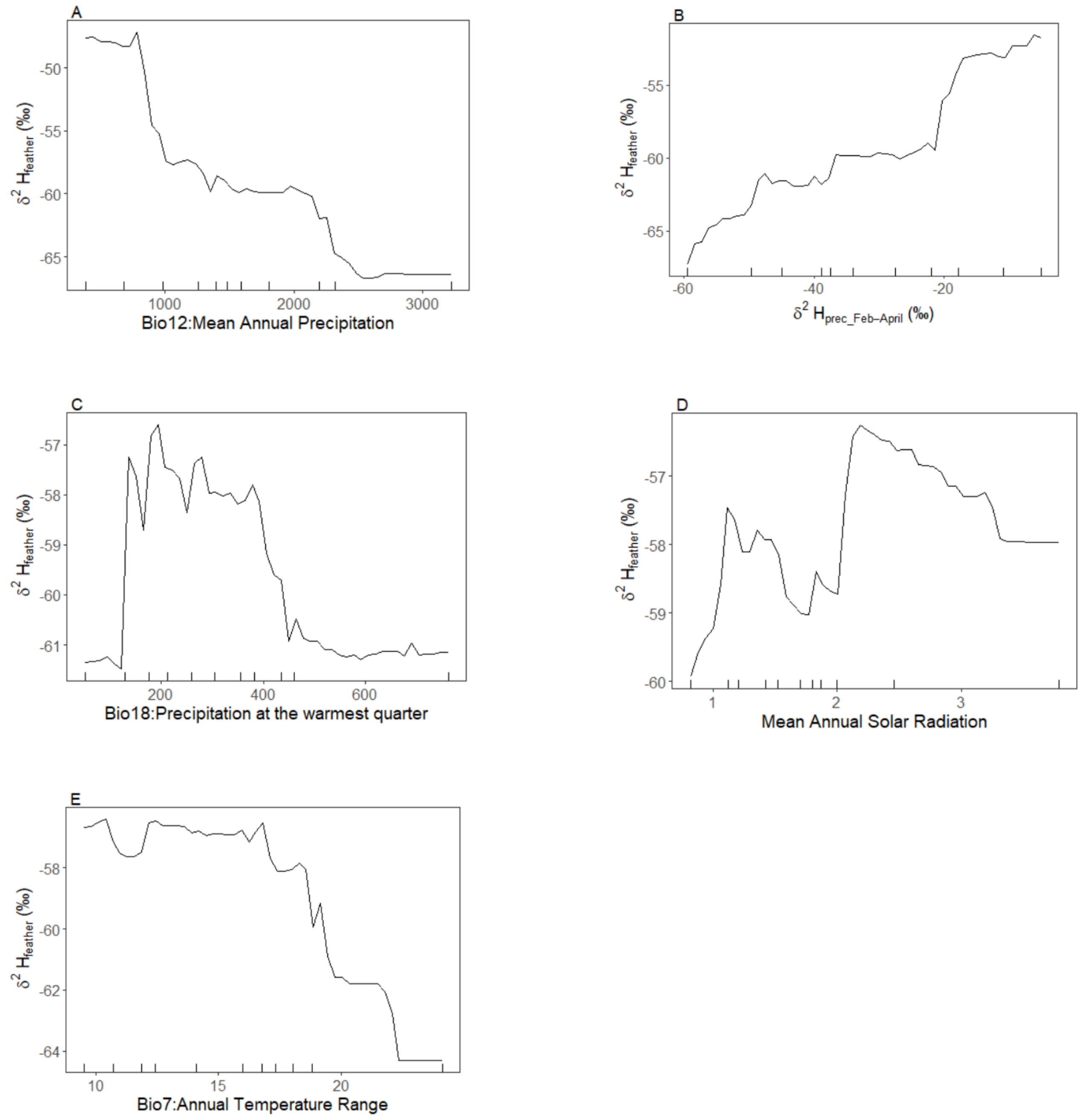

Fourth Step – Model validation

```
pred<-as.data.frame(predict(RF1,training1))
comp.obspre.rf<-data.frame(pred$`predict(RF1, training1)`,training1$d2H.obs)

colnames(comp.obspre.rf)<-c("d2h.predicted2","d2H.observed2")
```

1- Model validation with 10-fold cross-validation

```
comp.10fold<-data.frame(RF1$pred$pred,RF1$pred$obs)
is.num <- sapply(comp.10fold, is.numeric)
comp.10fold[is.num] <- lapply(comp.10fold[is.num], round, 2)

lm2<-lm(RF1$pred$obs~RF1$pred$pred)
summary(lm2)
##
## Call:
## lm(formula = RF1$pred$obs ~ RF1$pred$pred)
##
## Residuals:
##     Min      1Q    Median      3Q     Max
## -42.879    -9.175   0.922    8.824   48.082
##
## Coefficients:
##                Estimate Std. Error t value Pr(>|t|)
## (Intercept)   -14.25101    2.73382  -5.213 2.38e-07 ***
## RF1$pred$pred   0.75408    0.04588  16.434  < 2e-16 ***
## ---
## Signif. codes:  0 '***' 0.001 '**' 0.01 '*' 0.05 '.' 0.1 ' ' 1
##
## Residual standard error: 15.24 on 778 degrees of freedom
## Multiple R-squared:  0.2577, Adjusted R-squared:  0.2567
## F-statistic: 270.1 on 1 and 778 DF,  p-value: < 2.2e-16
RMSE<-sqrt(mean(lm2$residual^2))
[1] 15.21848
```

```
MAE<-mean(lm2$residual^2)
## [1] 231.21848
ggplot(comp.10fold, aes(x = RF1.pred.pred, y = RF1.pred.obs)) + geom_point(alpha = 1/2) +
  geom_abline(intercept = 0, slope = 1,colour = "grey") +
  stat_smooth(method = lm, colour = "black", se = T) +
  scale_x_continuous(limits=c(-100, 0)) +
  scale_y_continuous(limits=c(-100, 0)) +
  xlab(expression (Predicted~delta^{2}~H["feather"]~"(\u2030)")) +
  ylab(expression (Observed~delta^{2}~H["feather"]~"(\u2030)")) +
  ggtitle("A")+
  theme_classic()+
  theme(panel.grid = element_blank(),axis.title = element_text(size = 14),
        axis.text = element_text(size = 12),
        plot.title=element_text( hjust=.01, vjust=-7))
```

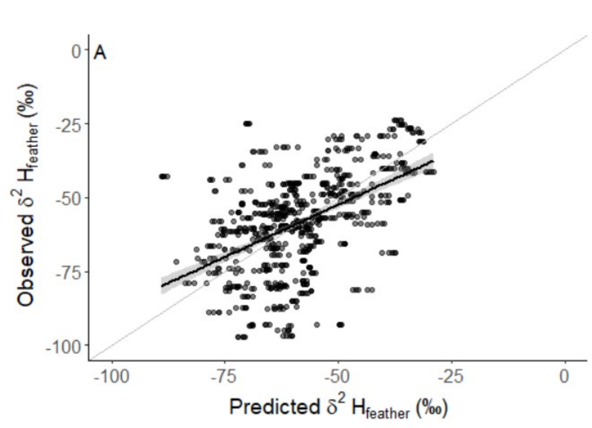

2- Model validation with testing data

Run the model again

```
library(randomForest)
set.seed(123)testing1<-testing[,4:9]
bestmtry <- tuneRF(training1[,-1], training$d2H.obs, stepFactor=3, improve=2, ntree=500)

mtry <- 3
tunegrid <- expand.grid(.mtry=mtry)
RF2 <- train(d2H.obs ~ ., data = testing1, method = "rf", tuneGrid=tunegrid,trControl= fitControl,
importance = TRUE)
varImp(RF2)

## rf variable importance
##
##                    Overall
## Bowen_Feb_April_xy  100.00
## Bio7_xy              46.11
## Bio12_xy             22.84
## AM_solarRad_xy        6.76
## Bio18_xy              0.00

pred<-as.data.frame(predict(RF2,testing1))
comp.obspre.rf2<-data.frame(pred$`predict(RF2, testing1)`,testing1$d2H.obs)
colnames(comp.obspre.rf2)<-c("d2h.predicted2","d2H.observed2")


lm2<-lm(comp.obspre.rf2$d2H.observed2~comp.obspre.rf2$d2h.predicted2)
summary (lm2)

##
## Call:
## lm(formula = comp.obspre.rf2$d2H.observed2~comp.obspre.rf2$d2h.predicted2)
##
## Residuals:
##     Min      1Q  Median      3Q     Max
##  -9.9585 -4.0266 -0.0913  4.7800 16.6118
##
## Coefficients:
##                                    Estimate Std. Error t value Pr(>|t|)
## (Intercept)                       10.78576    3.96692   2.719   0.0102***
## comp.obspre.rf$testing1.d2H.obs    1.18525    0.06454  18.365   <2e-16***
## ---
```

```
## Signif. codes:  0 '***' 0.001 '**' 0.01 '*' 0.05 '.' 0.1 ' ' 1
##
## Residual standard error: 6.064 on 34 degrees of freedom
## Multiple R-squared:  0.9084, Adjusted R-squared:  0.9057
## F-statistic: 337.3 on 1 and 34 DF,p-value: < 2.2e-16

MAE<-mean(lm2$residual^2)

## [1] 34.73267

RMSE<-sqrt(mean(lm2$residual^2))

## [1] 5.893443


ggplot(comp.obspre.rf, aes(x = d2h.predicted, y = d2H.observed))  +                         geom_po
int(alpha = 1/2) +
  geom_abline(intercept = 0, slope = 1, colour = "grey") +
  stat_smooth(method = lm, colour = "black", se = T) +
  scale_x_continuous(limits=c(-100, 0)) +
  scale_y_continuous(limits=c(-100, 0)) +
   xlab(expression (Predicted~delta^{2}~H["feather"]~"(\u2030)")) +   ylab(expression (Observed~de
lta^{2}~H["feather"]~"(\u2030)")) +
  ggtitle("B")+
  theme_classic()+ # adciona tema "Black and White"
  theme(panel.grid = element_blank(),axis.title = element_text(size = 14),axis.text = element_text
(size = 12),
        plot.title=element_text( hjust=.01, vjust=-7))
```

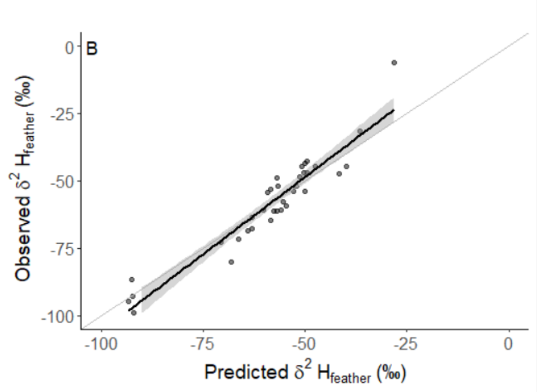

Fifth Step – Spatial prediction

1- Open raster

```
library(raster)
Bio7<-raster("Bio7_Temp_annual_range_2007_2018.grd")
Bio12<-raster("Bio12_Annual_precipitation_2007_2018.grd")
Bio18<-raster("Bio18_Prec_warmest_quarter_2007_2018.grd")
AM_solarRad<-raster("R_Mean_Annual_srad_resampled.grd")
Bowen_Feb_April<-raster("Prec_fev_abr_iso_Bowen-resampled.grd")
```

2- Stack raster for prediction

```
Brazil_stack<-stack(Bio7,Bio12,Bio18,AM_solarRad,Bowen_Feb_April)
names(Brazil_stack)<-c("Bio7_xy","Bio12_xy","Bio18_xy","AM_solarRad_xy","Bowen_Feb_April_xy")
```

3- Create a grid to apply model extent

```
sa.grid<-Bio7/Bio7
```

4- Run the same model, 20 times, to get the CV map and uncertainty

```
RF_1 <- train(d2H.obs ~ ., data = training1, method = "rf", tuneGrid=tunegrid,trControl= fitContro
l, importance = TRUE)
varImp(RF_1)
RF_pred1 <- predict(Brazil_stack, RF_1, ext=sa.grid, na.rm=TRUE, overwrite=TRUE, progress='text')
plot(RF_pred1)
writeRaster(RF_pred1, filename="RF_pred1.tif", format="GTiff", overwrite=TRUE)

RF_2 <- train(d2H.obs ~ ., data = training1, method = "rf", tuneGrid=tunegrid,trControl= fitContro
l, importance = TRUE)
varImp(RF_2)
RF_pred2 <- predict(Brazil_stack, RF_2, ext=sa.grid, na.rm=TRUE, overwrite=TRUE, progress='text')
writeRaster(RF_pred2, filename="RF_pred2.tif", format="GTiff", overwrite=TRUE)

RF_3 <- train(d2H.obs ~ ., data = training1, method = "rf", tuneGrid=tunegrid,trControl= fitContro
l, importance = TRUE)
varImp(RF_3)
RF_pred3 <- predict(Brazil_stack, RF_3, ext=sa.grid, na.rm=TRUE, overwrite=TRUE, progress='text')
writeRaster(RF_pred3, filename="RF_pred3.tif", format="GTiff", overwrite=TRUE)

RF_4 <- train(d2H.obs ~ ., data = training1, method = "rf", tuneGrid=tunegrid,trControl= fitContro
l, importance = TRUE)
varImp(RF_4)
RF_pred4 <- predict(Brazil_stack, RF_4, ext=sa.grid, na.rm=TRUE, overwrite=TRUE, progress='text')
writeRaster(RF_pred4, filename="RF_pred4.tif", format="GTiff", overwrite=TRUE)

RF_5 <- train(d2H.obs ~ ., data = training1, method = "rf", tuneGrid=tunegrid,trControl= fitContro
l, importance = TRUE)
varImp(RF_5)
RF_pred5 <- predict(Brazil_stack, RF_5, ext=sa.grid, na.rm=TRUE, overwrite=TRUE, progress='text')
writeRaster(RF_pred5, filename="RF_pred5.tif", format="GTiff", overwrite=TRUE)

RF_6 <- train(d2H.obs ~ ., data = training1, method = "rf", tuneGrid=tunegrid,trControl= fitContro
l, importance = TRUE)
varImp(RF_6)
RF_pred6 <- predict(Brazil_stack, RF_6, ext=sa.grid, na.rm=TRUE, overwrite=TRUE, progress='text')
writeRaster(RF_pred6, filename="RF_pred6.tif", format="GTiff", overwrite=TRUE)
```

```
RF_7 <- train(d2H.obs ~ ., data = training1, method = "rf", tuneGrid=tunegrid,trControl= fitContro
l, importance = TRUE)
varImp(RF_7)
RF_pred7 <- predict(Brazil_stack, RF_7, ext=sa.grid, na.rm=TRUE, overwrite=TRUE, progress='text')
writeRaster(RF_pred7, filename="RF_pred7.tif", format="GTiff", overwrite=TRUE)
```

```
RF_8 <- train(d2H.obs ~ ., data = training1, method = "rf", tuneGrid=tunegrid,trControl= fitContro
l, importance = TRUE)
varImp(RF_8)
RF_pred8 <- predict(Brazil_stack, RF_8, ext=sa.grid, na.rm=TRUE, overwrite=TRUE, progress='text')
```

```
writeRaster(RF_pred8, filename="RF_pred8.tif", format="GTiff", overwrite=TRUE)

RF_9 <- train(d2H.obs ~ ., data = training1, method = "rf", tuneGrid=tunegrid,trControl= fitContro
l, importance = TRUE)
varImp(RF_9)
RF_pred9 <- predict(Brazil_stack, RF_9, ext=sa.grid, na.rm=TRUE, overwrite=TRUE, progress='text')
writeRaster(RF_pred9, filename="RF_pred9.tif", format="GTiff", overwrite=TRUE)

RF_10 <- train(d2H.obs ~ ., data = training1, method = "rf", tuneGrid=tunegrid,trControl= fitContr
ol, importance = TRUE)
varImp(RF_10)
RF_pred10 <- predict(Brazil_stack, RF_10, ext=sa.grid, na.rm=TRUE, overwrite=TRUE, progress='text'
)
writeRaster(RF_pred10, filename="RF_pred10.tif", format="GTiff", overwrite=TRUE)

RF_11 <- train(d2H.obs ~ ., data = training1, method = "rf", tuneGrid=tunegrid,trControl= fitContr
ol, importance = TRUE)
varImp(RF_11)
RF_pred11 <- predict(Brazil_stack, RF_11, ext=sa.grid, na.rm=TRUE, overwrite=TRUE, progress='text'
)
writeRaster(RF_pred11, filename="RF_pred11.tif", format="GTiff", overwrite=TRUE)

RF_12 <- train(d2H.obs ~ ., data = training1, method = "rf", tuneGrid=tunegrid,trControl= fitContr
ol, importance = TRUE)
varImp(RF_12)
RF_pred12 <- predict(Brazil_stack, RF_12, ext=sa.grid, na.rm=TRUE, overwrite=TRUE, progress='text'
)
writeRaster(RF_pred12, filename="RF_pred12.tif", format="GTiff", overwrite=TRUE)

RF_13 <- train(d2H.obs ~ ., data = training1, method = "rf", tuneGrid=tunegrid,trControl= fitContr
ol, importance = TRUE)
varImp(RF_13)
RF_pred13 <- predict(Brazil_stack, RF_13, ext=sa.grid, na.rm=TRUE, overwrite=TRUE, progress='text'
)
writeRaster(RF_pred13, filename="RF_pred13.tif", format="GTiff", overwrite=TRUE)

RF_14 <- train(d2H.obs ~ ., data = training1, method = "rf", tuneGrid=tunegrid,trControl= fitContr
ol, importance = TRUE)
varImp(RF_14)
RF_pred14 <- predict(Brazil_stack, RF_14, ext=sa.grid, na.rm=TRUE, overwrite=TRUE, progress='text'
)
writeRaster(RF_pred14, filename="RF_pred14.tif", format="GTiff", overwrite=TRUE)

RF_15 <- train(d2H.obs ~ ., data = training1, method = "rf", tuneGrid=tunegrid,trControl= fitContr
ol, importance = TRUE)
varImp(RF_15)
RF_pred15 <- predict(Brazil_stack, RF_15, ext=sa.grid, na.rm=TRUE, overwrite=TRUE, progress='text'
)
writeRaster(RF_pred15, filename="RF_pred15.tif", format="GTiff", overwrite=TRUE)

RF_16 <- train(d2H.obs ~ ., data = training1, method = "rf", tuneGrid=tunegrid,trControl= fitContr
ol, importance = TRUE)
varImp(RF_16)
RF_pred16 <- predict(Brazil_stack, RF_16, ext=sa.grid, na.rm=TRUE, overwrite=TRUE, progress='text'
)
writeRaster(RF_pred16, filename="RF_pred16.tif", format="GTiff", overwrite=TRUE)
```

```
RF_17 <- train(d2H.obs ~ ., data = training1, method = "rf", tuneGrid=tunegrid,trControl= fitContr
ol, importance = TRUE)
varImp(RF_17)
RF_pred17 <- predict(Brazil_stack, RF_17, ext=sa.grid, na.rm=TRUE, overwrite=TRUE, progress='text'
)
writeRaster(RF_pred17, filename="RF_pred17.tif", format="GTiff", overwrite=TRUE)
```

```
RF_18 <- train(d2H.obs ~ ., data = training1, method = "rf", tuneGrid=tunegrid,trControl= fitContr
ol, importance = TRUE)
varImp(RF_18)
RF_pred18 <- predict(Brazil_stack, RF_18, ext=sa.grid, na.rm=TRUE, overwrite=TRUE, progress='text'
)
writeRaster(RF_pred18, filename="RF_pred18.tif", format="GTiff", overwrite=TRUE)
```

```
RF_19 <- train(d2H.obs ~ ., data = training1, method = "rf", tuneGrid=tunegrid,trControl= fitContr
ol, importance = TRUE)
varImp(RF_19)
RF_pred19 <- predict(Brazil_stack, RF_19, ext=sa.grid, na.rm=TRUE, overwrite=TRUE, progress='text'
)
writeRaster(RF_pred19, filename="RF_pred19.tif", format="GTiff", overwrite=TRUE)
```

```
RF_20 <- train(d2H.obs ~ ., data = training1, method = "rf", tuneGrid=tunegrid,trControl= fitContr
ol, importance = TRUE)
varImp(RF_20)
RF_pred20 <- predict(Brazil_stack, RF_20, ext=sa.grid, na.rm=TRUE, overwrite=TRUE, progress='text'
)
writeRaster(RF_pred20, filename="RF_pred20.tif", format="GTiff", overwrite=TRUE)
```

RF1

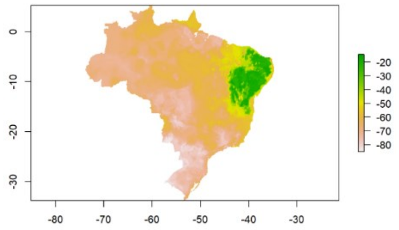


```
## rf variable importance
##                      Overall
## Bio12_xy             100.000
## Bio18_xy              60.293
## Bowen_Feb_April_xy    57.119
## AM_solarRad_xy         5.434
## Bio7_xy                0.000
```

RF2

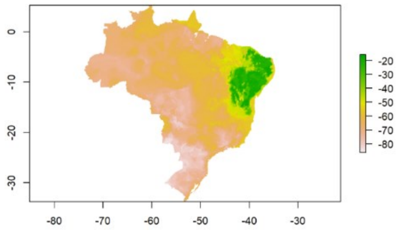


```
## rf variable importance
##                      Overall
## Bio12_xy             100.000
## Bowen_Feb_April_xy    71.420
## Bio18_xy              65.150
## AM_solarRad_xy        11.560
## Bio7_xy                0.000
```

RF3

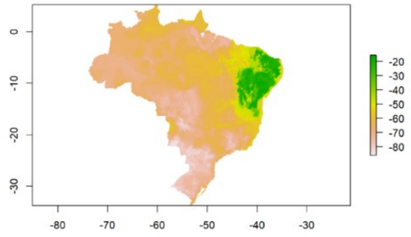


```
## rf variable importance
##                      Overall
## Bio12_xy             100.000
## Bowen_Feb_April_xy    59.900
## Bio18_xy              50.310
## AM_solarRad_xy        26.700
## Bio7_xy                0.000
```

RF4

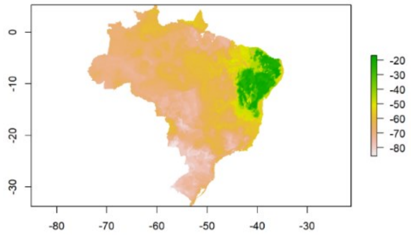


```
## rf variable importance
##                     Overall
## Bio12_xy            100.000
## Bowen_Feb_April_xy   70.459
## Bio18_xy             58.604
## AM_solarRad_xy        1.396
## Bio7_xy               0.000
```

RF5

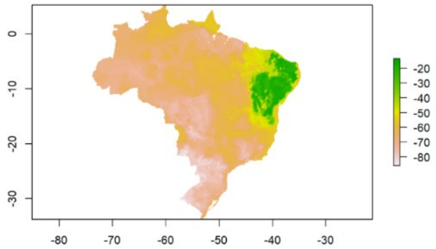


```
## rf variable importance
##                     Overall
## Bio12_xy            100.000
## Bowen_Feb_April_xy   58.345
## Bio18_xy             44.127
## AM_solarRad_xy        9.549
## Bio7_xy               0.000
```

RF6

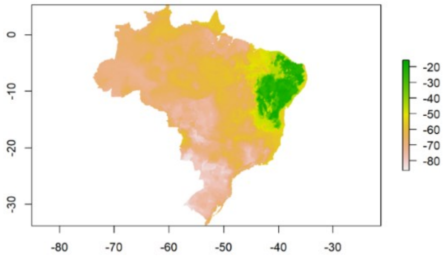


```
## rf variable importance
##                     Overall
## Bio12_xy            100.000
## Bio18_xy             51.585
## Bowen_Feb_April_xy   51.413
## AM_solarRad_xy        7.174
## Bio7_xy               0.000
```

RF7

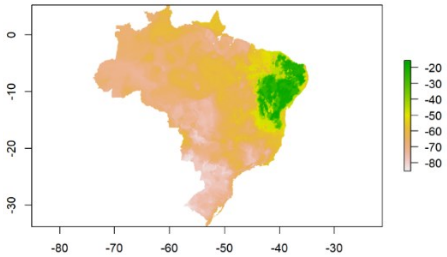


```
## rf variable importance
##                     Overall
## Bio12_xy            100.000
## Bowen_Feb_April_xy   58.446
## Bio18_xy             46.343
## AM_solarRad_xy        9.929
## Bio7_xy               0.000
```

RF8

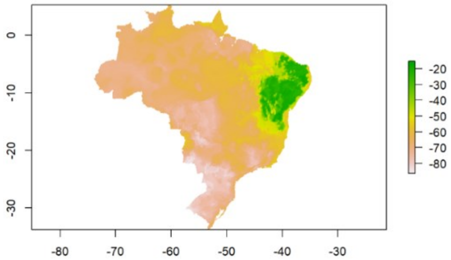


```
## rf variable importance
##                     Overall
## Bio12_xy            100.000
## Bowen_Feb_April_xy   60.585
## Bio18_xy             57.605
## AM_solarRad_xy        1.716
## Bio7_xy               0.000
```

RF9

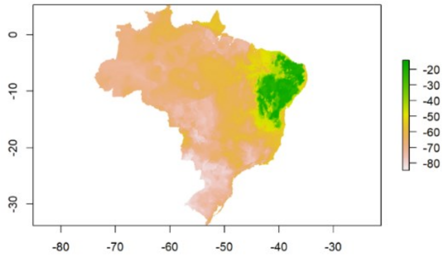


```
## rf variable importance
##                     Overall
## Bio12_xy            100.000
## Bowen_Feb_April_xy   60.973
## Bio18_xy             51.273
## Bio7_xy               1.202
## AM_solarRad_xy        0.000
```

RF10

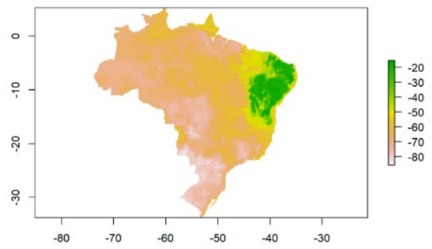


```
## rf variable importance
##                      Overall
## Bio12_xy            100.0000
## Bio18_xy             58.3332
## Bowen_Feb_April_xy   51.7474
## AM_solarRad_xy        0.7305
## Bio7_xy               0.0000
```

RF11

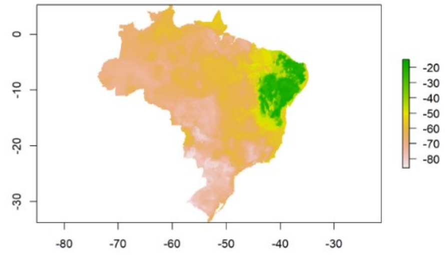


```
## rf variable importance
##                    Overall
## Bio12_xy            100.00
## Bowen_Feb_April_xy   55.69
## Bio18_xy             51.76
## AM_solarRad_xy       21.86
## Bio7_xy               0.00
```

RF12

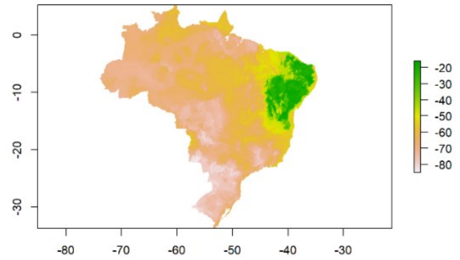


```
## rf variable importance
##                    Overall
## Bio12_xy           100.000
## Bio18_xy            71.533
## Bowen_Feb_April_xy  60.920
## Bio7_xy              1.021
## AM_solarRad_xy       0.000
```

RF13

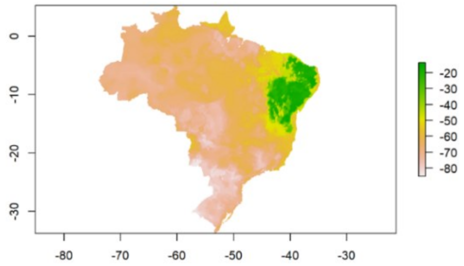


```
## rf variable importance
##                    Overall
## Bio12_xy            100.00
## Bowen_Feb_April_xy   65.79
## Bio18_xy             56.00
## AM_solarRad_xy       25.87
## Bio7_xy               0.00
```

RF14

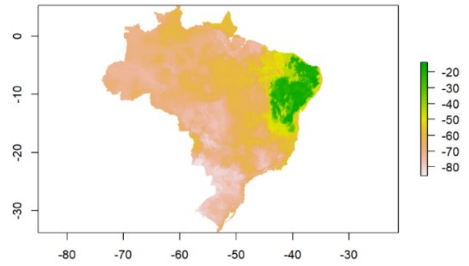


```
## rf variable importance
##                    Overall
## Bio12_xy            100.00
## Bio18_xy             46.76
## Bowen_Feb_April_xy   46.63
## AM_solarRad_xy        4.26
## Bio7_xy               0.00
```

RF15

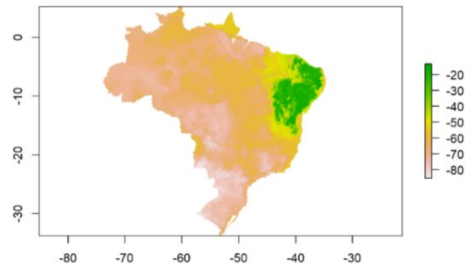


```
## rf variable importance
##                    Overall
## Bio12_xy            100.00
## Bowen_Feb_April_xy   66.21
## Bio18_xy             61.78
## AM_solarRad_xy       22.42
## Bio7_xy               0.00
```

RF16

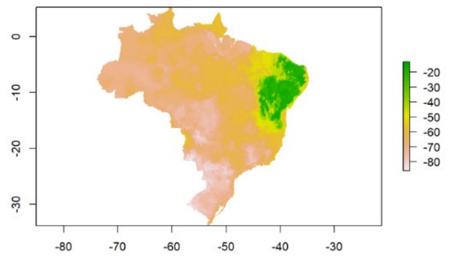


```
## rf variable importance
##                      Overall
## Bio12_xy             100.00
## Bowen_Feb_April_xy    62.19
## Bio18_xy              48.67
## AM_solarRad_xy        17.05
## Bio7_xy                0.00
```

RF17

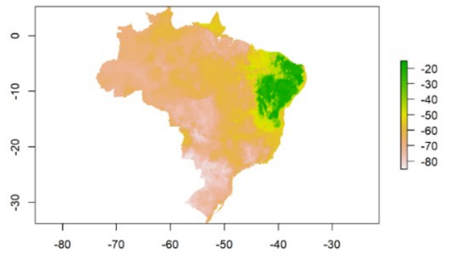


```
## rf variable importance
##                      Overall
## Bio12_xy             100.00
## Bowen_Feb_April_xy    77.01
## Bio18_xy              64.63
## AM_solarRad_xy        29.12
## Bio7_xy                0.0
```

RF18

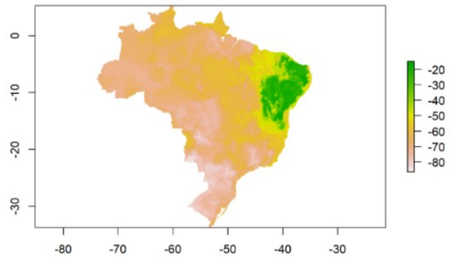


```
## rf variable importance
##                      Overall
## Bio12_xy             100.000
## Bowen_Feb_April_xy    50.324
## Bio18_xy              45.156
## AM_solarRad_xy         7.857
## Bio7_xy                0.000
```

RF19

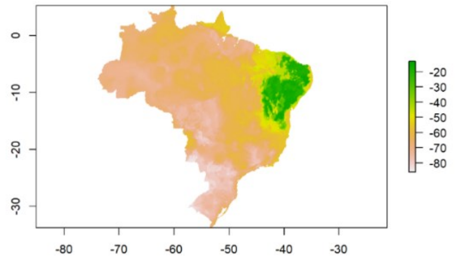


```
## rf variable importance
##                      Overall
## Bio12_xy             100.00
## Bio18_xy              69.42
## Bowen_Feb_April_xy    63.84
## AM_solarRad_xy        23.93
## Bio7_xy                0.00
```

RF20

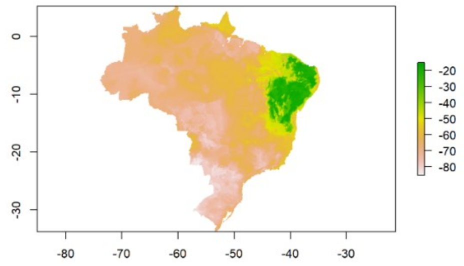


```
## rf variable importance
##                      Overall
## Bio12_xy             100.00
## Bio18_xy              65.32
## Bowen_Feb_April_xy    64.73
## AM_solarRad_xy        12.36
## Bio7_xy                0.00
```

6- Calculate an average model

```
RFpred.stack <- stack(RFpred1, RFpred2,RFpred3,RFpred4,RFpred5,
                      RFpred6,RFpred7,RFpred8,RFpred9,RFpred10,
                      RFpred11, RFpred12,RFpred13,RFpred14,RFpred15,
                     RFpred16,RFpred17,RFpred18,RFpred19,RFpred20)

qt_rfpred<-quantile(RFpred.stack, probs = c(0.05, 0.95), type=7,names = FALSE)
plot(qt_rfpred)
```

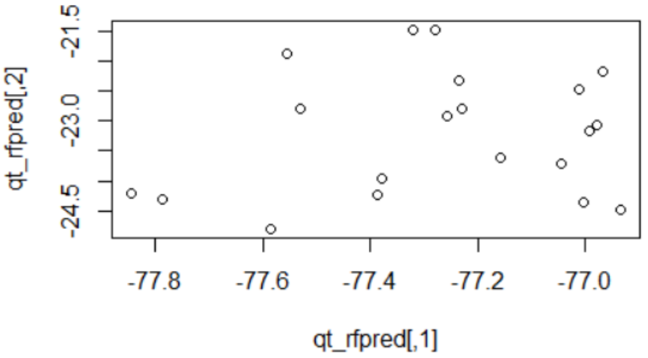


```
RFpred_mean <- overlay(RFpred1, RFpred2,RFpred3,RFpred4,RFpred5,
                       RFpred6,RFpred7,RFpred8,RFpred9,RFpred10,
                       RFpred11, RFpred12, RFpred13,RFpred14,RFpred15,
                     RFpred16,RFpred17,RFpred18,RFpred19,RFpred20,fun='mean')


plot(RFpred_mean,main="Final Map (based on the mean values of 20 models)")
```

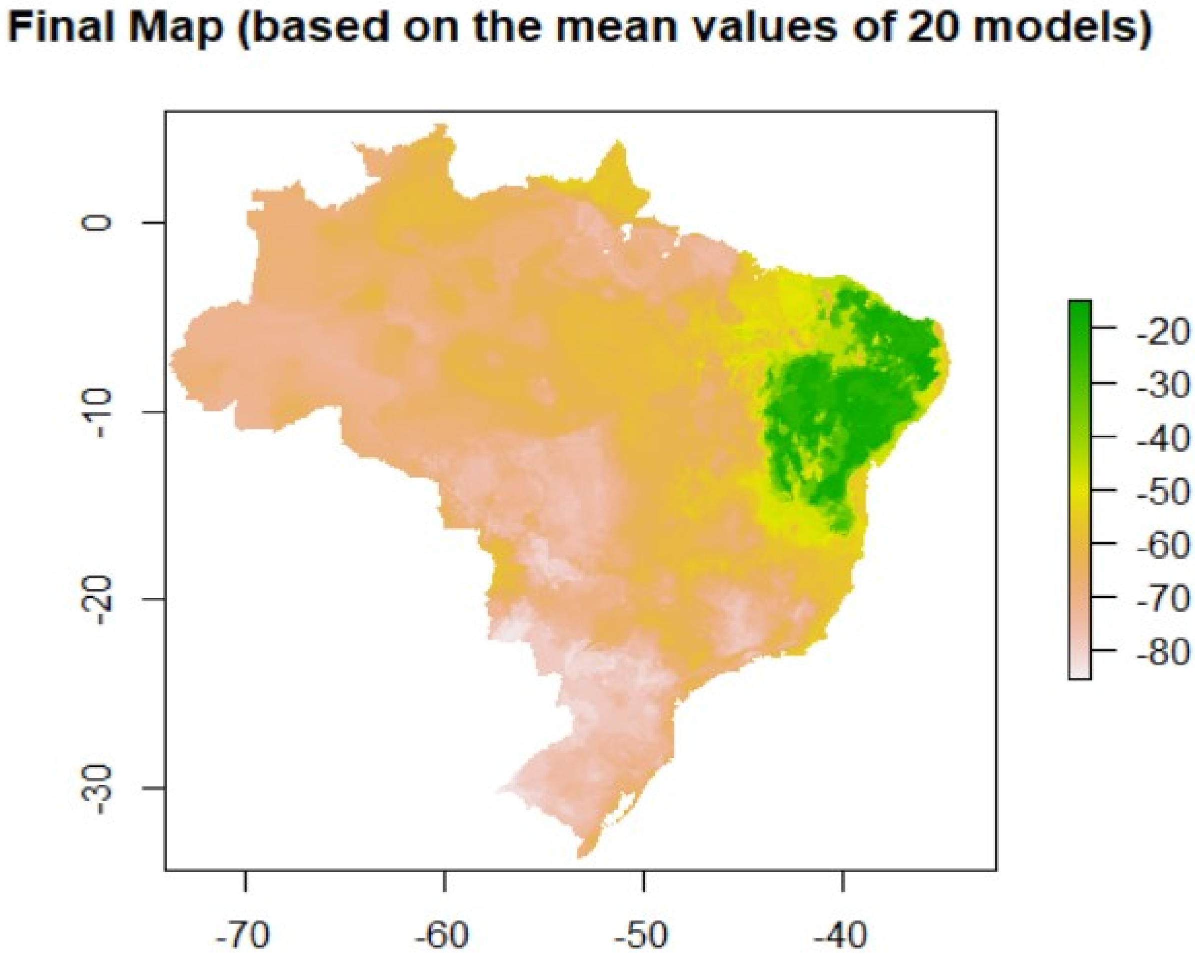

Supplement: S4 File — First step- Prepare data. R code to input raster and observations, and extract values. Including S5 Table in S4 File with extracted values; Second step- Selection of variables using Recursive Feature Elimination–RFE. R code to run RFE and plot results; Third step- Prepare data and run Random Forest (RF). R code to run RF and explore results with partial plots; Fourth step- Model validation. R code to validate the model and plot results; Fifth step- Spatial prediction. R code to apply modeled values into an isoscape, calculate method uncertainty, mean, standard deviation and coefficient of variation. (PDF) [file pone.0271573.s005.pdf]
